# A Zinc Catalyzed C(sp^3^)−C(sp^2^) Suzuki–Miyaura Cross‐Coupling Reaction Mediated by Aryl‐Zincates

**DOI:** 10.1002/chem.201704170

**Published:** 2017-10-19

**Authors:** Richard J. Procter, Jay J. Dunsford, Philip J. Rushworth, David G. Hulcoop, Richard A. Layfield, Michael J. Ingleson

**Affiliations:** ^1^ School of Chemistry The University of Manchester Manchester M13 9PL UK; ^2^ Research and Development GlaxoSmithKline Gunnelswood Road Stevenage SG1 2NY UK

**Keywords:** alkylation, boron, cross-coupling, transmetallation, zincate

## Abstract

The Suzuki–Miyaura (SM) reaction is one of the most important methods for C−C bond formation in chemical synthesis. In this communication, we show for the first time that the low toxicity, inexpensive element zinc is able to catalyze SM reactions. The cross‐coupling of benzyl bromides with aryl borates is catalyzed by ZnBr_2_, in a process that is free from added ligand, and is compatible with a range of functionalized benzyl bromides and arylboronic acid pinacol esters. Initial mechanistic investigations indicate that the selective in situ formation of triaryl zincates is crucial to promote selective cross‐coupling reactivity, which is facilitated by employing an arylborate of optimal nucleophilicity.

The selective formation of carbon–carbon bonds is arguably the most important transformation in synthetic chemistry. Among the most widely used C−C bond forming reactions is the Suzuki–Miyaura (SM) cross coupling reaction,^1^, ^2^ which is even utilized on large scale in industry.^3^ This powerful method couples a boron based organic nucleophile with an organic electrophile, typically catalyzed by Pd or Ni compounds.^4^ Recently, catalysts based on other metals, particularly less toxic metals (relative to Pd/Ni),^5^ for example, copper^6^ and iron (which has the lowest toxicity rating),^7^ that offer alternative reactivity profiles, and/or reduced costs, have gained increasing attention. However, zinc‐catalyzed SM reactions are, to the best of our knowledge, unknown. This is despite the attractive features of zinc which include: (i) low toxicity (in contrast to Ni compounds, zinc has the same low toxicity rating as iron),^5^ and (ii) relatively high abundance, thus zinc compounds are inexpensive and have low supply risk.^8^

The use of stoichiometric organozinc reagents in coupling reactions is well established, particularly the Pd‐catalyzed Negishi reaction.^9^ More recently, stoichiometric organozinc reagents have been used in coupling reactions that do not require transition‐metal catalysts.^10^ Of specific relevance to this work is the coupling of arylboronic acids with benzyl bromides in the presence of excess Et_2_Zn (Scheme 1 a). The proposed mechanism involves a zinc cation activating benzyl bromides for S_N_2 substitution.^11^ Diaryl zinc species have also been reacted with alkyl halides (including benzylic) to form C(sp^2^)−C(sp^3^) bonds in the abscence of a catalyst, provided the reaction was carried out in weakly‐coordinating aromatic solvents (Scheme 1 b).^12^ These recent developments, although notable, all use stoichiometric (or super‐stoichiometric) quantities of zinc reagents. The use of sub‐stoichiometric zinc compounds in C−C coupling is extremely rare, with the only example, to the best of our knowledge, being the coupling of alkyl Grignard reagents with α–hydroxy ester triflates catalyzed by ZnCl_2_ (Scheme 1 c).^13^ We sought to develop a catalytic zinc cross‐coupling reaction that uses arylboron nucleophiles. This requires an arylborate able to convert zinc halide by‐products from cross‐coupling back to arylzinc species that are effective for cross‐coupling with organic electrophiles. Herein, we realize this goal using readily accessible arylborate nucleophiles derived from arylboronic acid pinacol esters, with ZnBr_2_ proving to be an effective catalyst for coupling these arylborates with benzyl halides.

**Scheme 1:**
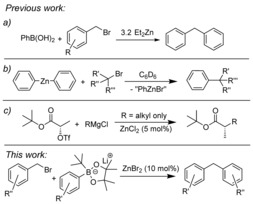
Zinc compounds in “catalyst‐free” coupling reactions.

Although neutral aryl boranes exchange aryl for alkyl on reaction with dialkylzinc reagents,^14^, ^15^ in order to transmetallate to zinc halides, more strongly nucleophilic arylboranes are required.^16^, ^17^ Attempts using arylboronic esters activated by alkoxides led to transfer of the alkoxide group to zinc in preference to the aryl group (see Figure S2). In contrast, the lithium borate, [*t*BuB(Pin)Ph]Li, **1 a**, selectively and rapidly (<30 min for complete consumption of **1 a**) transfers an aryl group to zinc dihalides (halide=Br or Cl) in ether solvents, as indicated by the formation of *tert*‐butylboronic acid pinacol ester (*t*BuBPin) by NMR spectroscopy (no PhBPin is observed precluding Zn−*t*Bu formation). An alternative pathway, rapid alkyl transfer from **1 a** to form ZnBu species, followed by rapid reaction of these with ArylBPin to form Aryl−Zn species is precluded, based on the slow reaction between ZnEt_2_ and ArylBpin (<5 % aryl transfer after 30 mins).

With an effective boron to ZnX_2_ transmetallating agent in hand, the transmetallation from boron to zinc in arene solvents was attempted, but it did not proceed significantly, since it was hindered by the low solubility of the aryl borate reagent and zinc halides. Arene solvents were essential in previous work on coupling stoichiometric Ar_2_Zn with alkyl halides, whereas in ether solvents, coupling was effectively quenched.^12^ By performing the transmetallation in cyclopentyl methyl ether (CPME) and then replacing CPME with benzene, the arylzinc product reacted with 3‐methoxybenzyl bromide (**2 a**) to generate the desired product (**3 a**) within 1 h at 20 °C. Subsequently, we found that using 10 mol % of zinc dihalide both steps can be performed in CPME, with heating enabling the cross‐coupling step (Scheme 2), albeit with a lower hetero/homo coupling ratio. In contrast, using ArylBPin/ZnEt_2_ mixtures with **2 a** as the electrophile led to minimal C(sp^2^)−C(sp^3^) cross‐coupling after 18 h at 60 °C.

**Scheme 2:**
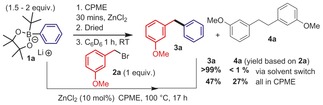
Top, cross coupling via a solvent switch, bottom cross coupling all in CPME.

The transmetallation and cross‐coupling steps in ether solvents were optimized using 4‐fluorobenzyl bromide, **2 b** (enabling quantitative in situ analysis by ^19^F NMR spectroscopy). This revealed that lower temperatures and other ether solvents resulted in high selectivity for heterocoupling (see Table 1). It is noteworthy that successful cross‐coupling was observed using dioxane as the solvent (entry 3), whereas ZnPh_2_ effectively does not undergo cross‐coupling with benzylbromides in dioxane, even at 60 °C over 18 h.^11^ Although highly selective cross‐coupling was observed in both dioxane and 2‐MeTHF, 2‐MeTHF was utilized for this study due to its superior safety profile. Control reactions were performed next to examine the possibility of trace‐metal catalysis.^18^ ZnBr_2_ obtained from multiple sources and of different purity (including 99.999 % purity) produced similar coupling outcomes. When the reaction was performed without ZnBr_2_, no **3 b** is formed, and only minor homocoupling (**4 b**) is observed (entry 6). Catalysis by trace copper or nickel impurities is disfavored on the basis of lower hetero‐/homocoupling selectivity (entries 7 and 8). FeBr_2_ was examined, and significant heterocoupling was observed (entry 9). However, FeBr_2_ is precluded as a “trace metal catalyst” in this chemistry due to significant reactivity differences compared to ZnBr_2_ (e.g. FeBr_2_ is an effective catalyst for heterocoupling using **1 a** and aryl Grignard reagents, whereas ZnBr_2_ does not couple aryl Grignard reagents with benzylbromides, see the Supporting information for further discussion). A Pd catalyst also gave high heterocoupling selectivity (entry 10). However, in the coupling of 4‐bromobenzylbromide (an electrophile containing both an aryl C−Br and benzylic C−Br bond) with **1 a**, ZnBr_2_ selectively couples through the benzylic carbon. In contrast, under identical conditions Pd(PPh_3_)_4_ cross‐couples through both the C(sp^2^)−Br and the C(sp^3^)−Br, thus precluding Pd impurities as the catalyst in this protocol (see Supporting Information). Finally, under these conditions MgBr_2_ led to no heterocoupling (entry 11). These results strongly support a zinc‐catalyzed cross‐coupling between **1 a** and **2 b**.

**Table 1:** Optimization and impurity catalysis controls.

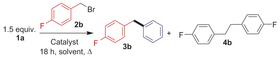					
Entry	Solvent	T [°C]	catalyst (mol %)	**3 b** [%]^[a]^	**4 b [**%]^[a]^
1	THF	60	ZnBr_2_ (10)	47	3
2	Benzene/THF 10:1	75	ZnBr_2_ (10)	70	3
3	Dioxane	60	ZnBr_2_ (10)	86	1
4	2‐MeTHF	60	ZnBr_2_ (10)	90	1
5	2‐MeTHF	80	ZnBr_2_ (10)	87	3
6	2‐MeTHF	60	No catalyst	0	16
7	2‐MeTHF	60	CuBr (13)	45	26
8	2‐MeTHF	60	NiBr_2_(PPh_3_)_2_ (3)	26	27
9	2‐MeTHF	60	FeBr_2_ (11)	84	8
10	2‐MeTHF	60	Pd(PPh_3_)_4_ (3)	93	2
11	2‐MeTHF	60	MgBr_2_ (10)	<1	12
12^[b]^	2‐MeTHF	60	ZnBr_2_ (10)	47	<1

[a] By ^19^F NMR spectroscopy and GCMS. [b] using Li[*n*BuB(Pin)Ph] (**5**) instead of **1 a**.

Optimization of the boron nucleophile also was explored briefly. When the *n*‐butyl congener of *t*‐butyl borate **1 a** (Li[*n*BuB(Pin)Ph], **5**) was used instead of **1 a**, **3 b** was cleanly formed, however, the reaction was slower than when **1 a** was used (compare entries 4 and 12. In contrast, using the alternative phenyl source Na[BPh_4_] led to minimal conversion to **3 b** after 18 h (<15 % **3 b**, see Supporting Information). Finally, 1.5 equivalents of **1 a** was found to improve cross‐coupling yields (lower equivalents of **1 a** did not lead to full consumption of the electrophile).

This zinc‐catalyzed cross‐coupling was compatible with electron‐withdrawing and donating groups (Table 2). It was also tolerant of halide, CF_3_, OCF_3_, alkyl, ether, thioether and heteroaryl groups, with excellent heterocoupling selectivity throughout. Electron‐withdrawing groups on the arylborate, for example, *p*‐(OCF_3_), are compatible but result in a slower reaction (only 30 % heterocoupling after 24 h), so they require longer reaction times. Although ketone and aldehyde functional groups proved to be incompatible, esters and acetals were both amenable to coupling. Benzyl chlorides reacted slower than the analogous bromides, while 2° benzyl electrophiles also reacted more slowly than 1° benzyl bromides, however, both are also viable substrates if longer reaction times are used. Bromodiphenylmethane and methylallyl bromide were also effectively cross‐coupled, however, octylbromide and cycloheptylbromide were not amenable. The formation of **3 o** was highly selective (>95 %) with minimal products from *cine*‐ or *tele*‐substitution observed, indicating that an organometallic *ipso*‐coupling process dominates.10g The observed scope is consistent with an S_N_2 mechanism, and the minor amounts of homocoupling observed (<5 %) is attributed to a zinc‐free reaction based on Table 1 entry 6. Radical scavengers such as 9,10‐dihydroanthracene (used in zinc‐mediated radical borylations),^19^ and styrene (a scavenger used in radical reactions involving arylborates)^20^ did not inhibit heterocoupling.

**Table 2:** Substrate scope for the zinc catalyzed C(sp^3^)−C(sp^2^) coupling.

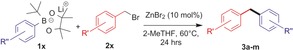
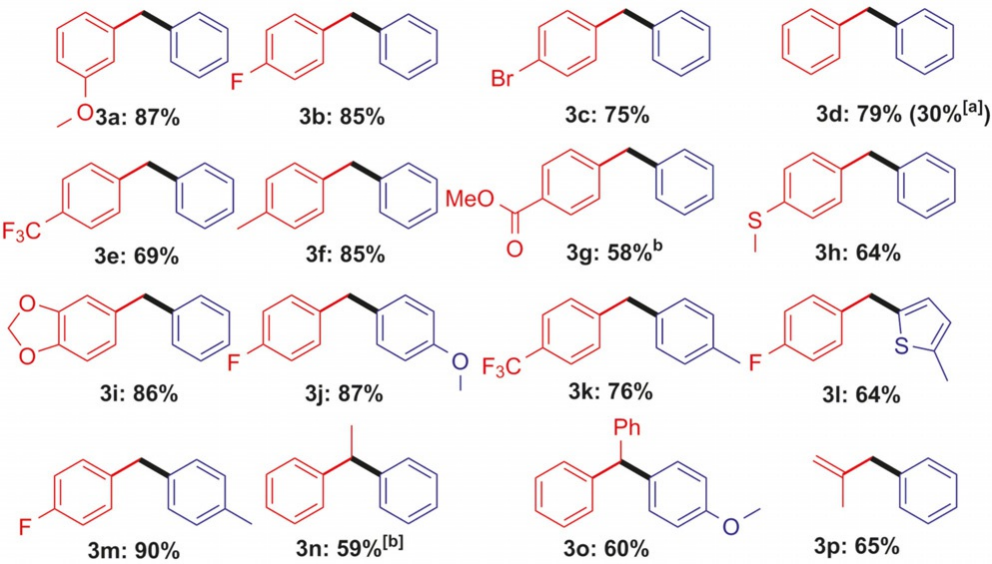

[a] With benzyl chloride. [b] 72 hours.

With a closed‐shell mechanism favoured, and neutral diarylzinc reagents precluded as the active species (since ZnPh_2_ and **2 a** do not cross‐couple in dioxane),^11^ the formation of anionic arylzincates from **1 a** was explored in 2‐MeTHF. Anionic zincates are more nucleophilic than Ar_2_Zn species, and are often more effective in the transfer of aryl groups to electrophiles;^21^ for example, [*t*Bu_2_PhZn]Li cleanly arylates MeI.^22^ To assess for zincate formation, two equivalents of **1 a** were reacted with ZnBr_2_ at 20 °C; in <10 mins *t*BuBPin had completely formed (by NMR spectroscopy) indicating transfer of two equivalents of phenyl to zinc (Scheme 3). The composition of the ensuing zincate species will most likely predominantly be of the form {[Ph_*x*_ZnBr_*y*_]^−^}_*n*_ (*x*+*y=*3, *n*=1 or higher aggregates), although only a single set of ^1^H and ^13^C phenyl resonances were observed, which is consistent with rapid exchange on the NMR timescale (as is the case throughout these experiments).^23^ The zincate assignment is supported by significant changes in the ^1^H and ^13^C{^1^H} NMR spectra on addition of one equivalent of LiBr to ZnPh_2_, whereas a second equivalent of LiBr results in only very minor chemical‐shift changes (indicating minimal formation of [Ph_*x*_ZnBr_*y*_]^2−^ {*x*+*y=*4} species).

**Scheme 3:**
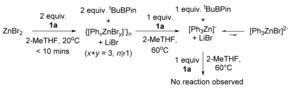
Reaction outcomes between **1 a** and ZnBr_2_.

To determine if an aryl group can be transferred from **1 a** to a diarylzinc species, equimolar ZnPh_2_ and **1 a** were reacted. This resulted in slow (at 20 °C) transfer to form [ZnPh_3_]^−^ (with a diagnostic δ^13^C=168.8 for the *ipso* C_phenyl_ in 2‐MeTHF)^24^ and *t*BuBpin. At 60 °C approximately 2 h were required for formation of [ZnPh_3_]^−^ from ZnPh_2_ and **1 a**. Li[ZnPh_3_], synthesized from ZnPh_2_ and one equivalent of PhLi has a closely comparable δ^13^C_ipso_ for the Zn−Ph moiety (169.5 ppm in 2‐MeTHF). Transmetallation from **1a** to zinc species still proceeds in the presence of LiBr; for example, using a 1:2 mixture of ZnPh_2_/LiBr, 35 % aryl transfer from **1 a** to zinc occurs after 1 h at 60 °C, thus aryl transfer to {[Ph_x_ZnBr_y_]^−^}_*n*_ species does occur. Li[ZnPh_3_] only interacts weakly with 1 equivalent of LiBr (as indicated by very minor changes in the ^1^H and ^13^C NMR resonances, max. Δ*δ*=0.02 ppm; addition of a second equivalent of LiBr results in no observable Δδ). As [Ar_4_Zn]^2−^ are documented_,_ 24, 25 attempts to form [Ph_4_Zn]^2−^ using **1 a** were explored. However, the addition of **1 a** to [Ph_3_Zn]^−^ (made in situ) did not lead to any observable aryl transfer (by NMR spectroscopy) disfavoring formation of [Ph_4_Zn]^2−^ under these conditions. Attempts to crystallise these zincates failed; nevertheless, the above reactions indicate that a major Zn species present during catalysis is [ZnPh_3_]^−^. However, [Ph_*x*_ZnBr_*y*_]^*n*−^ (*x*+*y=*3 or 4, *n*=1 or 2, *y*≥1) will also be present, and will presumably increase in concentration as catalysis proceeds, due to the formation of LiBr as a by‐product from cross‐coupling along with consumption of **1a**. It is notable that combining **1 a** and ZnX_2_ does not produce any observable [Ph_4_Zn]^2−^ in contrast to using PhLi. Therefore using borate **1 a** allows [Ar_3_Zn]^−^ to be selectively accessed without any dianioic zincate formation.^24^ It is also notable that Na[BPh_4_] gives drastically different transmetallation outcomes to **1 a**, since it does not transfer an aryl to ZnPh_2_ in 2‐MeTHF (at 20 °C or 60 °C), and thus does not produce any observable [Ph_3_Zn]^−^. A relative nucleophilicity scale in 2‐MeTHF for these nucleophiles is shown in Scheme 4, with **1 a** uniquely positioned between the monoanionic triaryl and dianionic tetraaryl zincates.

**Scheme 4:**
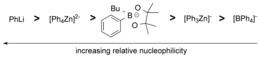
Relative aryl nucleophilicity (in 2‐MeTHF).

The stoichiometric coupling reactivity of various zincates with **2 b** was assessed to determine if any heterocoupling occurs (and the selectivity for heterocoupling). In each case, benzylbromide **2 b** was combined with a zincate mixture containing a specific ratio; for example, ZnPh_*y*_Br_*x*_, generated by combining ZnPh_2_ (or ZnBr_2_) with PhLi (or **1 a**) and LiBr. On combining [ZnPh_4_]^2−^ (formed from PhLi and ZnPh_2_)^24^ and **2 b** in 2‐MeTHF, **2 b** was consumed within 20 min at 25 °C. However, this led exclusively to the homocoupled product **4 b** (Table 3, entry 1). Therefore, for selective heterocoupling, [Ar_4_Zn]^2−^ species have to be avoided, presumably since these are stronger reducing agents, and thus lead to single‐electron‐transfer reactivity. In contrast, [ZnPh_3_]^−^ (made from PhLi and ZnPh_2_) on combination with **2 b** predominantly led to heterocoupling (entry 2). Repeating in the presence of LiBr also led to formation of [ZnPh_3_]^−^ (confirmed by comparable δ^13^C for the *ipso* C_phenyl_, entry 4) and a comparable coupling outcome. Notably, a 1:1 mixture of **1 a**/ZnPh_2_ (post heating at 60 °C for 18 h) when reacted with **2 b** produced predominantly **3 b**, with a slightly improved hetero/homo coupling ratio (entry 3 compared to 2, or 4), suggesting that *t*BuBPin may subtly affect the catalytic process and thus the overall selectivity. A number of mixed zincates, [Ph_*x*_ZnBr_*y*_]^*n*−^ (with δ^13^C resonances supporting the presence of Zn−Br moieties),^26^ were reacted with **2 b**; reactivity was slow at 60 °C, and either more **4 b** was produced than **3 b** (entry 5), or no reaction was observed at all (entry 6). Therefore, the triarylzincates appear to be essential to lead to significant heterocoupling. This is consistent with the increased efficacy of 1.5 equivalents of **1 a** relative to 1.1 equivalents in the catalysis, because otherwise low activity bromide–zincates will dominate as the reaction progresses.

**Table 3:** Zincate reactivity with **2 b**.

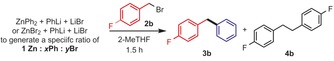						
Entry	*x* Ph	*y* Br	δ^13^C (*ipso*)	T [°C]	**3 b** [%]^[a]^	**4 b** [%]^[a]^
1	4	0	171.4	25^[b]^	0	100
2	3	0	169.5	60	59	11
3^[c]^	3	0	168.8	60	69	9
4	3	2	169.4	60	63	11
5	2	2	160.4	60	3	10
6	1	3	158.7	60	0	0

[a] Yields by ^19^F NMR spectroscopy and GC‐MS, mass balance where appropriate is unreacted **2 b**. [b] 20 min. [c] from ZnPh_2_ and borate **1 a** instead of PhLi.

Previously, zinc Lewis acids were proposed to activate benzylbromides by coordination to bromide, thereby facilitating S_N_2 substitution by arylborates or zincates.^11^ To assess if Lewis acids are present during catalysis, Et_3_PO was added (using the conditions from Table 1 entry 4) after 3 h. The ^31^P{^1^H} NMR spectrum showed a downfield shift of 12.44 ppm compared to free Et_3_PO, confirming that Lewis acidic species are present. However, this may well be due to lithium Lewis acids, since a similar (Δ*δ*=13.98 ppm) downfield shift was observed upon addition of Et_3_PO to LiBr in 2‐MeTHF. Furthermore, a 2:1 mixture of ZnPh_2_/**1 a** was heated in 2‐MeTHF until **1 a** was consumed, targeting a 1:1 mixture of Lewis acidic ZnPh_2_(solvent)_*n*_ and zincate [ZnPh_3_]^−^. To this mixture, **2 b** was added, and the reaction was heated to 60 °C for 1 h, leading to poor coupling selectivity (**3 b**/**4 b** of 2.8:1). Therefore, under these conditions, zinc Lewis acid mediated coupling is disfavoured, and a mechanism involving S_N_2 substitution by a triarylzincate is preferred, possibly involving substrate activation by Li^+^ salts.

In conclusion, benzyl halides can be coupled with aryl borates using ZnBr_2_ as catalyst. To the best of our knowledge this is the first zinc,catalyzed Suzuki–Miyaura reaction. Initial studies indicate an S_N_2 mechanism, with triarylzincates as the key nucleophiles. Our findings represent an advance in the development of less toxic, base‐metal cross‐coupling catalysts as alternatives to established methodologies using noble metals, and further investigations into the detailed mechanism and scope of the reaction are ongoing.

## Conflict of interest

The authors declare no conflict of interest.

## Supporting information

As a service to our authors and readers, this journal provides supporting information supplied by the authors. Such materials are peer reviewed and may be re‐organized for online delivery, but are not copy‐edited or typeset. Technical support issues arising from supporting information (other than missing files) should be addressed to the authors.

Supplementary
