# Supplementary material for: A Zinc Catalyzed C(sp3)−C(sp2) Suzuki–Miyaura Cross‐Coupling Reaction Mediated by Aryl‐Zincates
Source: Chemistry. 2017 Oct 19;23(63):15889–93. doi: 10.1002/chem.201704170 (PMC5915750; doi:10.1002/chem.201704170)
Supplement: Supplementary file 1 — Supplementary [file CHEM-23-15889-s001.pdf]

CHEMISTRY
A European Journal

Supporting Information

A Zinc Catalyzed C(sp^3)–C(sp^2) Suzuki–Miyaura Cross-Coupling Reaction Mediated by Aryl-Zincates

Richard J. Procter,[a] Jay J. Dunsford,[a] Philip J. Rushworth,[b] David G. Hulcoop,[b] Richard A. Layfield,[a] and Michael J. Ingleson*[a]

chem_201704170_sm_miscellaneous_information.pdf

Contents

General Remarks 2
Synthesis of borate nucleophiles 3
Alkoxide activated borate – transmetallation with $ZnBr_2$ 7
Borate-to-$ZnBr_2$ Transmetallation in CPME and Cross-Coupling in benzene............ 9
Preliminary catalysis investigations 10
Solvent optimisation reactions............ 11
Trace metal control reactions............ 12
Nucleophile optimisation reactions 16
Substrate scope screening reactions and experimental data 18
Radical inhibition studies 38
Zincate reactivity studies 39
Triethylphosphine oxide Lewis acidity test............ 58
NMR Spectra of new compounds............ 59
References............ 66

General Remarks

Unless otherwise stated all manipulations were carried out using standard Schlenk techniques under argon or in an MBraun UniLab glovebox, under an atmosphere of argon. THF, 2MeTHF, dioxane and cyclopentyl methyl ether (CPME) were dried over and distilled from potassium and stored over activated 3 Å molecular sieves. Hexane was dried and distilled from either calcium hydride or NaK alloy and stored over a potassium mirror. All other reagents were purchased from commercial chemical suppliers and used as received. NMR spectra were recorded on Bruker AvanceIII-400, Bruker AvanceII-500 or Bruker Ascend-400 spectrometers. Chemical shifts are reported as dimensionless values and are frequency referenced relative to residual protio- impurities in the NMR solvents for ^{1}H and $^{13}C\{^{1}H\}$ respectively, while $^{11}B\{^{1}H\}$, $^{19}F\{^{1}H\}$, ^{7}Li and ^{31}P shifts are referenced relative to external BF_3-etherate, hexafluorobenzene, LiCl, and H_3PO_4 respectively. Coupling constants J are given in Hertz (Hz) as positive values regardless of their real individual signs. The multiplicity of the signals are indicated as "s", "d", or "q" for singlet, doublet, or quartet respectively. GC-MS analysis was performed on either of two instruments. An Agilent Technologies 7890A GC system equipped with an Agilent Technologies 5975C inert XL EI/CI MSD with triple axis detector, fitted with a HP-5Ms column, with dimensions 30 m length; 0.250 mm internal diameter; and 0.25 μm film. Or an Agilent Technologies 6890N GC equipped with an Agilent Technologies 5973N EI MSD, fitted with a HP-5MS column, with dimensions 30 m length; 0.250 mm internal diameter; and 0.25 μm film.

The relative response factors for GCMS analysis of the heterocoupled and homocoupled products derived from the fluorinated electrophile, 2b, were calculated using values from $^{19}F\{^{1}H\}$ NMR spectra (with a delay time of 35s to allow full spin-lattice relaxation) where their integrals could be measured accurately. When these resonances were overlapped in the ^{19}F $\{^{1}H\}$ NMR spectra (which occurred in a number of solvents), GCMS analysis was used to calculate their ratio, and yields calculated by using the overall integral of the overlapped peak with this ratio applied (accounting for the 2 equivalents of electrophile involved in the homocoupled product). The relative response factors used in this calculation for GCMS analysis were calculated from the results where ^{19}F resonances could be accurately integrated. Yields are based on the electrophile as the limiting reagent and for homocoupling impurities the 19F integrals are scaled by 0.5 to give a molar ratio vs. heterocoupling (i.e. A 1:1 hetero:homocoupled product ratio at full conversion, would be reported as 33 % : 33 %, due to the additional molecule of starting material required in the production of the homocoupled product). In a number of cases the ^{13}C resonance for the carbon atom directly bonded to boron was not observed due to the effect of quadrupolar relaxation.

Synthesis of borate nucleophiles

General Procedure

The borates were synthesised according to a modified literature procedure[1]. In an oven dried Schlenk flask the appropriate arylboronic acid pinacol ester (1-1.05 eq.) was dissolved in anhydrous hexane and cooled to -78 °C before dropwise addition of *tert*-butyllithium (1.7M in pentane, 1 eq.). The reaction was allowed to warm to room temperature and stirred overnight at room temperature, over which period a precipitate formed. The borate was isolated by filtration, washed with anhydrous hexane and residual solvent removed under reduced pressure.

[Li][(^{t}Bu)(Ph)B(Pin)] (1a)

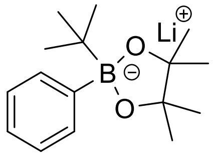

Synthesised according to the above general procedure, from phenylboronic acid pinacol ester (3 g, 14.7 mmol), and ^{t}BuLi (1.7M/pentane, 8.5 ml, 14.5 mmol). Isolated as a free flowing white powder. Yield: 3.6g, 13.4 mmol, 92%. NMR spectroscopic data match previously reported values[2].

[Li][(^{t}Bu)(*p*-tol)B(Pin)] (1b)

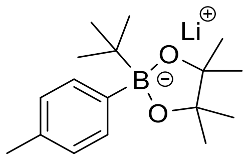

Synthesised according to the above general procedure, from *p*-tolylboronic acid pinacol ester (1.136g, 5.21 mmol) and ^{t}BuLi (1.7M/pentane, 3ml, 5.1 mmol). Isolated as a free flowing white powder. Yield: 1.35g, 4.8 mmol, 94%.

^{1}H NMR (d_8-THF, 400 MHz) : δ 7.25 (d, *J*=7.53 Hz, 2 H), 6.79 (d, *J*=7.78 Hz, 2 H), 2.19 (s, 3 H), 1.13 (s, 6 H), 0.84 (s, 6 H), 0.61 ppm (s, 9 H)

^{11}B{^{1}H} NMR (d_8-THF, 128 MHz) : δ 8.15 ppm

^{7}Li{^{1}H} NMR (d_8-THF, 155 MHz): δ 0.15 ppm

^{13}C{^{1}H} NMR (d_8-THF, 101 MHz): δ 133.32, 131.53, 126.88, 78.49, 30.93, 28.62, 28.14, 21.60 ppm

[Li][(^{t}Bu)(*p*-MeO-C_6H_4-)B(Pin)] (1c)

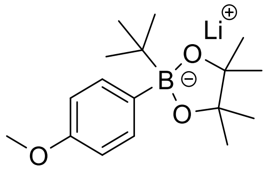

Synthesised according to the above general procedure, from *para*-methoxyphenylboronic acid pinacol ester (650 µl, 3.22 mmol) and ^{t}BuLi (1.7M/pentane, 1.9 ml, 3.23 mmol). Isolated as a free flowing white powder. Yield: 877 mg, 2.94 mmol, 91%.

^{1}H NMR (d_8-THF, 400 MHz) : δ 7.26 (d, *J*=8.03 Hz, 2 H), 6.57 (d, *J*=8.28 Hz, 2 H), 3.66 (s, 3 H), 1.13 (s, 6 H), 0.84 (s, 6 H), 0.62 ppm (s, 9 H)

^{11}B{^{1}H} NMR (d_8-THF, 128 MHz) : δ 8.11 ppm

^{7}Li{^{1}H} NMR (d_8-THF, 155 MHz): δ 0.16 ppm

^{13}C{^{1}H} NMR (d_8-THF, 101 MHz): δ 157.48, 133.88, 111.80, 78.54, 54.94, 30.94, 28.69, 28.18 ppm

[Li][(^{t}Bu)(*p*-F_3CO-C_6H_4-)B(Pin)] (1d)

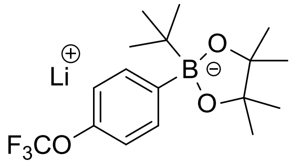

Synthesised according to the above general procedure, from 4-(trifluoromethoxy)phenyl boronic acid pinacol ester (490 mg, 1.7 mmol) and ^{t}BuLi (1.7M/pentane, 1 ml, 1.7 mmol). Isolated as a free flowing off-white powder. Yield: 359 mg, 1.02 mmol, 60%.

^{1}H NMR (d_8-THF, 400 MHz) : δ 7.46 (d, *J*=8.3 Hz, 2 H), 6.81 (d, *J*=7.5 Hz, 2 H), 1.11 (s, 6 H), 0.78 (s, 6 H), 0.60 ppm (s, 9 H)

^{11}B{^{1}H} NMR (d_8-THF, 128 MHz) : δ 7.6 ppm

^{7}Li{^{1}H} NMR (d_8-THF, 155 MHz): δ 0.02 ppm

^{19}F{^{1}H} NMR (d_8-THF, 376 MHz): δ -58.2 ppm (s)

^{13}C{^{1}H} NMR (d_8-THF, 101 MHz): δ 146.8, 134.6, 122.1 (q, *J*=253 Hz), 117.7, 78.6, 30.7, 28.7, 28.3 ppm

[Li][(^{t}Bu)(Me-C_4H_2S)B(Pin)] (1e)

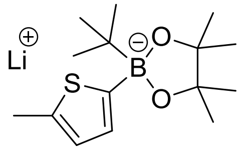

1e was isolated with a by-product that is tentatively assigned as [Li][(Me-C_4H_2S)$_2$B(Pin)] that accounts for approximately 13% of the overall amount, assignment is based on comparison to literature reports.[3]

^{1}H NMR (d_8-THF, 400MHz) : 6.53 (d, J=3Hz 1 H) 6.49 (m, 1 H) 2.36 (s, 4 H) 1.11 (s, 10 H) 0.98 (s, 6 H) 0.66 ppm (s, 9 H)

^{11}B{^{1}H} NMR (d_8-THF, 128 MHz) : δ 7.5 ppm

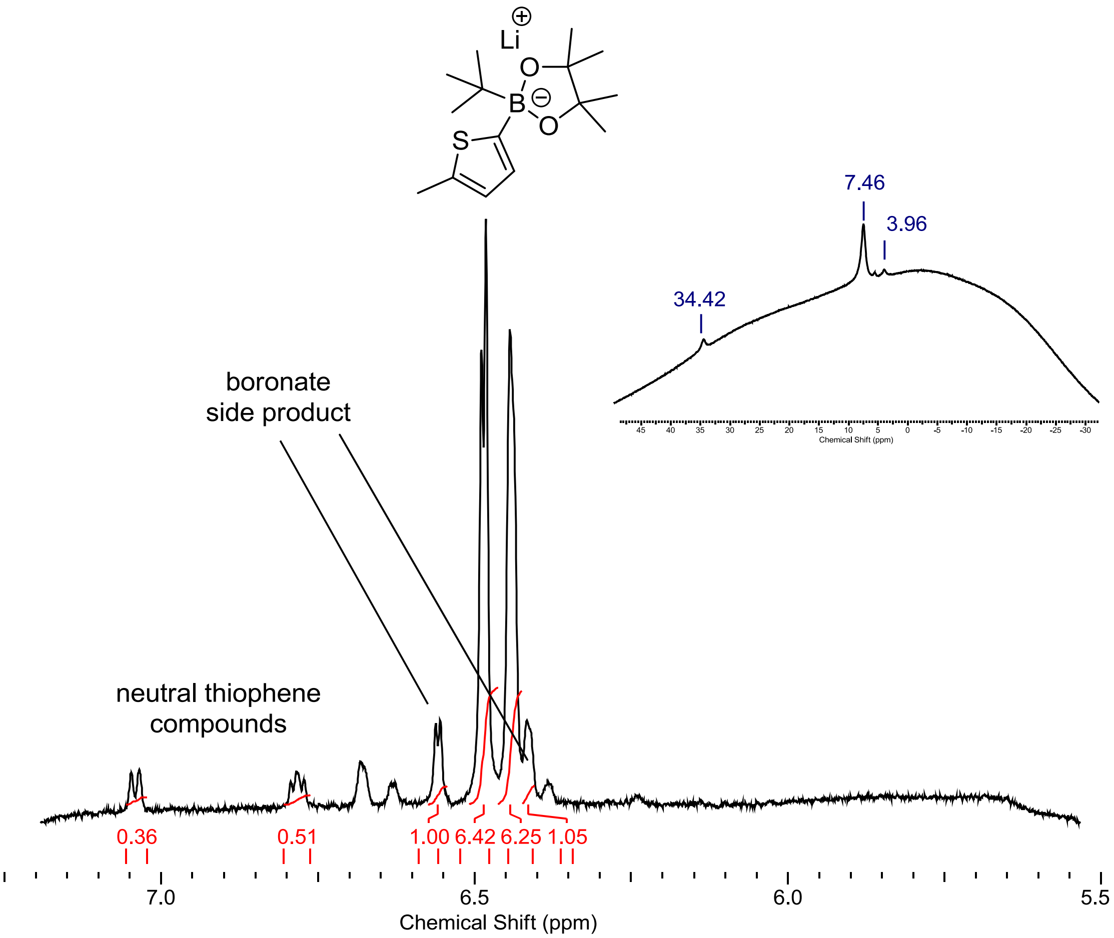

Figure S 1: ^{1}H NMR spectrum ($^{1}H_8$- THF) of the aryl region of 1e. Inset: ^{11}B NMR spectrum

Synthesis of [Li][(^{n}Bu)(Ph)B(Pin)] (5)

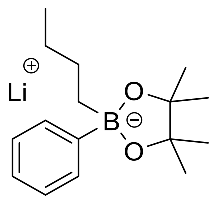

Synthesised according to the above general procedure, from phenyl boronic acid pinacol ester (650 mg, 3.2 mmol) and ^{n}BuLi (1.6M/hexane, 2 ml, 3.2 mmol). Isolated as a free flowing off-white powder. Yield: 678 mg, 79%. NMR spectroscopic data is consistent with previously reported values.[1]

Synthesis of [Li][(O^{t}Bu)(Ph)B(Pin)] (6)

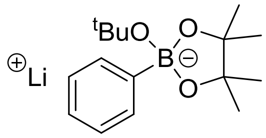

An oven dried Schlenk tube was charged with phenyl boronic acid pinacol ester (500 mg, 2.5 mmol) and anhydrous hexane (9 ml) and a solution of lithium *tert*-butoxide (200 mg, 2.5 mmol) in hexane (10 ml) was added slowly. The homogenous mixture was stirred and of 2.5 ml THF was added, before stirring for a further 4 hrs. During this time, a white precipitate had formed, which was isolated by filtration and washed with anhydrous hexane (2 x 4ml). Residual solvent was removed under reduced pressure, giving a free flowing white powder in 258 mg. The solvent components were combined and stirred overnight leading to more precipitation which was isolated above to give a second crop of 133mgs. Combined yield of both crops = 56 %.

^{1}H NMR (*protio*-THF, 400 MHz) : δ 7.48 (d, *J*=7.03 Hz, 2 H) 6.93 (t, *J*=7.28 Hz, 2 H) 6.83 (t, *J*=7.03Hz, 1 H) 1.10 (s, 6 H) 0.97 (s, 9 H) 0.88 ppm (s, 6 H)

^{11}B{^{1}H} NMR (d_8-THF, 128 MHz) : δ 6.7 ppm

Alkoxide activated borate – transmetallation with $ZnBr_2$

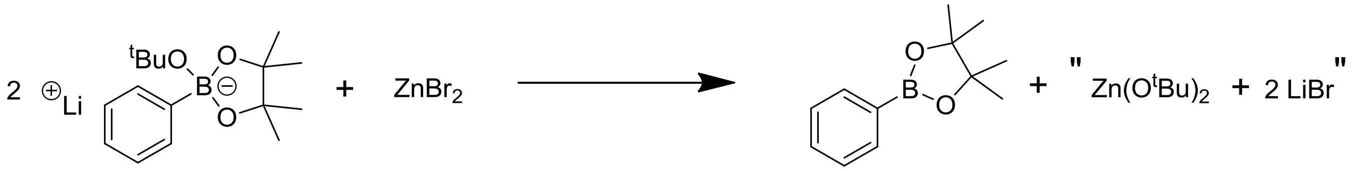

In an oven dried J Young's NMR tube, [Li][(O^{t}Bu)(Ph)B(Pin)] (20 mg, 0.07 mmol), and $ZnBr_2$ (8 mg, 0.035 mmol) were dissolved in anhydrous THF, with a DMSO-d_6 capillary. After 2 hours the mixture was analysed by ^{11}B NMR spectroscopy, revealing loss of the starting material resonance at (6.7 ppm) and formation of the neutral PhBPin (30.6 ppm), indicating preferential transfer of the ^{t}BuO$^-$group to the zinc bromide.

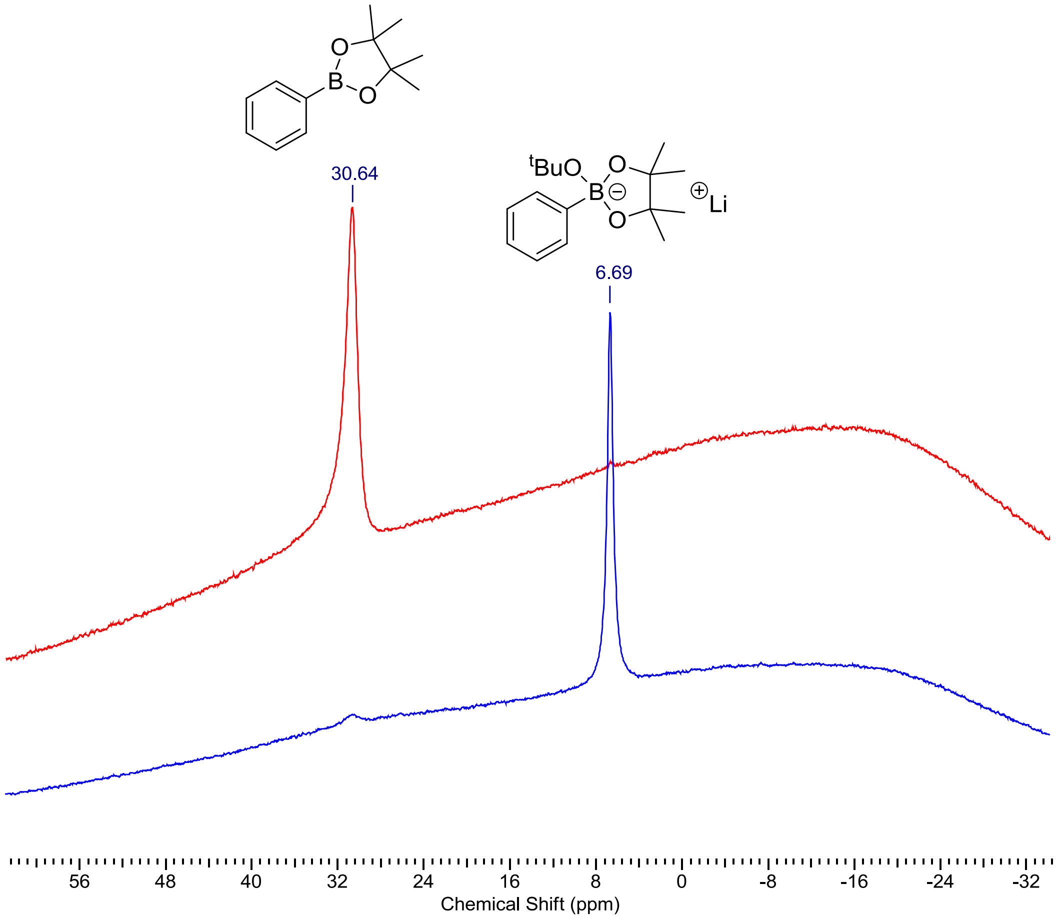

Figure S 2: ^{11}B NMR spectrum of 6 before (bottom) and after (top) addition of $ZnBr_2$

Furthermore, attempts to use alkoxide borate **6** (1.5 eq.) /$ZnBr_2$ (10 mol%) to couple with **2b** led to no heterocoupling (18 h at 60°C in 2-MeTHF).

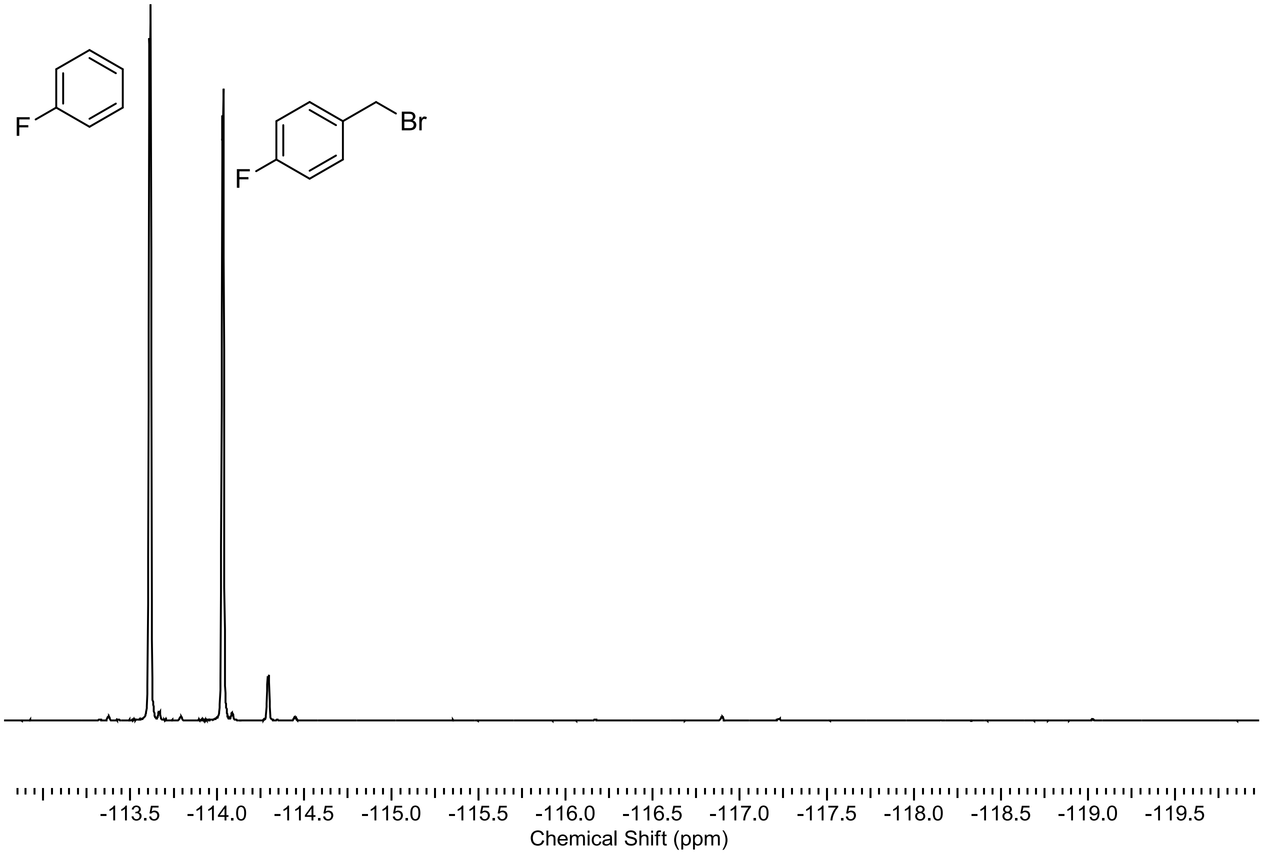

Figure S 3 : ^{19}F NMR spectrum of the attempted coupling between 6 and 2b using $ZnBr_2$ catalysis (fluorobenzene added as a NMR standard)

Borate-to-$ZnBr_2$ Transmetallation in CPME and Cross-Coupling in benzene

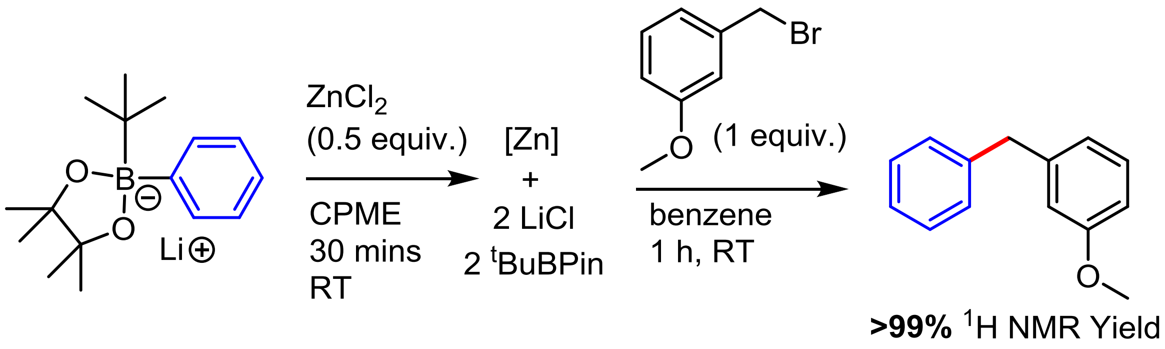

A J. Youngs ampoule equipped was loaded with [PhBPin(^{t}Bu)][Li] (268.1 mg, 1.0 mmol) and anhydrous $ZnCl_2$ (68.2 mg, 0.5 mmol) prior to the addition of anhydrous CPME (2.0 mL). The reaction mixture was stirred at ambient temperature for 30 minutes prior to the removal of all volatiles to afford an oily residue. The residue was taken up in anhydrous benzene (2.0 mL) prior to the addition of 3-methoxybenzyl bromide (70 µL, 0.5 mmol) and the reaction mixture stirred for 1 h at ambient temperature which led to the deposition of a colourless solid. Filtration of the reaction mixture and analysis by ^{1}H NMR spectroscopy demonstrated the conversion to the desired diarylmethane C*sp*^{2}-C*sp*^{3} cross-coupled product (*Figure S4*).

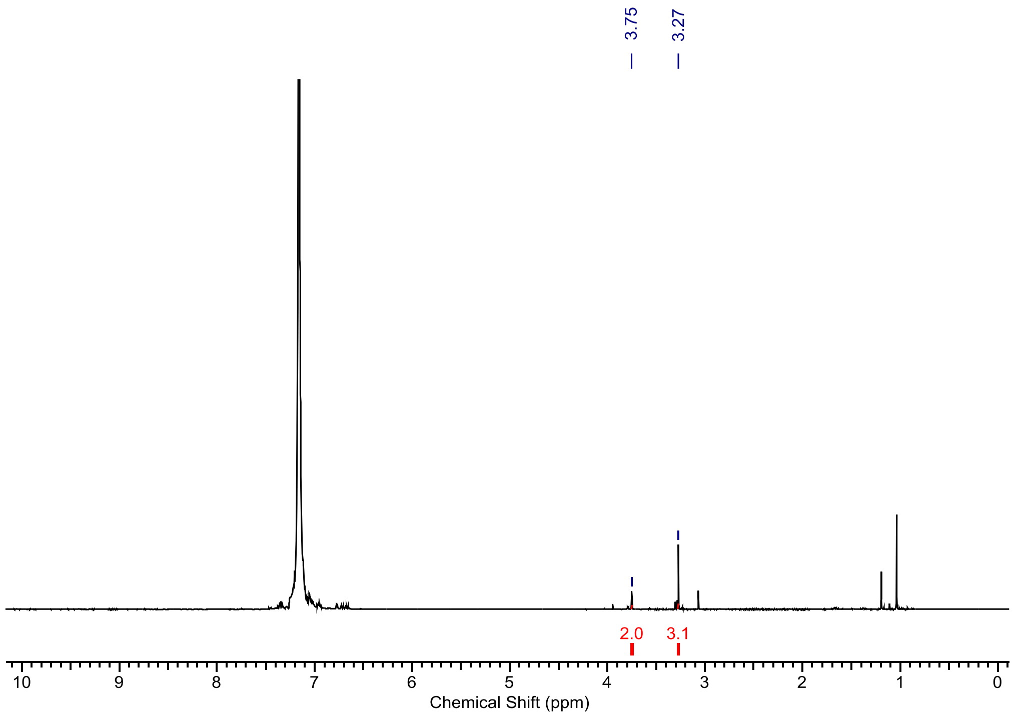

Figure S 4: Crude ^{1}H NMR spectrum (C_6H_6/C_6D_6, 400 MHz, 298K) of the reaction mixture after 1 h at ambient temperature post the addition of 3-methoxybenzylbromide showing complete consumption of benzyl bromide and formation of the heterocoupling product 3a.

Preliminary catalysis investigations

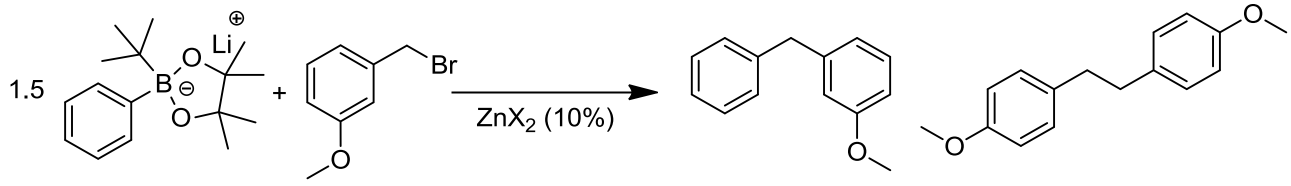

In an oven dried J Young's ampoule [Li][(^{t}Bu)(Ph)B(Pin)] **1a** (94 mg, 0.35 mmol) and $ZnPh_2$(5 mg, 0.02 mmol) and 3-methoxybenzyl bromide (32 µl, 0.23 mmol) were combined and dissolved in the appropriate solvent (2 ml). The reaction was heated to the desired temperature for 17 hours prior to addition of mesitylene (32 µl, 0.23 mmol) as an internal standard and transfer to an NMR tube under ambient conditions. Subsequently the mixture was diluted with DCM, filtered through a silica plug and analysed by GCMS.

Table S 1: Results from GCMS analysis

**Solvent, Temperature**	**Catalyst**	**2a**	**3a**	**4a**	**Biphenyl**
CPME, 100°C	$ZnPh_2$	0	1	0.61	0.53
CPME, 60°C	$ZnPh_2$	0.92	1	0.65	0.57
CPME, 100°C	$ZnCl_2$	0	1	0.57	0.47
Dioxane, 60°C	$ZnPh_2$	0.12	1	0.03	0.02

(GCMS integration as ratios vs **3a**. Not calibrated for response factors)

Solvent optimisation reactions

General Procedure

In an oven dried J Young's ampoule [Li][(^{t}Bu)(Ph)B(Pin)] **1a** (94 mg, 0.35 mmol) and $ZnBr_2$ (5 mg, 0.02 mmol) were dissolved in the appropriate solvent (2 ml). Then 4-fluorobenzyl bromide (29 µl, 0.23 mmol) was added. The reaction was heated to the appropriate temperature for 18 hours before quenching with ethanol followed by addition of fluorobenzene (22 µl, 0.23 mmol) and mesitylene (32 µl, 0.23 mmol) as internal standards. The mixture was directly analysed by ^{19}F NMR spectroscopy before dilution with DCM, filtration through a silica plug and analysis by GCMS.

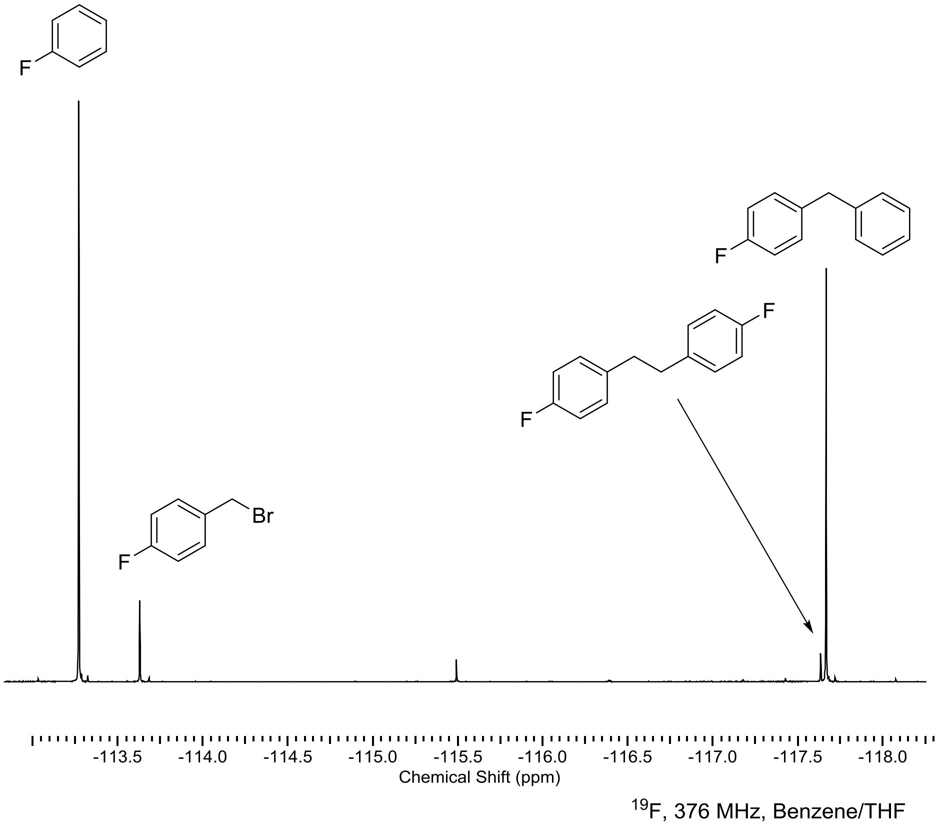

Figure S 5: Representative ^{19}F{^{1}H} NMR spectrum (from reaction using a 10:1 benzene / THF mixture)

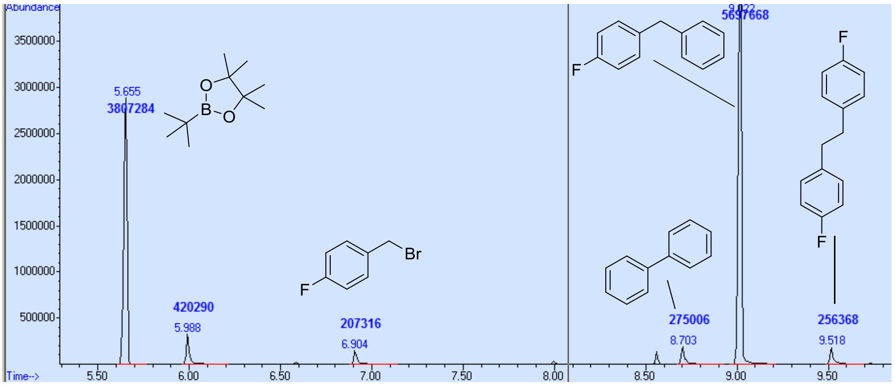

Figure S 6: Representative GCMS trace (from reaction using a 10:1 benzene / THF mixture)

Trace metal control reactions

General Procedure

In an oven dried J Young's ampoule [Li][(^{t}Bu)(Ph)B(Pin)] **1a** (94 mg, 0.35 mmol) and the appropriate metal salt were dissolved in 2-MeTHF (2ml). 4-fluorobenzyl bromide (29 µl, 0.23 mmol) was added and the mixture heated to 60°C for 18 hours before quenching with ethanol or dilute HCl followed by addition of fluorobenzene (22 µl, 0.23 mmol) and mesitylene (32 µl, 0.23 mmol) as standards for analysis. When possible the mixture was directly analysed by $^{19}F\{^1H\}$ NMR spectroscopy before dilution with DCM, filtration through a silica plug and analysis by GCMS.

Without added catalyst:

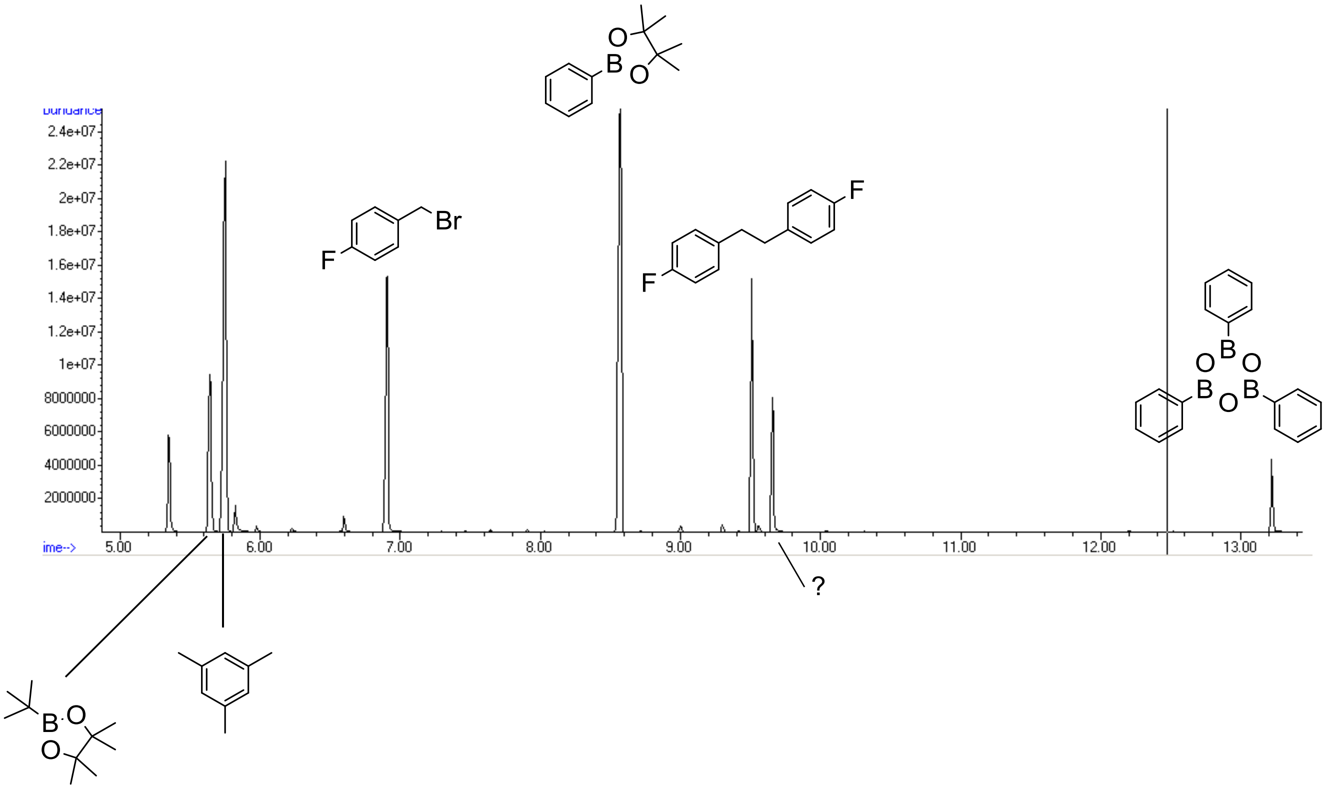

Figure S 7: GCMS chromatogram of the reaction without added catalyst (table 1 entry 6)

The fraction at 9.65 min retention times has a m/z of 202.1 thus is not heterocoupling or biphenyl. Currently it is an unidentified by-product from the reaction

Competition reaction Benzyl vs. Aryl bromide with palladium or zinc

In an oven dried J Young's ampoule [Li][(^{t}Bu)(Ph)B(Pin)] **1a** (135 mg, 0.5 mmol 2.1 eq.) and either Pd(PPh_3)$_4$ (8 mg, 3 mol%) *or* $ZnBr_2$ (5 mg, 10 mol%) and 4-bromobenzyl bromide (60 mg, 0.024 mmol) were added and then dissolved in 2-MeTHF (2 ml). The mixture was then heated to 60 °C for 24 hours, before quenching with dilute HCl (for Pd) or ethanol (for Zn), extraction into or dilution with DCM, filtration through a plug of silica and analysis by GCMS.

Scheme S 1: Aryl-bromide vs benzyl bromide competition control reactions

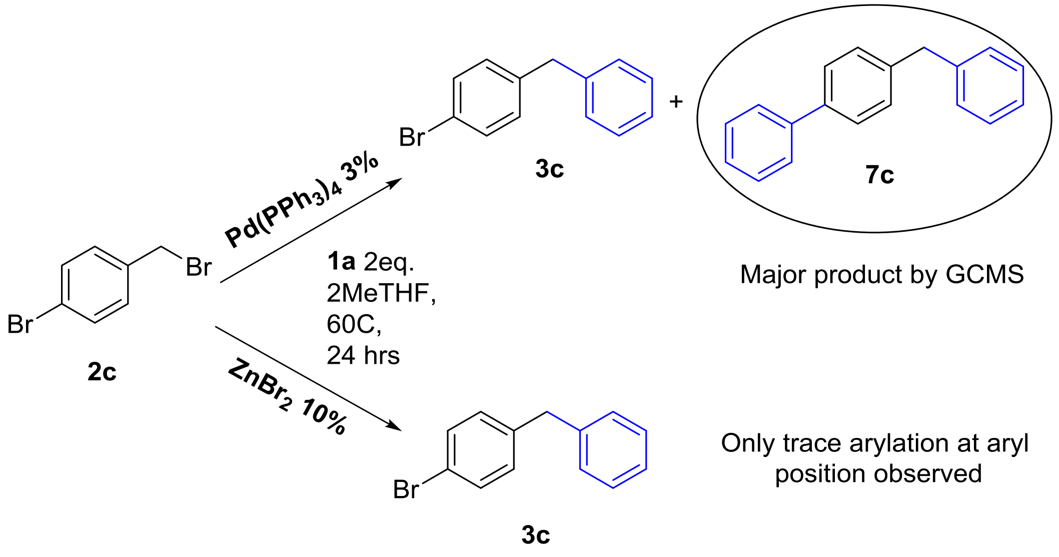

Table S 2: Competition reactions - area in GC chromatogram relative to mono-arylated product

Catalyst	2c	3c	7c
$ZnBr_2$	0	1	0.003
Pd(PPh_3)$_4$	0	1	1.41

Kumada coupling with $ZnBr_2$ and with $FeBr_2$

$ZnBr_2$ (5mg, 10 mol%) was added to an ampoule and dissolved in 1.5 ml 2-MeTHF. 4-fluorobenzylbromide was then added (29µl, 0.233 mmol, 1 equiv). PhMgBr (480µl, 0.725 M solution in 2-MeTHF, 0.35 mmol 1.5 eq.) was added slowly and the reaction was heated at 60°C for 18 h. The mixture was quenched by addition of 0.2 ml EtOH followed by addition of fluorobenzene (22 µl, 0.23 mmol) and mesitylene (32 µl, 0.23 mmol) as internal standards. The mixture was directly analysed by $^{19}F\{^{1}H\}$ NMR spectroscopy before dilution with DCM, filtration through a silica plug and analysis by GCMS.

An identical procedure was used replacing $ZnBr_2$ with $FeBr_2$.

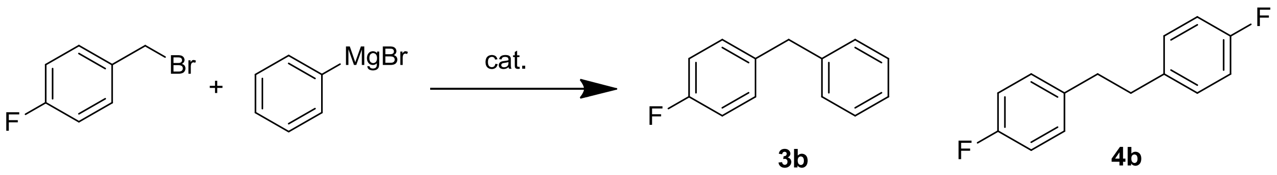

Table S 3: Kumada coupling results[a]

Catalyst	3b	4b	Biphenyl	Ratio 3b:4b[b]
$ZnBr_2$ (10%)	0.09	0.75	1.03	0.13
$FeBr_2$ (10%)	0.70	0.66	0.98	1.19

[a]GCMS integration vs. an internal mesitylene standard. [b]Ratio with adjustment for relative response factors calculated from previous results using ^{19}F NMR spectroscopy.

Attempted synthesis of cycloheptyl benzene

In an oven dried ampoule **1a** (141 mg 0.525 mmol, 1.5eq) was dissolved in 2.25ml 2MeTHF. To this was added 750 µl of a 0.047M stock solution of $ZnBr_2$ in 2MeTHF (0.035mmol, 0.1 eq.), immediately followed by cycloheptyl bromide (48 µl, 0.35 mmol, 1 eq.). The reaction was heated at 60 °C for 24 hours before quenching with EtOH (~2ml), followed by extraction into DCM (3 x ~10 ml) and removal of solvent under reduced pressure. An aliquot was then taken for analysis by GC-MS. The desired product was produced in only trace quantities, with the major product being cycloheptene. This is in contrast to work by Bedford *et al* using iron catalysts which efficiently couple cycloheptyl bromide with **1a**[4].

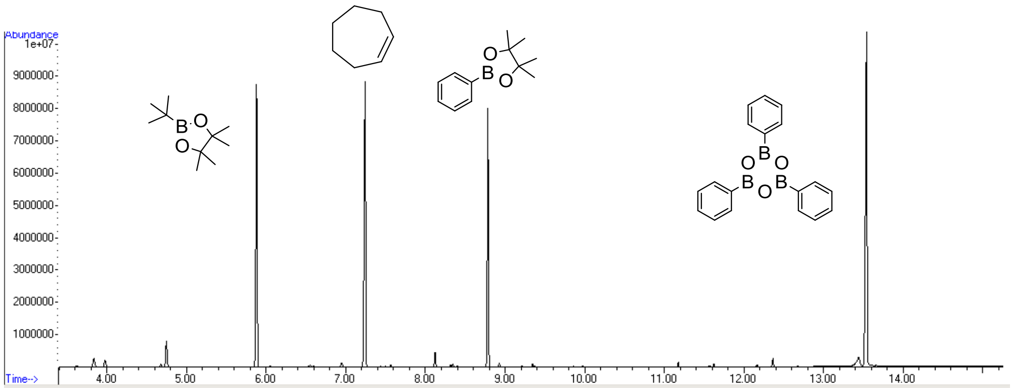

Figure S 8: GCMS Chromatogram of the attempted coupling of cycloheptyl bromide with 1a

An analogous reaction was run using octylbromide in place of cyclohepthylbromide under otherwise identical conditions. Analysis by GC-Ms again showed minimal coupling with the major species being the starting electrophile along with triphenylboroxine.

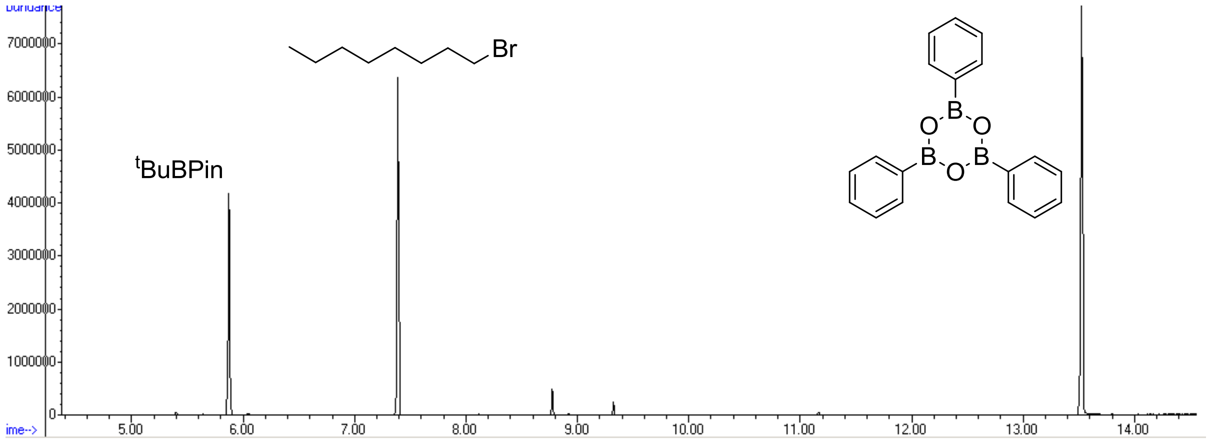

Figure S 9: GCMS Chromatogram of the attempted coupling of octyl bromide with 1a

Nucleophile optimisation reactions

General Procedure

In an oven dried J Young's ampoule [Li][($^{n\ or\ t}$Bu)(Ph)B(Pin)] (94 mg, 0.35 mmol) and zinc bromide (5mg, 0.02 mmol) were dissolved in 2-MeTHF (2ml). 4-fluorobenzyl bromide (29 µl, 0.23 mmol) was added and the mixture heated to 60°C for 18 hours before quenching with ethanol followed by addition of fluorobenzene (22 µl, 0.23 mmol) and mesitylene (32 µl, 0.23 mmol) as internal standards. The mixture was directly analysed by $^{19}F\{^{1}H\}$ NMR spectroscopy before dilution with DCM, filtration through a silica plug and analysis by GCMS.

Procedure for reaction using $NaBPh_4$

In an oven dried ampoule $NaBPh_4$ (180 mg, 0.525 mmol, 1.5eq) was dissolved/suspended in 2.25ml 2MeTHF. To this 750 µl of a 0.047M stock solution of $ZnBr_2$ in 2MeTHF (0.035mmol, 0.1 eq) was added, immediately followed by 4-fluorobenzyl bromide (44 µl, 0.35 mmol). The reaction was heated at 60 °C for 24 hours before quenching with ethanol (~0.2ml), followed by extraction into DCM (3 x ~10 ml) and removal of solvent under reduced pressure. The solid was dissolved in $CDCl_3$ followed by addition of fluorobenzene (22 µl, 0.23 mmol) and mesitylene (32 µl, 0.23 mmol) as internal standards. The mixture was directly analysed by $^{19}F\{^{1}H\}$ NMR spectroscopy and analysis by GCMS.

Table S 4: Nucleophile optimization

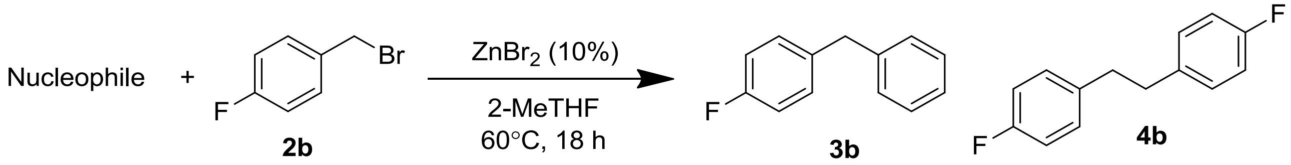

Nucleophile	Equivalents vs. **2b**	**3b** / % yield	**4b** / % yield
**1a** 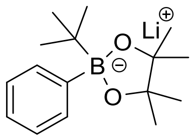	1.5	90	1
**1a**[a]	1.5	71	1
**1a**	1.1	59	4
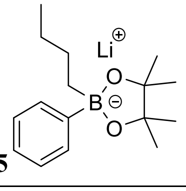 **5**	1.5	47	trace
**5**[b]	1.5	59	10
$NaBPh_4$[b]	1.5	19[c]	2

[a]Borate generated *in situ* (at -78°C for 20 minutes then warmed to RT and held for 1 h). [b]24 hours, 80 °C [c]This is an overestimate as the resonance in the NMR spectrum is overlapped with impurities.

Attempted coupling using ArylBPin and $ZnEt_2$

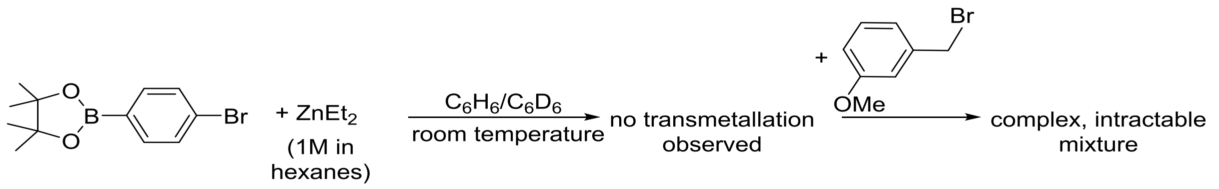

A J. Youngs NMR tube was loaded with 4-Br-C_6H_4-BPin (84.9 mg, 0.3 mmol) and dissolved in a 0.5 ml mixture of C_6D_6/C_6H_6 and then $ZnEt_2$ was added (0.3 mL of a 1 M hexanes solution) to furnish a colourless homogenous reaction mixture. After rotating for 30 minutes multinuclear NMR spectroscopy revealed that minimal transmetallation had occurred (30 minutes was chosen to be comparable with the transmetallation with borate **1a** on page S9). At this stage one equivalent of the electrophile, 3-methoxybenzylbromide) was added (0.3 mmol, 42 μL) which resulted in no observable change (visibly or by ^{11}B NMR spectroscopy) even after 1 h at 20°C. Subsequent heating overnight led to minimal Csp^2-Csp^3 coupling (as indicated by the resonance at 30.3 ppm in the ^{11}B NMR spectrum dominating which is consistent with the starting arylBPin reagent). Benzene was chosen as reaction solvent in this case to maximise the transmetallation and subsequent coupling (coordinating solvents would hinder both steps by binding to the zinc Lewis acids).

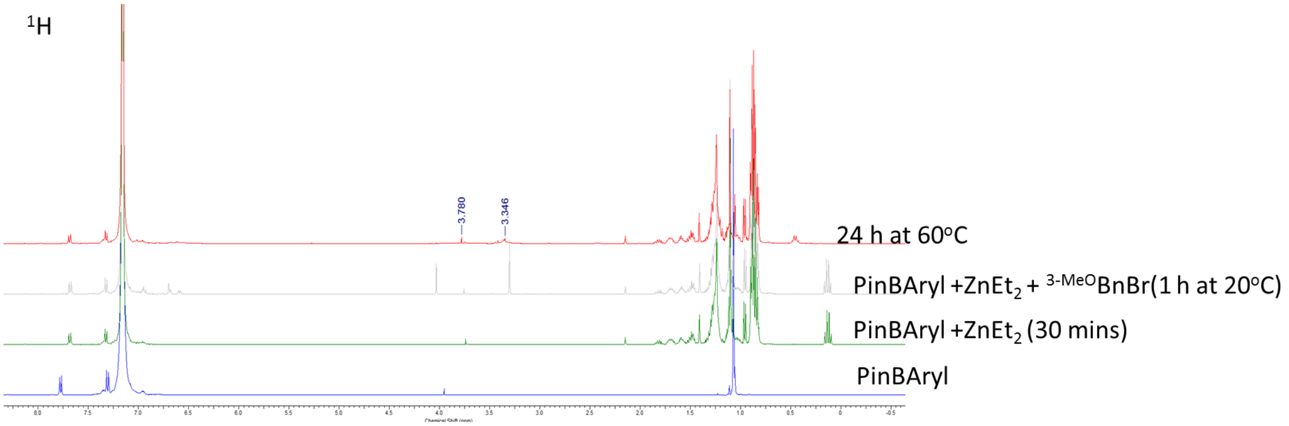

Fig S10: ^{1}H NMR spectrum of attempted cross coupling using AryBpin/$ZnEt_2$

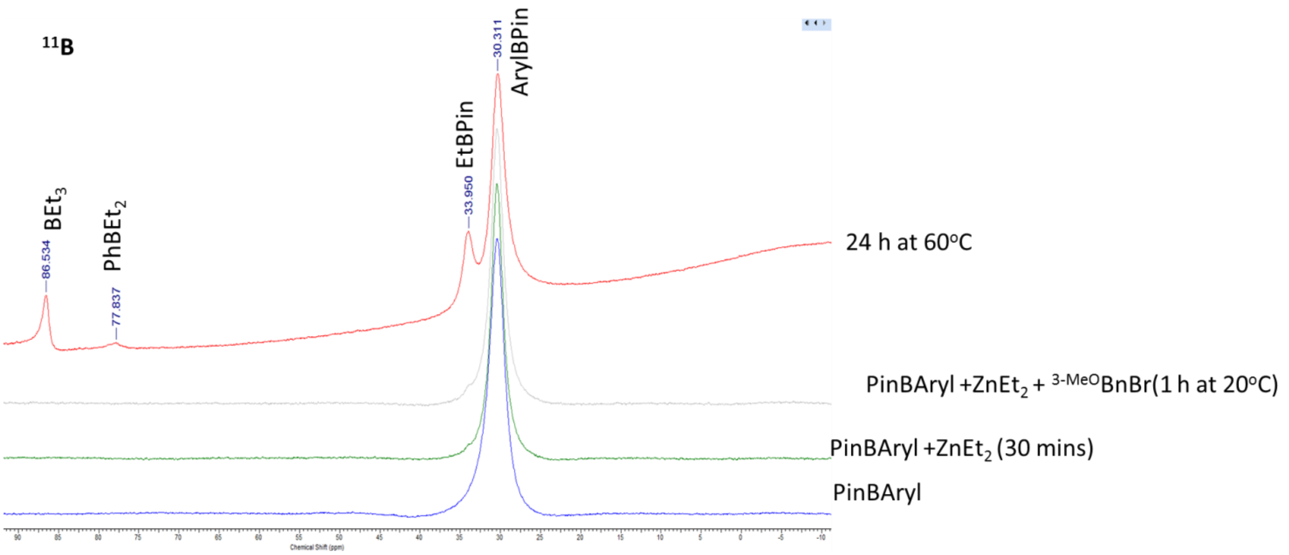

Fig S11: ^{11}B{^{1}H} NMR spectrum of attempted cross coupling using ArylBpin/$ZnEt_2$.

Substrate scope screening reactions and experimental data

General Procedure

In an oven dried ampoule the appropriate borate salt (0.525 mmol, 1.5eq) was dissolved in 2.25 ml 2MeTHF. To this was added 750 µl of a 0.047M stock solution of $ZnBr_2$ in 2MeTHF (0.035 mmol, 0.1 eq), immediately followed by the alkyl halide (0.35 mmol, 1 eq.). The reaction was heated at 60 °C for 24 hours before quenching with 1M aqueous HCl (~2ml), followed by extraction into DCM (3 x ~10 ml) and removal of solvent under reduced pressure. Triphenylmethane (85.5 mg, 0.35 mmol, 1 eq.) or mesitylene was added as an internal standard for NMR yield calculations and the mixture was dissolved in $CDCl_3$ and analysed by NMR spectroscopy. An aliquot was then taken for analysis by GC-MS.

1-benzyl 3-methoxybenzene (3a)

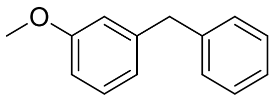

Synthesised according to the above general procedure from 3-methoxybenzyl bromide (49 µl) and [Li][(^{t}Bu)(Ph)B(Pin)] (141 mg). ^{1}H NMR spectroscopic data is consistent with previously reported values[5]. Yield 87% using triphenylmethane as an internal standard.

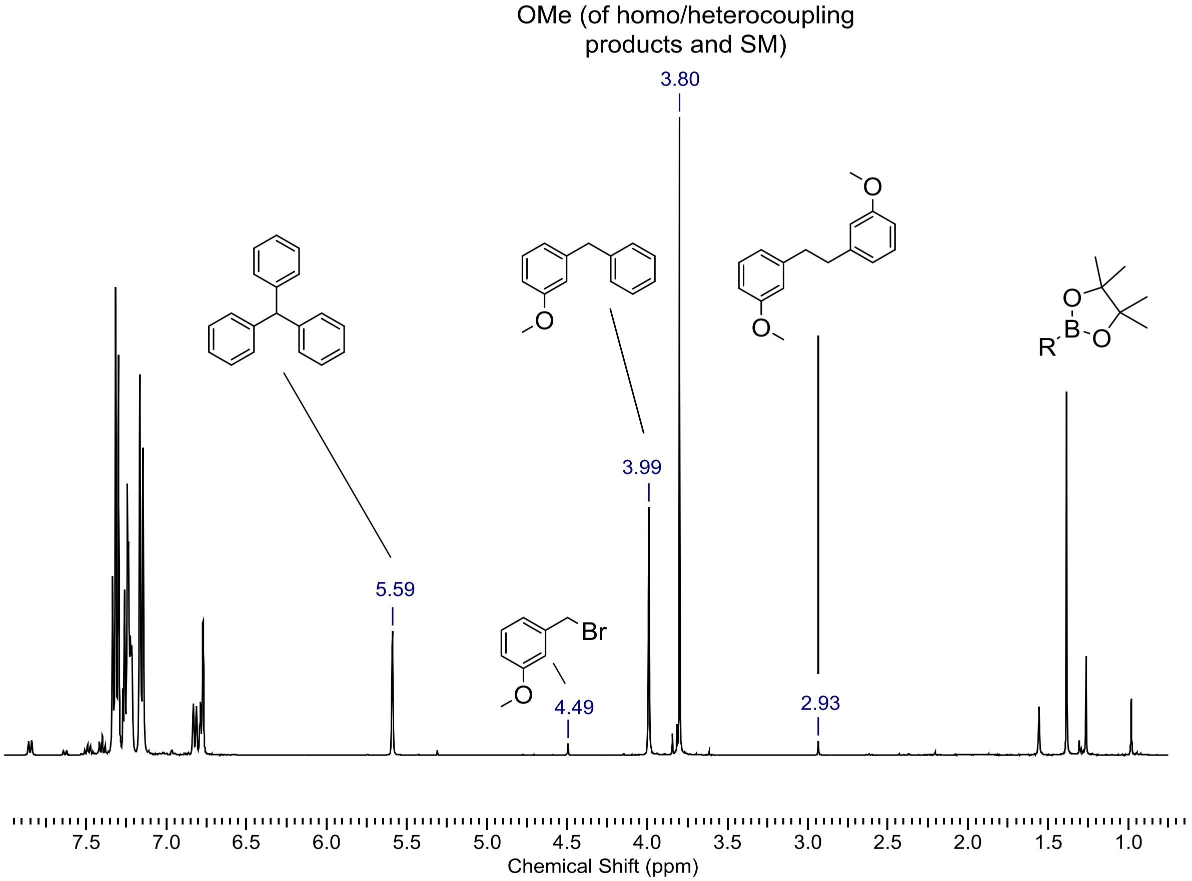

Figure S 102: ^{1}H NMR spectrum of the crude from the reaction to produce 3a

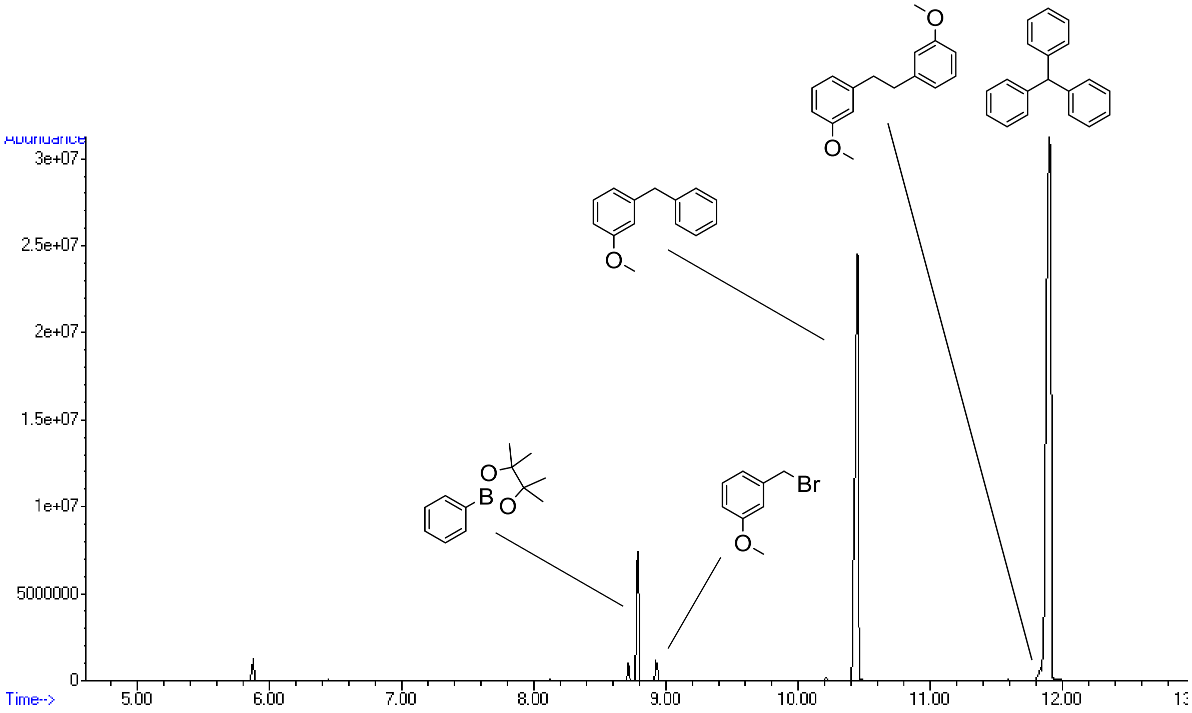

Figure S 113: GCMS chromatogram of crude reaction mixture from production of 3a

1-benzyl 4-fluorobenzene (3b)

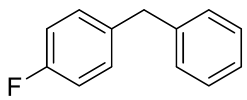

Synthesised according to the above general procedure from 4-fluorobenzyl bromide (44 μl) and [Li][(^{t}Bu)(Ph)B(Pin)] (141 mg). ^{1}H NMR spectroscopic data is consistent with previously reported values[6]. Yield 85% using triphenylmethane as an internal standard.

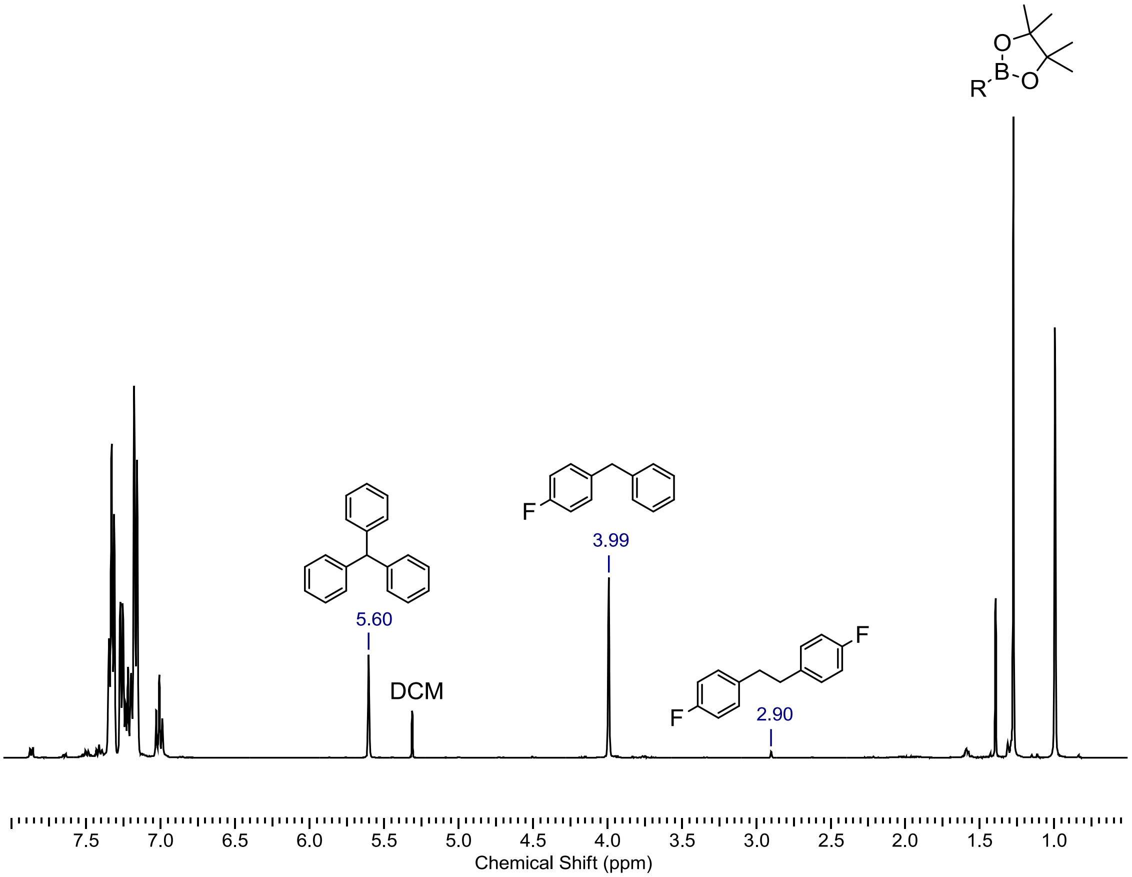

Figure S 124: ^{1}H NMR spectrum of the crude from the reaction to produce 3b

1-benzyl 4-bromobenzene (3c)

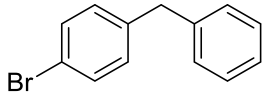

Synthesised according to the above general procedure from 4-bromobenzyl bromide (87.5 mg) and [Li][(^{t}Bu)(Ph)B(Pin)] (141 mg). ^{1}H NMR spectroscopic data is consistent with previously reported values[5]. Yield 75% using triphenylmethane as an internal standard.

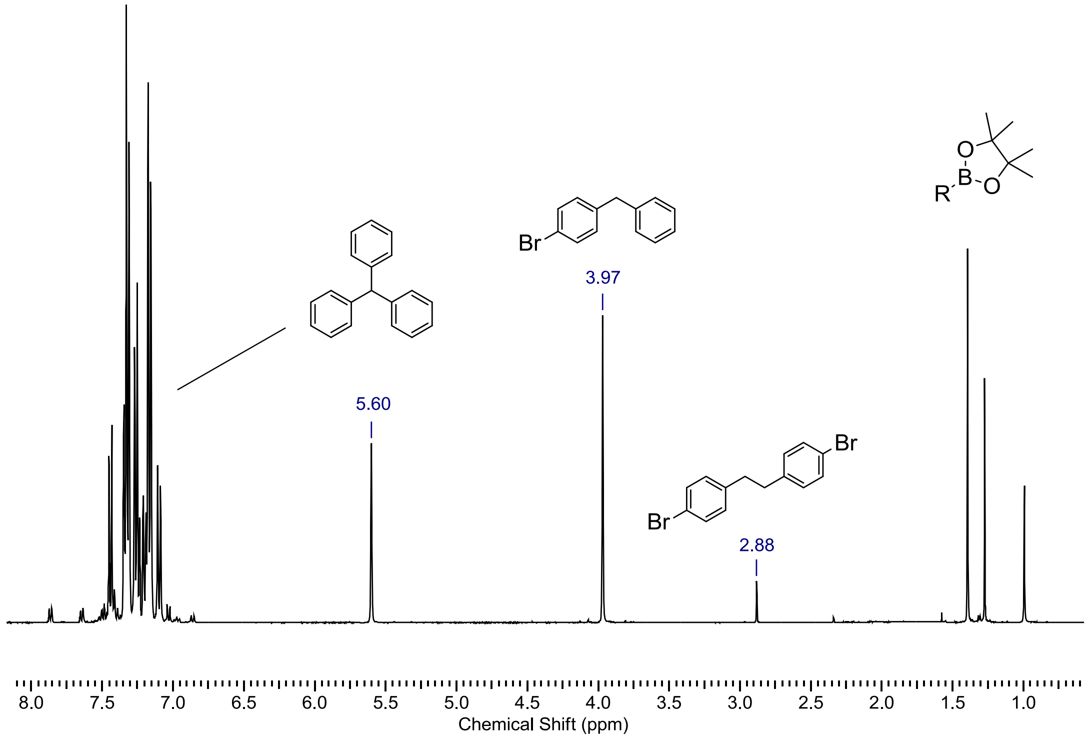

Figure S 135: ^{1}H NMR spectrum of the crude from the reaction to produce 3c

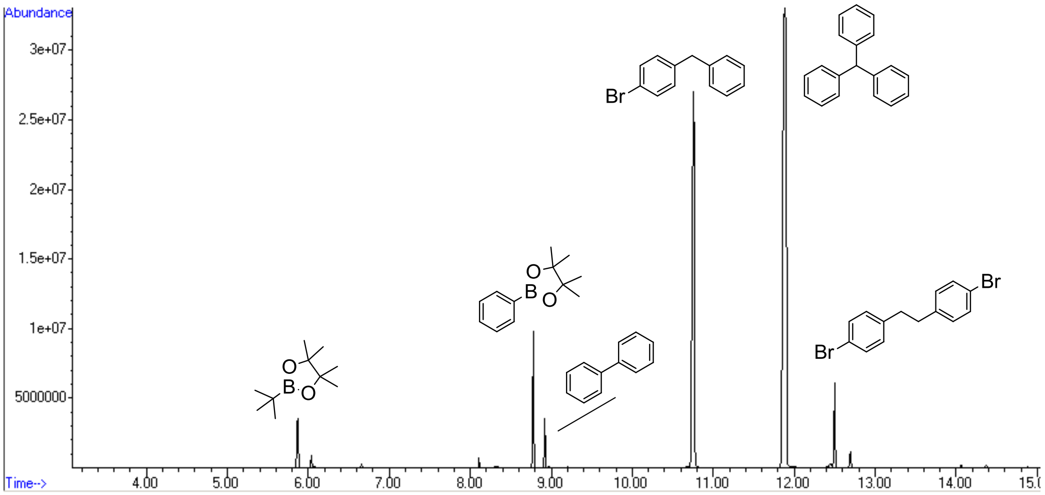

Figure S 146: GCMS chromatogram of the crude from the reaction to produce 3c

Diphenylmethane (3d)

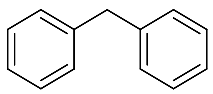

Synthesised according to the above general procedure from benzyl bromide (42 μl) and [Li][(^{t}Bu)(Ph)B(Pin)] (141 mg). ^{1}H NMR spectroscopic data is consistent with previously reported values[7]. Yield 82% using triphenylmethane as an internal standard. Alternatively synthesised according to the above general procedure from benzyl chloride and [Li][(^{t}Bu)(Ph)B(Pin)]. Yield 30 %

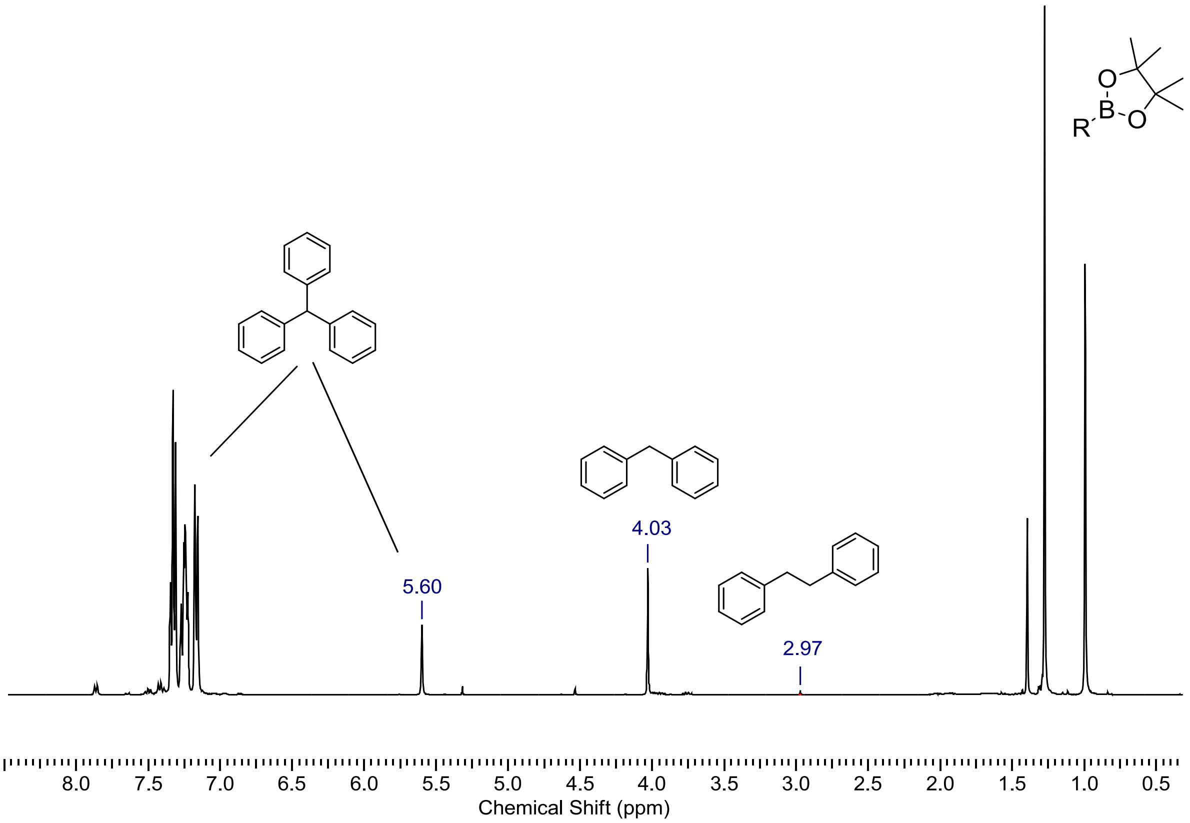

Figure S 157: ^{1}H NMR spectrum of the crude from the reaction to produce 3d

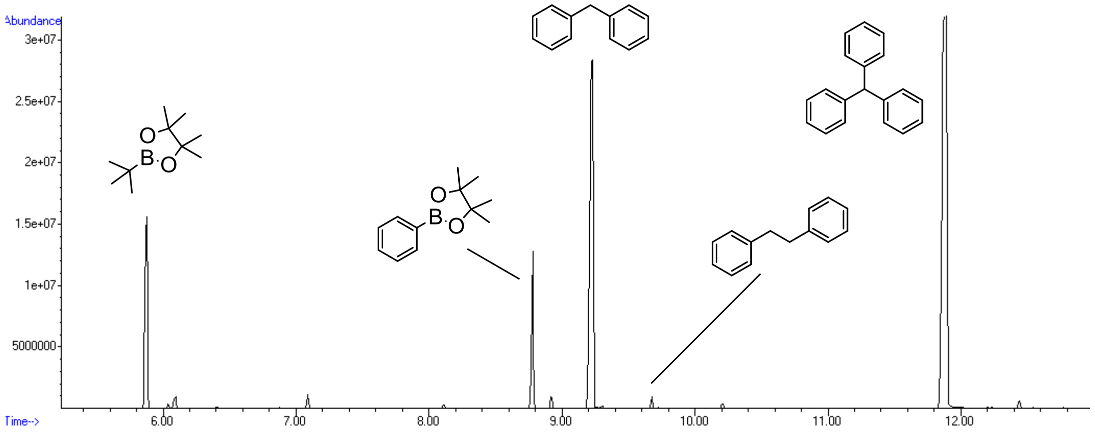

Figure S 168: GCMS chromatogram of the crude from the reaction to produce 3d

1-benzyl 4-(trifluoromethyl)benzene (3e)

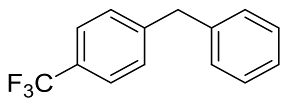

Synthesised according to the above general procedure from 4-(trifluoromethyl)benzyl bromide (54 µl) and [Li][(^{t}Bu)(Ph)B(Pin)] (141 mg). ^{1}H NMR spectroscopic data is consistent with previously reported values[6]. Yield 69% using triphenylmethane as an internal standard.

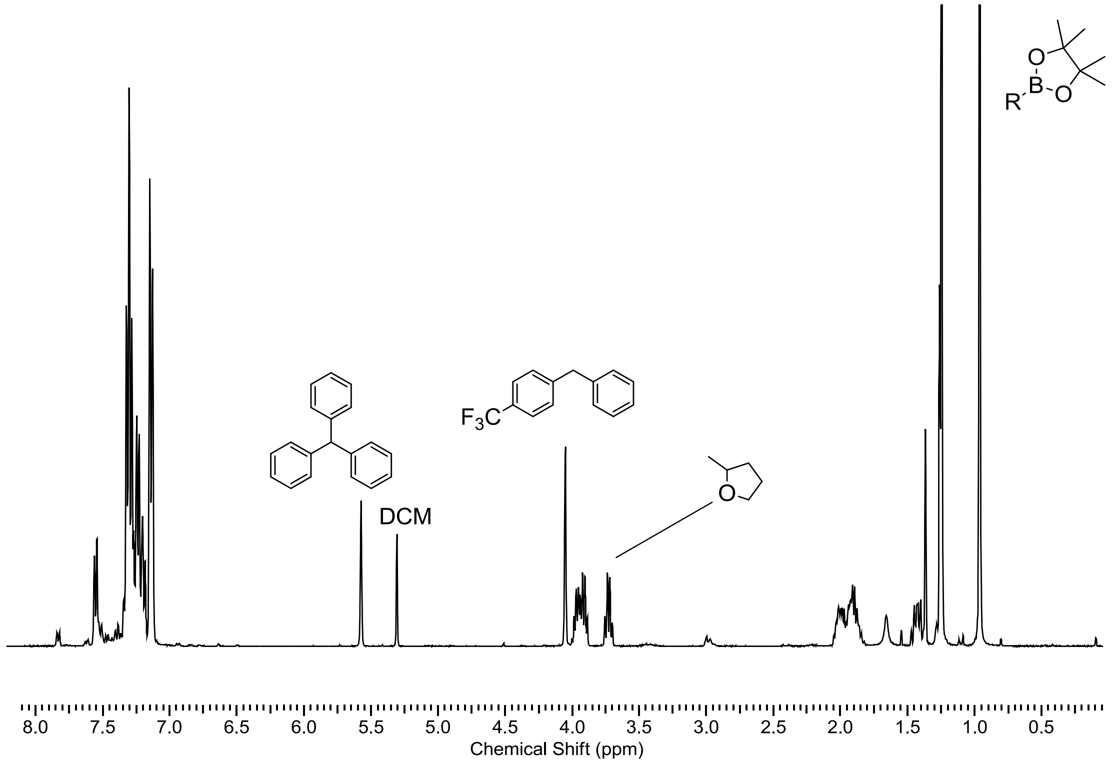

Figure S 179: ^{1}H NMR spectrum of the crude from the reaction to produce 3e

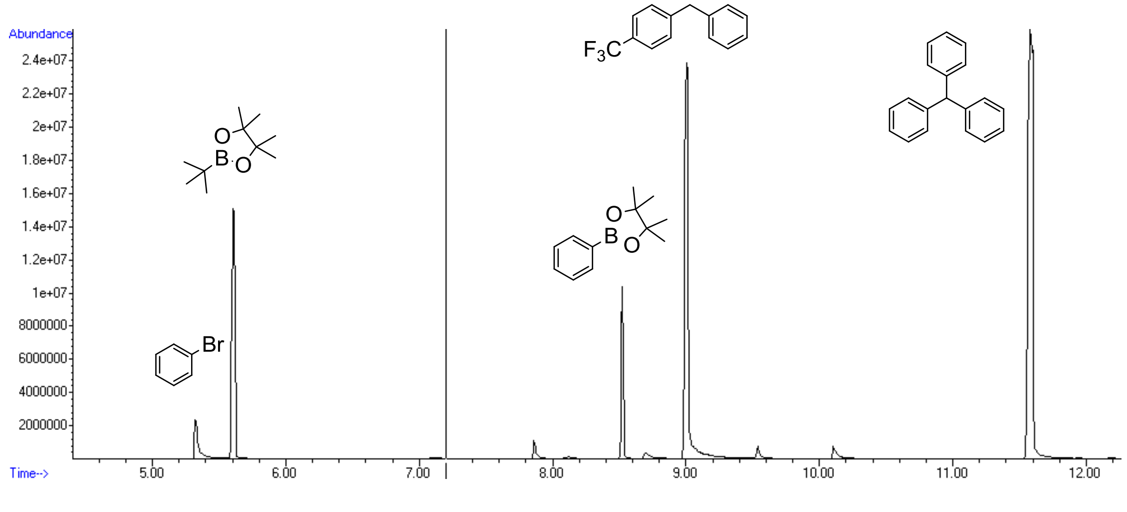

Figure S 2018: GCMS chromatogram of the crude from the reaction to produce 3e

1-benzyl 4-methylbenzene (3f)

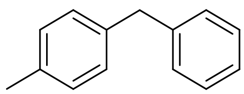

Synthesised according to the above general procedure from 4-methylbenzyl bromide and [Li][(^{t}Bu)(Ph)B(Pin)]. ^{1}H NMR spectroscopic data is consistent with previously reported values[5]. Yield 85% using triphenylmethane as an internal standard.

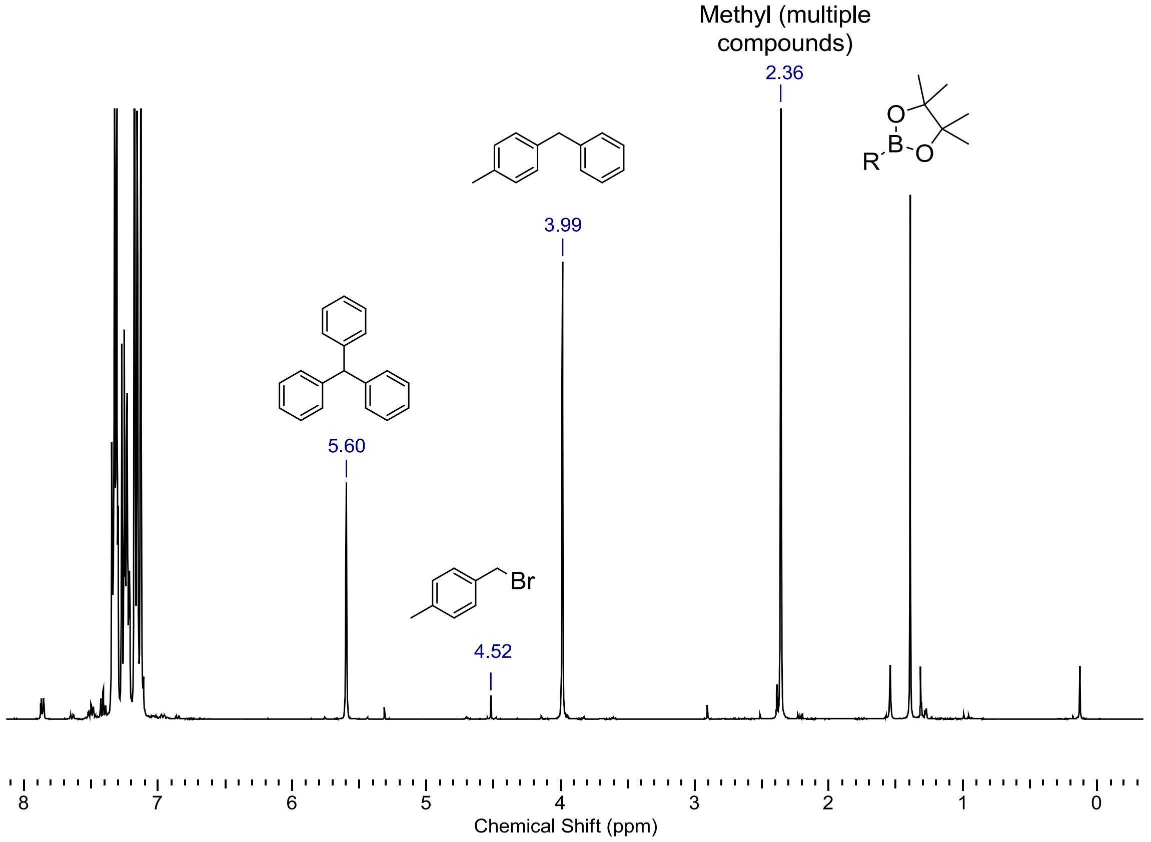

Figure S 19: ^{1}H NMR spectrum of the crude from the reaction to produce 3f

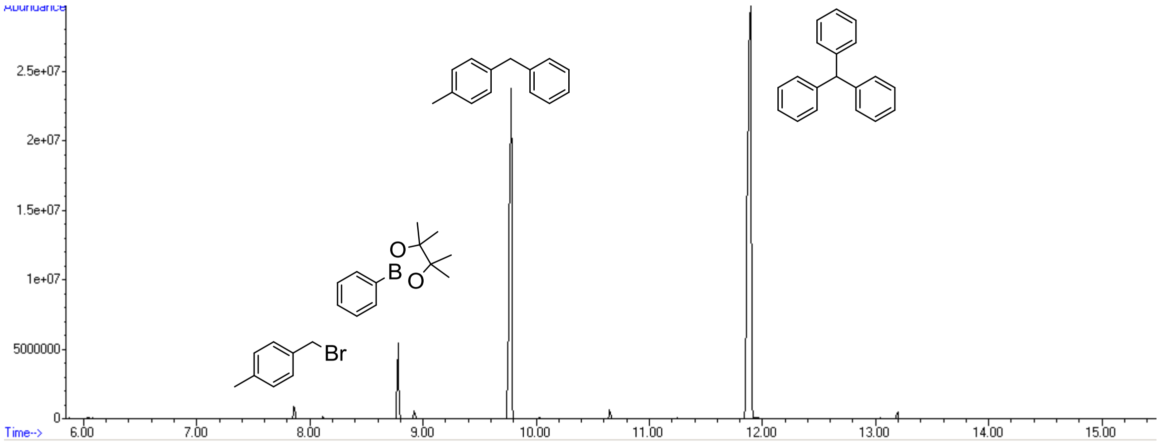

Figure S 20: GCMS chromatogram of the crude from the reaction to produce 3f

Methyl 4-benzylbenzoate (3g)

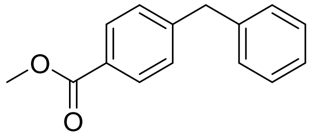

Synthesised according to the above general procedure from methyl 4-(bromomethyl)benzoate (80 mg) and [Li][(^{t}Bu)(Ph)B(Pin)] (141 mg), with heating to 60°C for 72 hours. After this time the solvent was removed *in vacuo* without HCl quench. ^{1}H NMR spectroscopic data is consistent with previously reported values[5]. Yield: 58% using triphenylmethane as an internal standard.

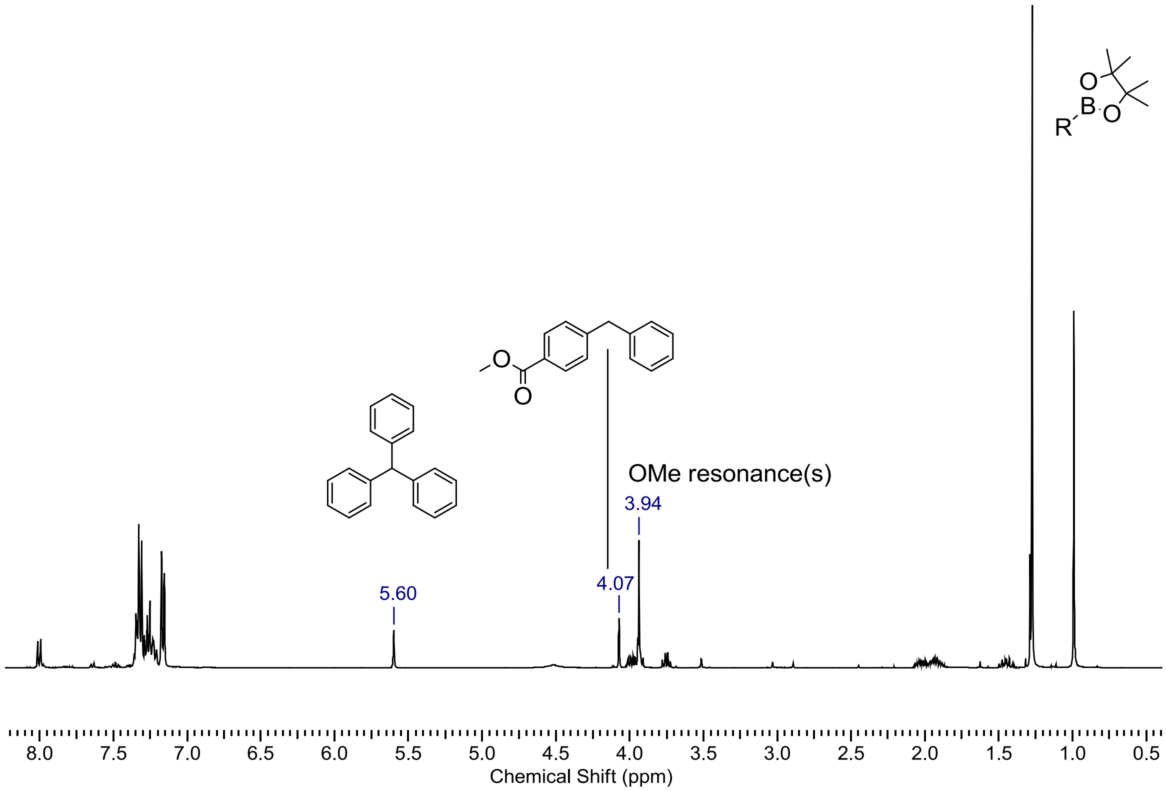

Figure S 213: ^{1}H NMR spectrum of the crude from the reaction to produce 3g

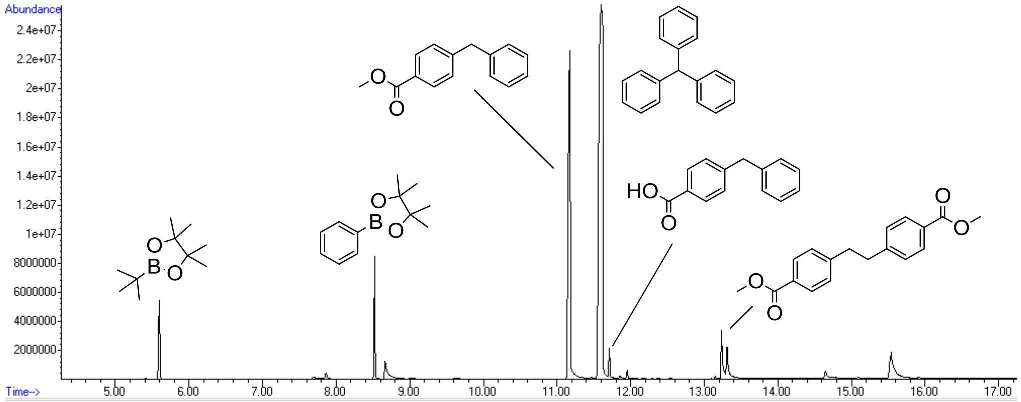

Figure S 224: GCMS chromatogram of the crude from the reaction to produce 3g

1-benzyl-4-methylsulfanyl-benzene (3h)

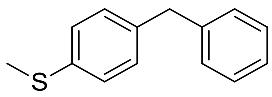

Synthesised according to the above general procedure from 4-(bromomethyl)phenyl methyl sulphide (76 mg) and [Li][(^{t}Bu)(Ph)B(Pin)] (141 mg). ^{1}H NMR spectroscopic data is consistent with previously reported values[6]. Yield: 64% using triphenylmethane as an internal standard.

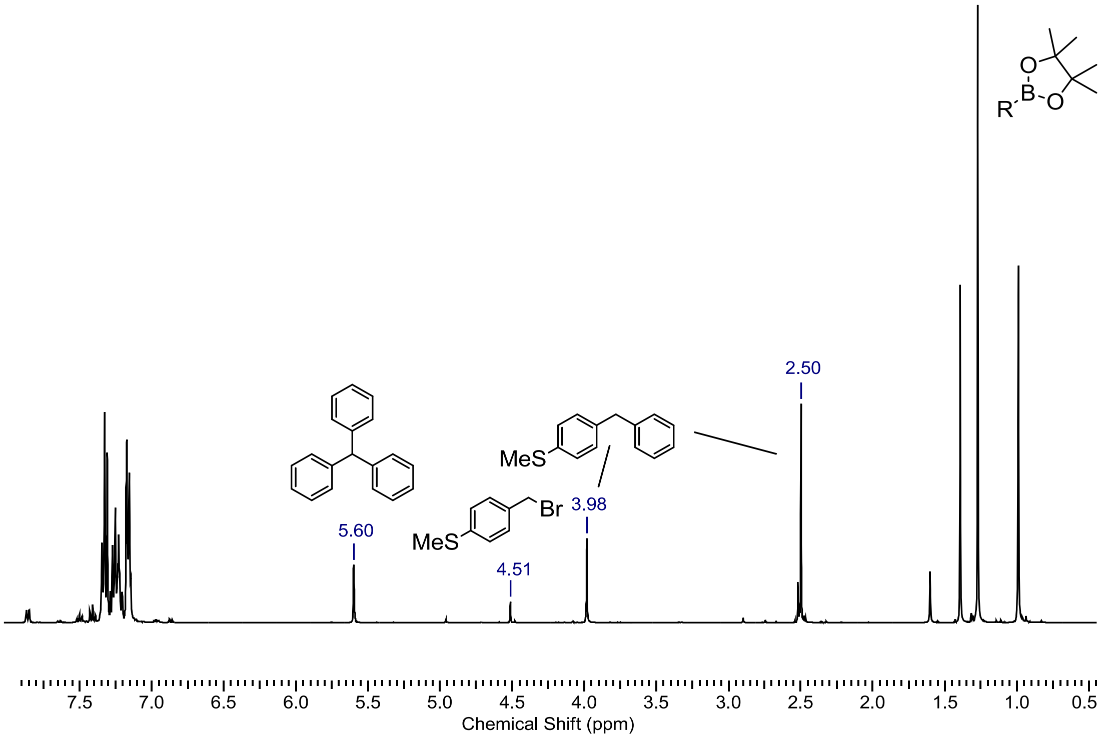

Figure S 23: ^{1}H NMR spectrum of the crude from the reaction to produce 3h

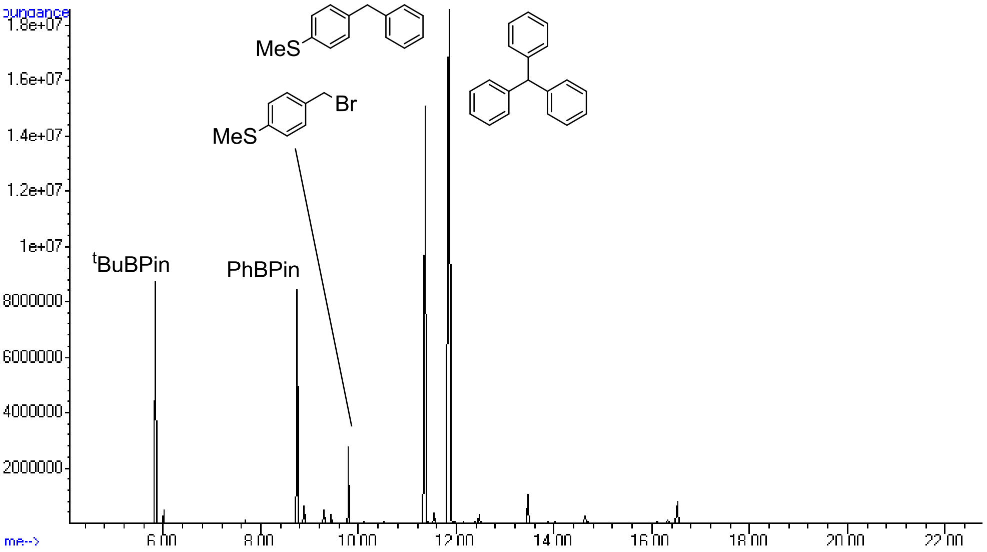

Figure S 24: GCMS chromatogram of the crude from the reaction to produce 3h

5-(phenyl)-1,3-benzodioxole (3i)

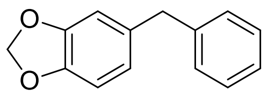

Synthesised according to the above general procedure from 5-(bromomethyl)-1,3-benzodioxole (75 mg) and [Li][(^{t}Bu)(Ph)B(Pin)] (141 mg). ^{1}H NMR spectroscopic data is consistent with previously reported values[7]. Yield: 86% using triphenylmethane as an internal standard.

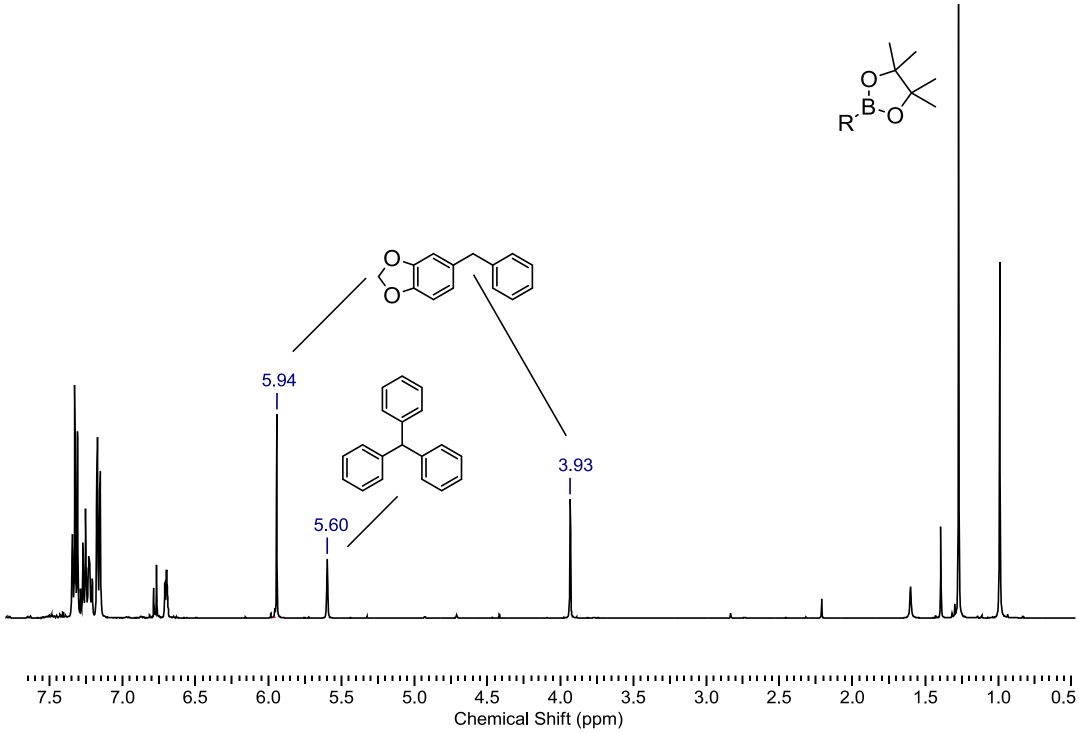

Figure S 25: ^{1}H NMR spectrum of the crude from the reaction to produce 3i

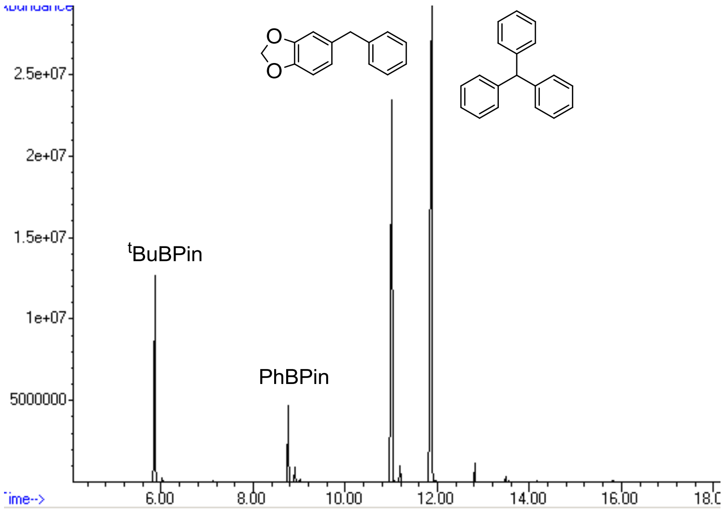

Figure S 26: GCMS chromatogram of the crude from the reaction to produce 3i

1-fluoro 4-(4-methoxybenzyl)benzene (3j)

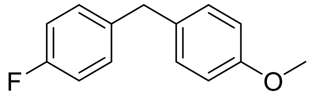

Synthesised according to the above general procedure from 4-fluorobenzyl bromide (44 μl) and [Li][(^{t}Bu)(MeO-C_6H_4-)B(Pin)] (157 mg). ^{1}H NMR spectroscopic data is consistent with previously reported values[8]. Yield 87% using triphenylmethane as an internal standard.

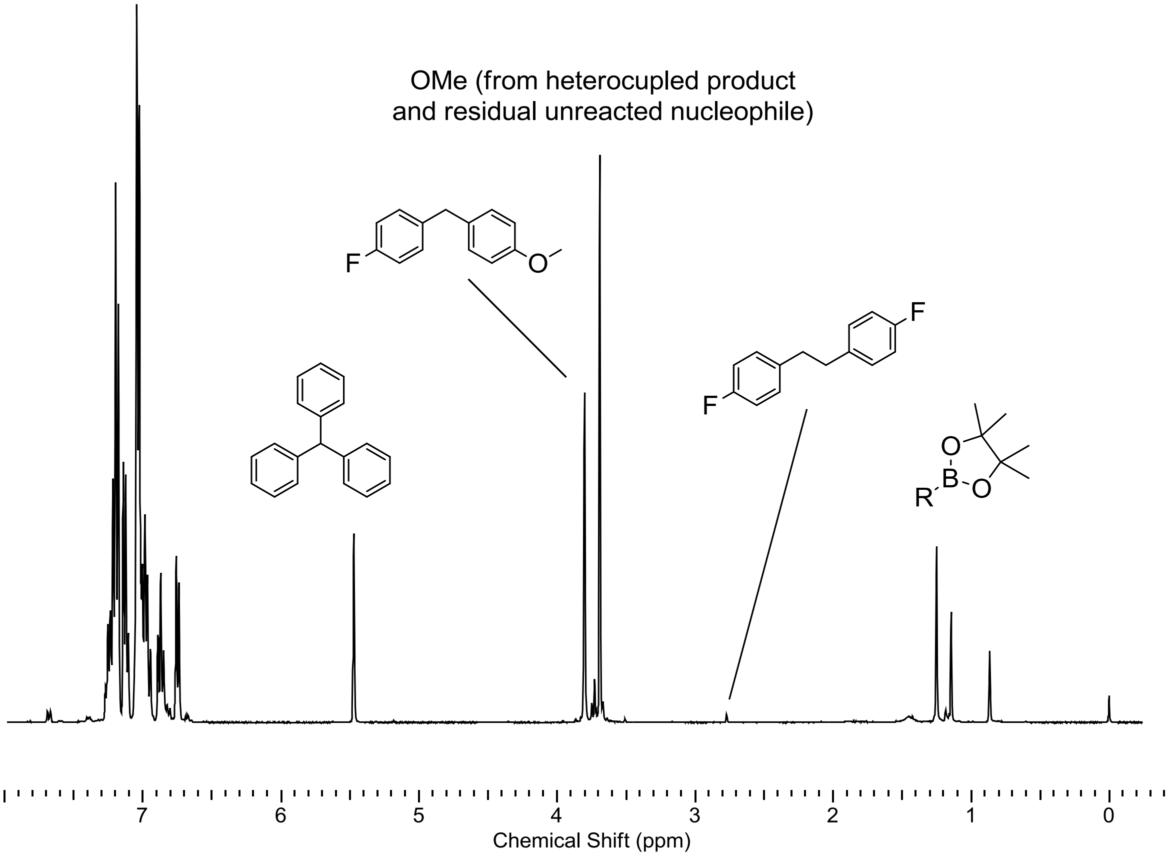

Figure S 279: ^{1}H NMR spectrum of the crude from the reaction to produce 3j

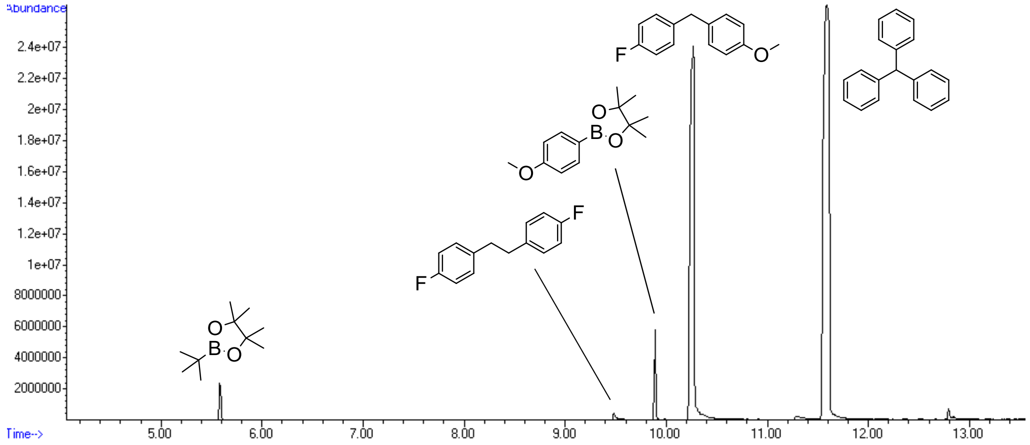

Figure S 3028: GCMS chromatogram of the crude from the reaction to produce 3j

1-methyl-4-(4-(trifluoromethyl)benzyl)benzene (3k)

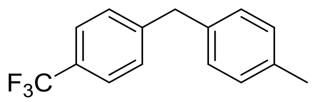

Synthesised according to the above general procedure from 4-(trifluoromethyl)benzyl bromide (54 μl) and [Li][(^{t}Bu)(p-tol)B(Pin)] (148 mg). ^{1}H NMR spectroscopic data is consistent with previously reported values[9]. Yield 75% using triphenylmethane as an internal standard.

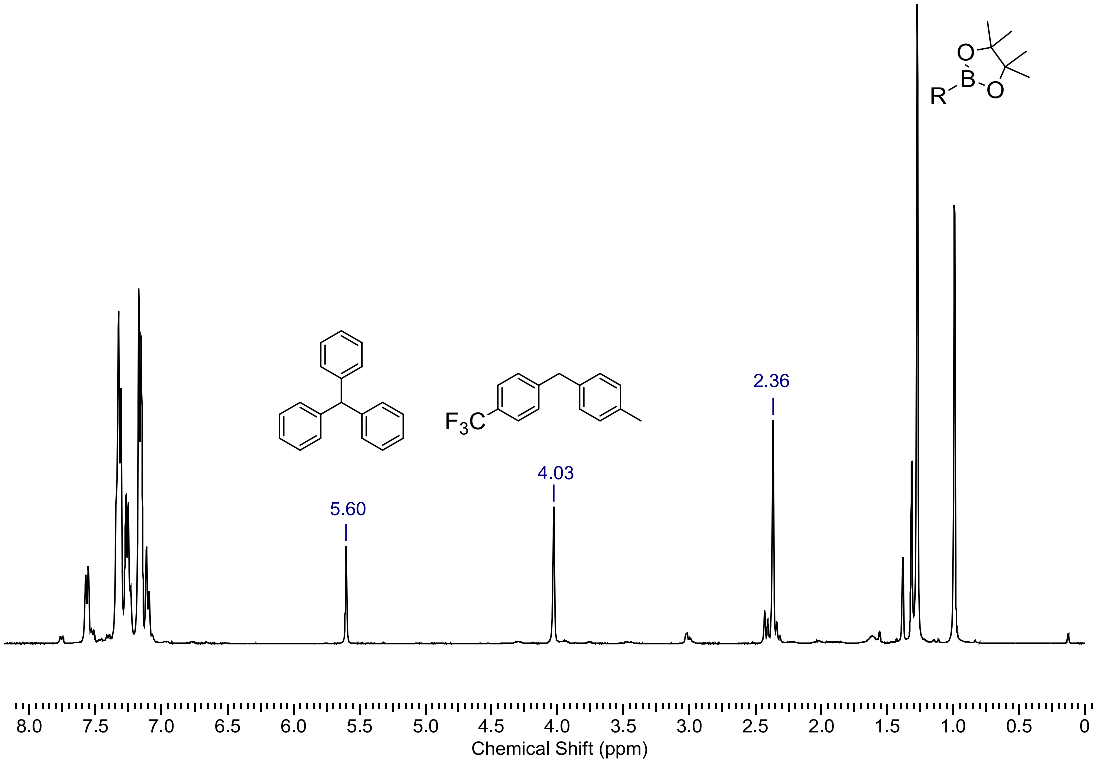

Figure S31: ^{1}H NMR spectrum of the crude from the reaction to produce 3k

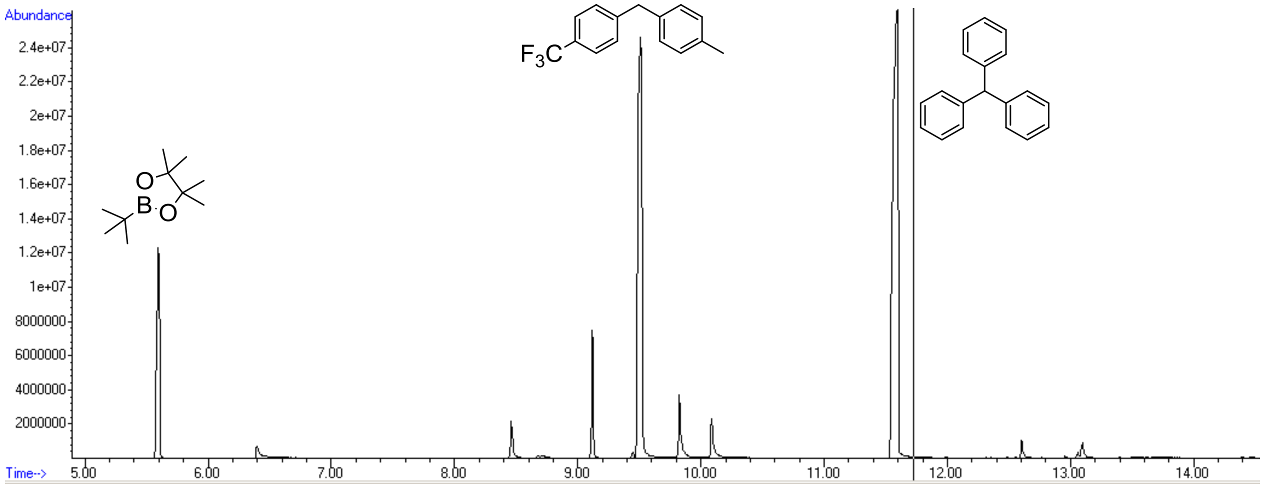

Figure S 32: GCMS chromatogram of the crude from the reaction to produce 3k

2-(4-fluorobenzyl)-5-methylthiophene (3l)

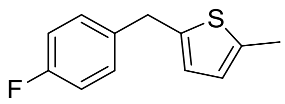

Synthesised according to the above general procedure from 4-fluorobenzyl bromide and crude [Li][(^{t}Bu)(Me-C_4H_2S)B(Pin)] (1e). Purification of the crude mixture by silica gel column chromatography was attempted (using PET ether as eluent), although NMR analysis showed the product was present (25 mg 75 % yield) in 87% purity (therefore yield of the desired C2 functionalised = 65%), due to the presence of 13 % of the 3-isomer from Friedel Crafts functionalisation of the beta thiophene position, related chemistry has been observed previously using anisole-zinc Lewis acid reagents[10].

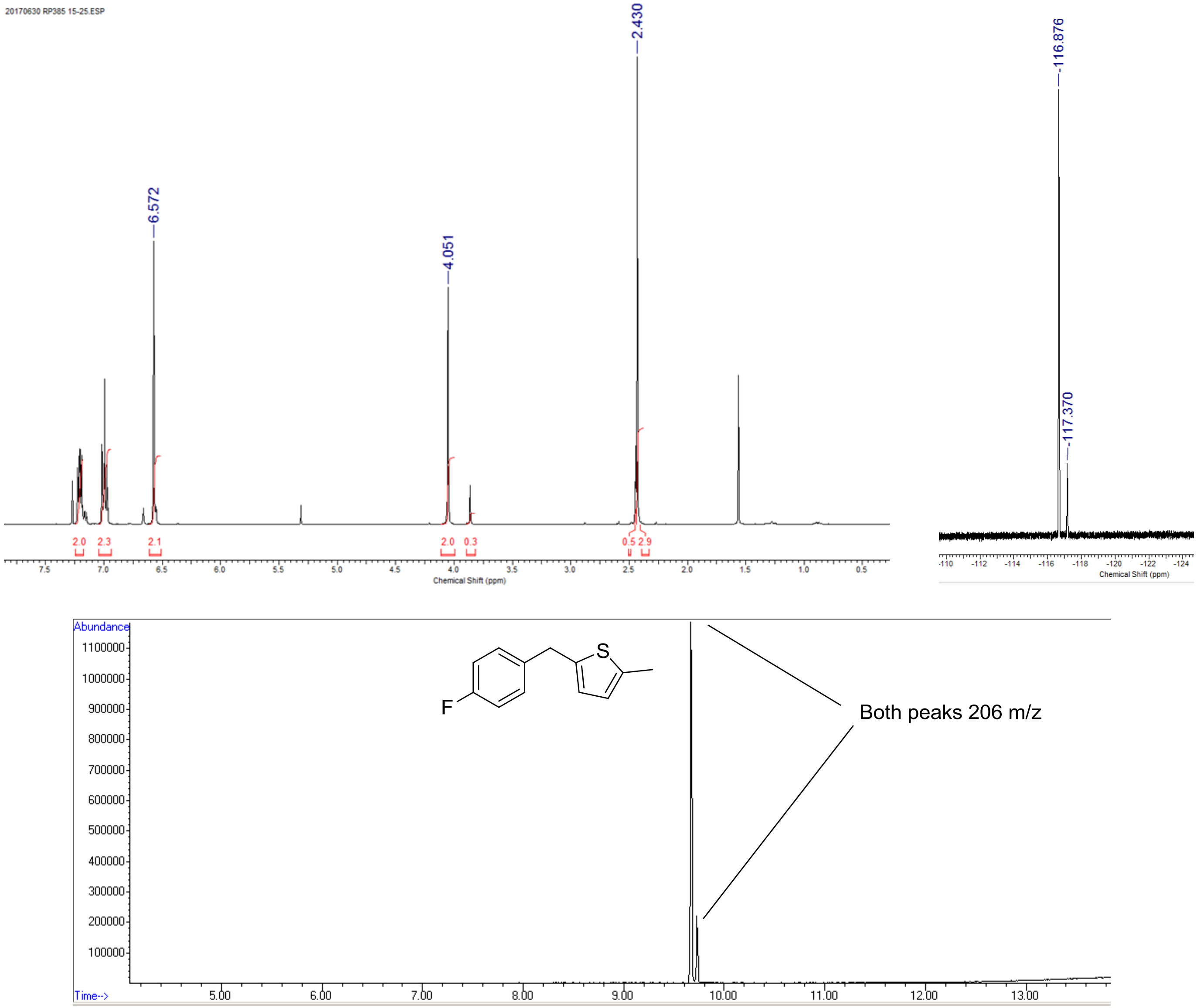

.

Figure S 33: Top left, ^{1}H NMR spectrum of the columned products from the reaction to produce 3l. Top right, ^{19}F NMR spectrum. Bottom, GCMS chromatogram of the reaction products from 3l

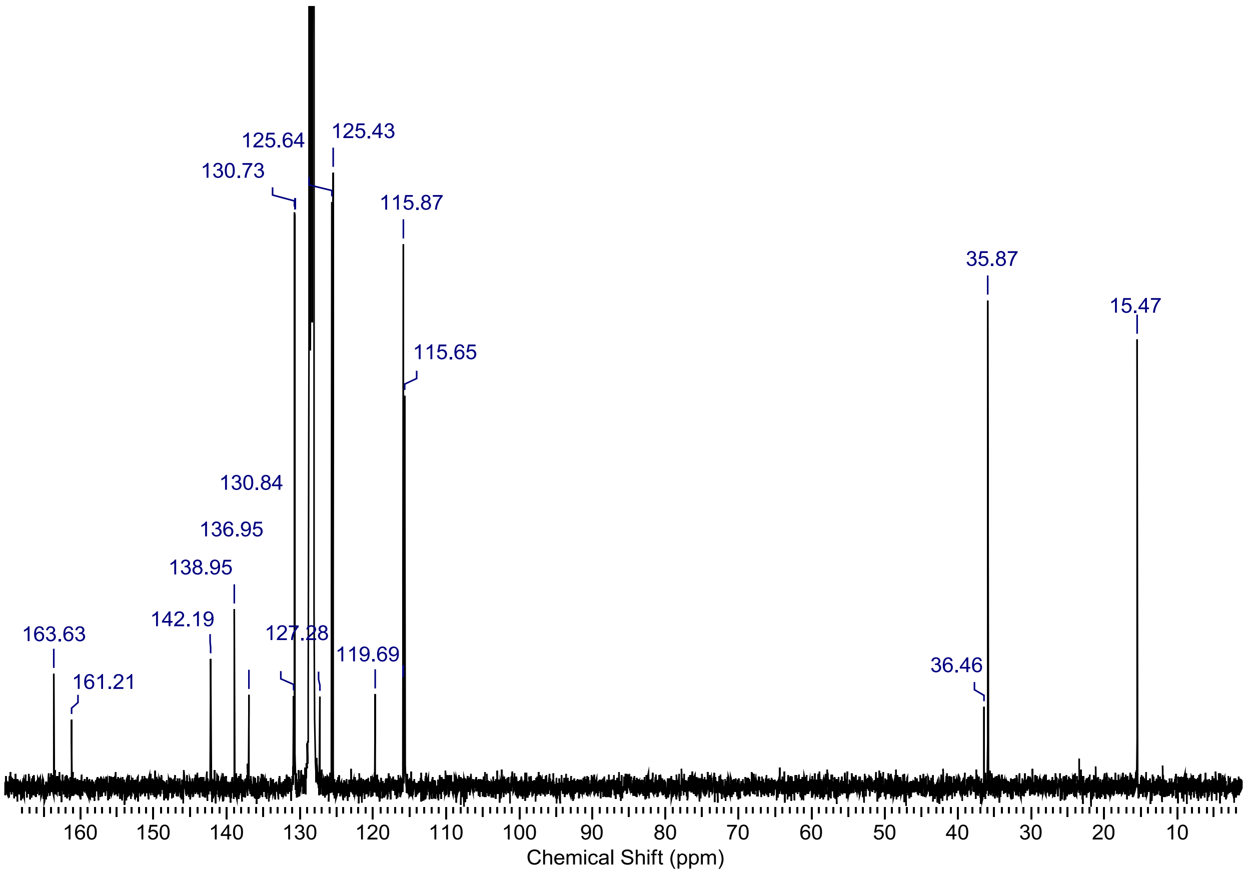

Figure S 29: $^{13}C\{^1H\}$ (C_6D_6) NMR spectrum of the products from 3l

The major isomer is assigned as the 2-(4-fluorobenzyl)-5-methyl isomer, while the minor isomer is assigned as the 3-(4-fluorobenzyl)-5-methyl isomer, based on two observations. Firstly, ^{1}H NMR data for the analogous 2-phenyl-5-methyl- isomer has been previously published[11], and this shows strong similarity to the major isomer of 3l, particularly the CH_2 benzyl resonances (4.07 vs 4.05 ppm). Additionally, HMBC shows that for the minor isomer, where each thienyl resonance is distinct, there is coupling between the benzyl protons and both thienyl carbon (containing a C-H) resonance, which would be much more likely to occur in the 3-(4-fluorobenzyl)-5-methyl isomer, as both of these positions are only separated by 3 bonds, whereas for the 2-(4-fluorobenzyl) isomer, one is separated by 4 bonds. However, since the thienyl resonances of the major isomer are coincident, completely unambiguous assignment is not possible. Finally, in the work reported herein using anisole derivatives very little Freidel Crafts substitution products are observed in contrast to previous work,[10] indicating the organometallic coupling is the preferred process in this work.

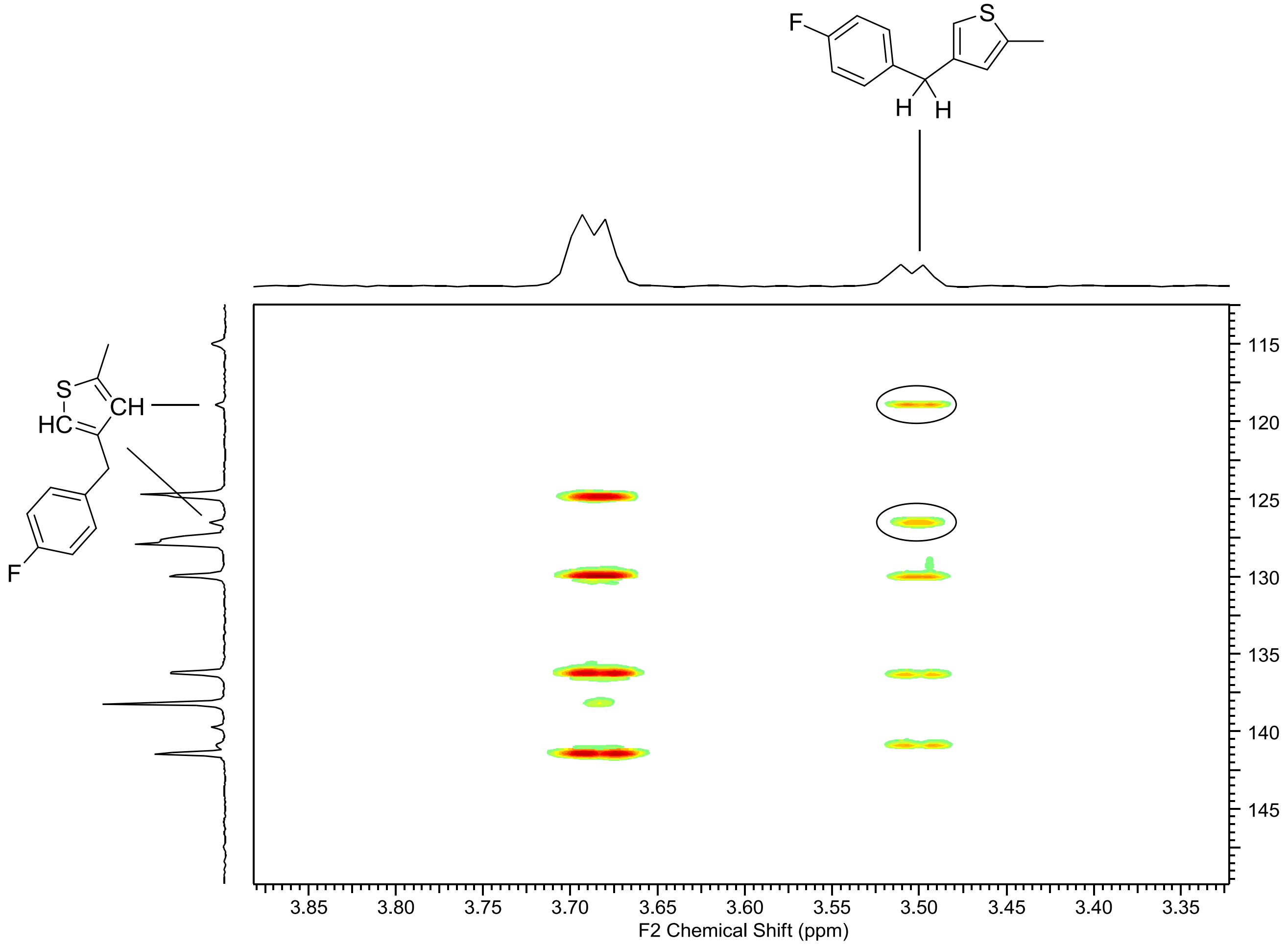

Figure S 30: Aryl region of the HMBC spectrum of the products 3l. Minor product benzyl ^{1}H resonance at 3.5 ppm, major product benzyl ^{1}H resonance at 3.7

1-fluoro-4-(4-methylbenzyl)benzene (3m)

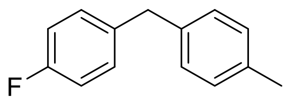

Synthesised according to the above general procedure from 4-fluorobenzyl bromide (44 μl) and [Li][(^{t}Bu)(p-tol)B(Pin)] (148 mg). ^{1}H NMR spectroscopic data is consistent with previously reported values[5]. Yield 90% using triphenylmethane as an internal standard.

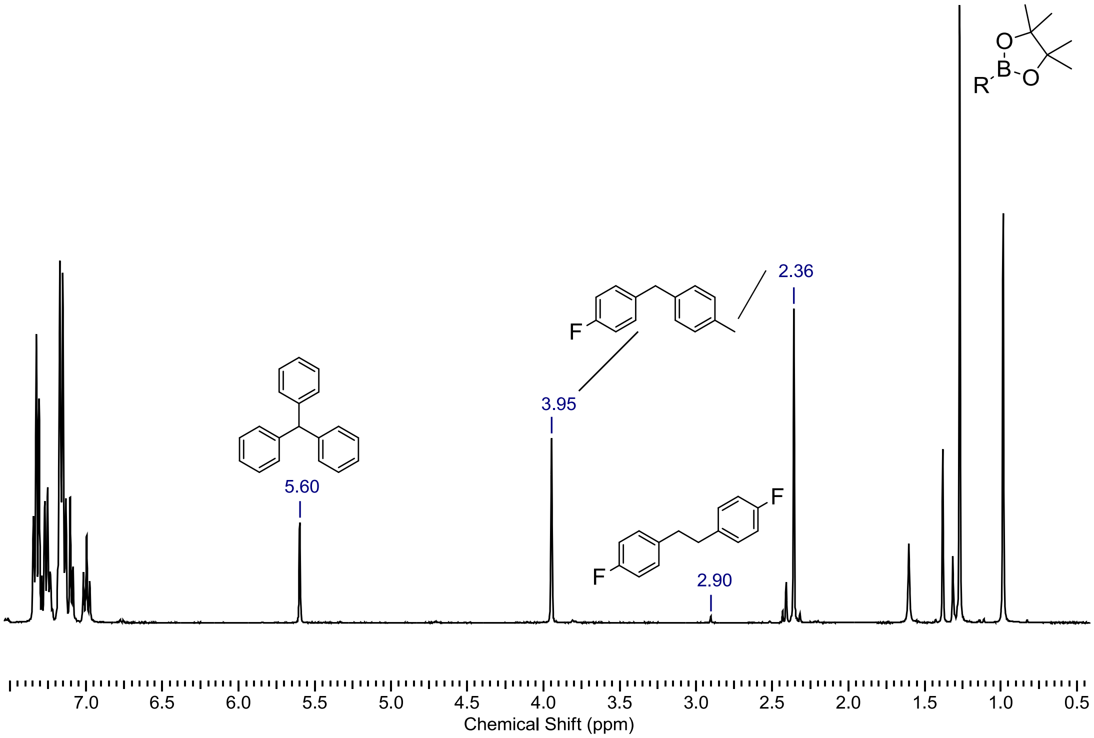

Figure S 31: ^{1}H NMR spectrum of the crude from the reaction to produce 3m

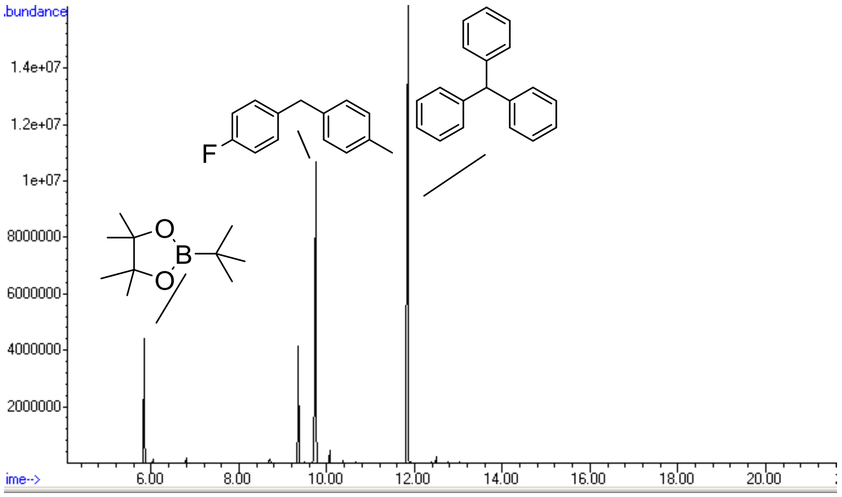

Figure S 32: GCMS chromatogram of the crude from the reaction to produce 3m

1,1-diphenylethane (3n)

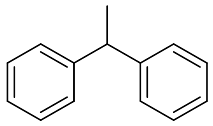

Synthesised according to the above general procedure from (1-bromoethyl)benzene (48 µl) and [Li][(^{t}Bu)(Ph)B(Pin)] (141 mg), with heating to 60°C for 72 hours. ^{1}H NMR spectroscopic data is consistent with previously reported values[12]. Yield 59% using triphenylmethane as an internal standard.

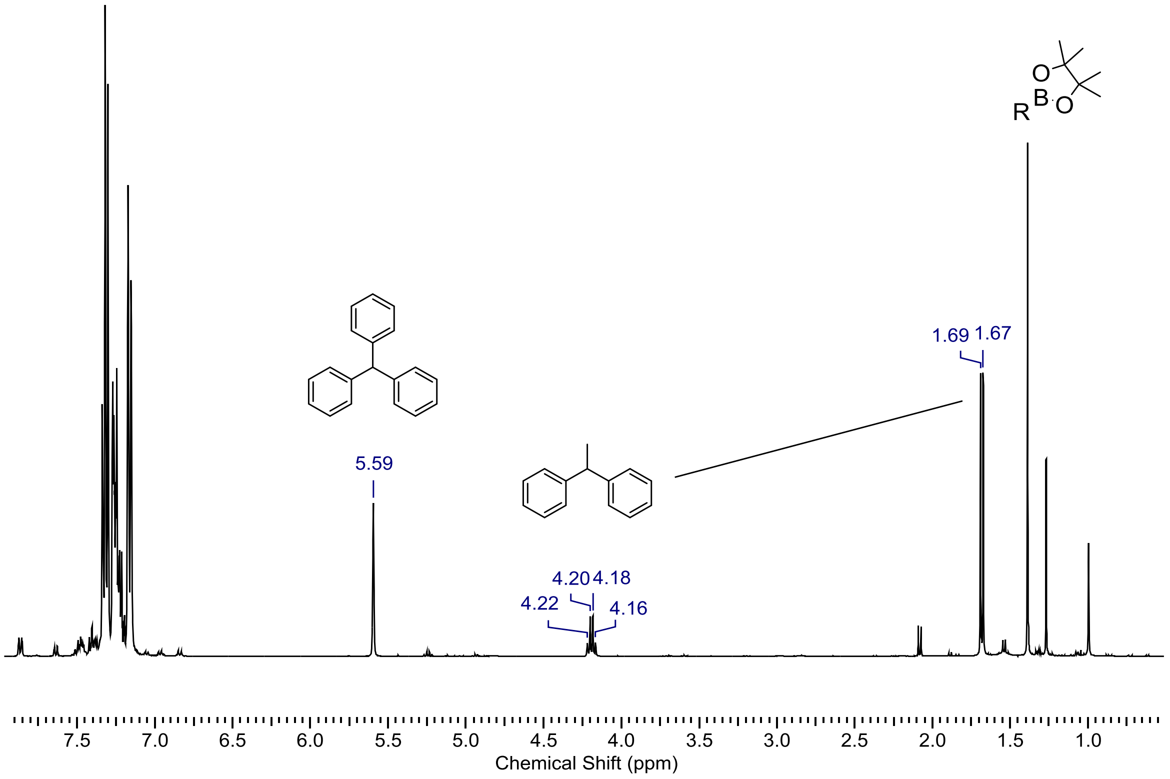

Figure S 33: ^{1}H NMR spectrum of the crude from the reaction to produce 3n

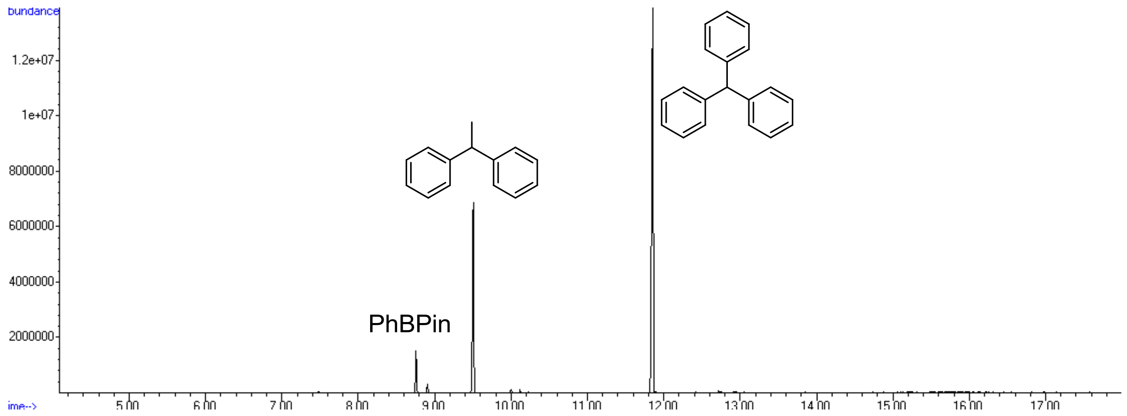

Figure S 349: GCMS chromatogram of the crude from reaction to produce 3n

((4-methoxyphenyl)methylene)dibenzene (3o)

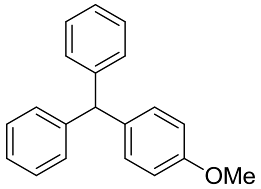

Synthesised according to the general procedure from diphenylbromomethane (87 mg) and [Li][(^{t}Bu)(*p*MeO-C_6H_4)B(Pin)] (157 mg), using mesitylene as an internal standard. ^{1}H NMR spectroscopic data is consistent with previously reported values[13]. Yield: 60%. A minor isomer is observed by GCMS, but the ^{1}H NMR spectrum of other *OMe*- isomers are significantly different,[14] thus assignment of the major isomer as the *para*- product from organometallic cross coupling is unambiguous and consistent with previous work.[10]

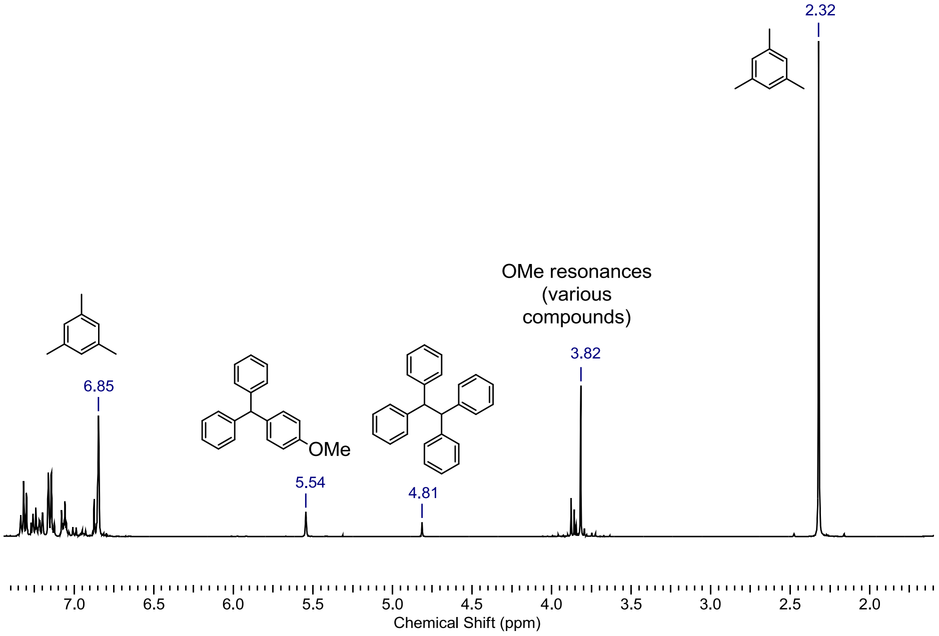

Figure S 40: ^{1}H NMR spectrum of the crude from the reaction to produce 3o

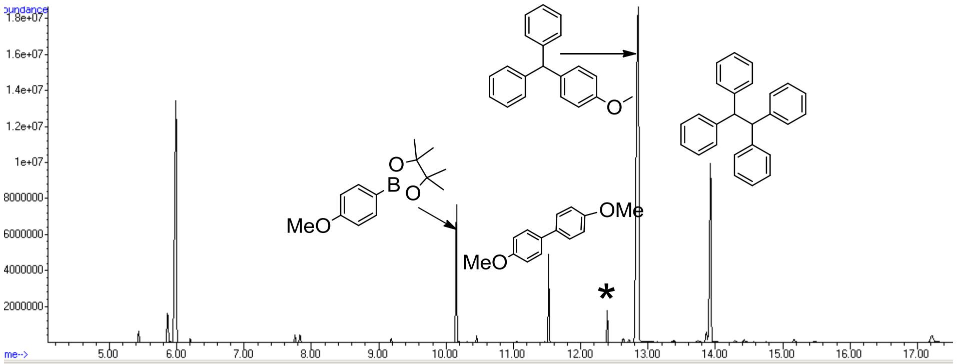

Figure S 41 : GCMS chromatogram of the crude from the reaction to produce 3o. * minor isomer

Ratio of major to minor isomer (from GC-MS))) = 30:1

2-Methyl-3-phenyl-1-propene (3p)

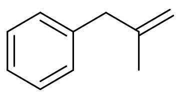

Synthesised according to the above general procedure from 3-bromo-2-methylpropene (35 μl) and [Li][(^{t}Bu)(Ph)B(Pin)] (141 mg). ^{1}H NMR spectroscopic data is consistent with previously reported values_ENREF_14[15]. Yield: 60% using triphenylmethane as an internal standard.

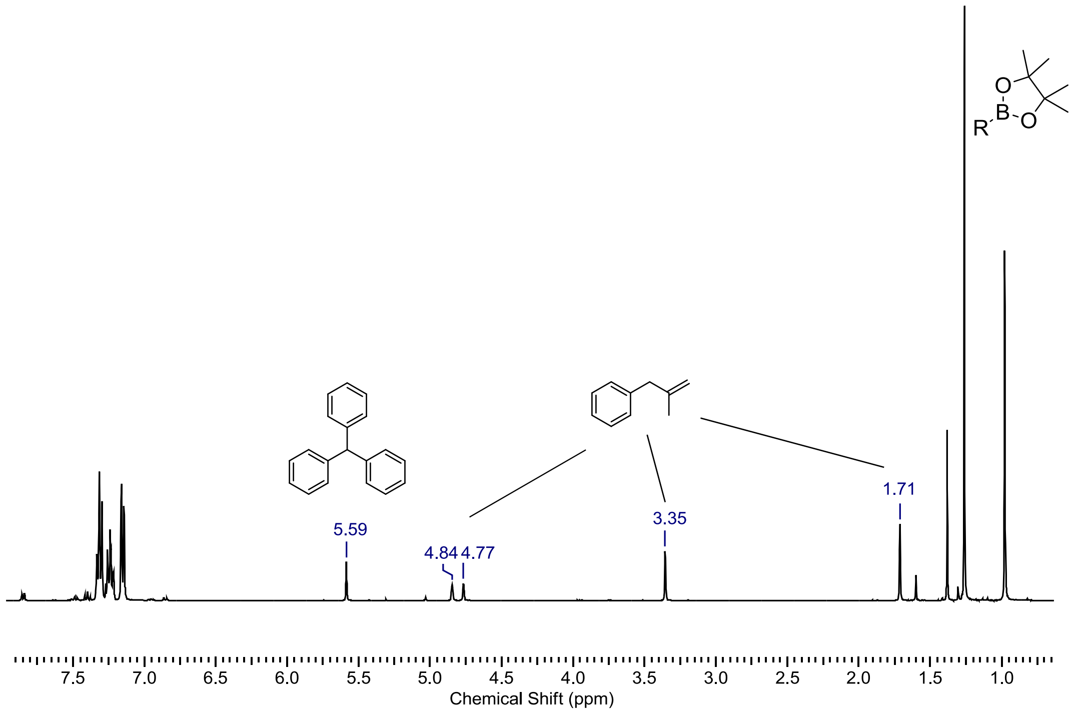

Figure S 42: ^{1}H NMR spectrum of the crude from the reaction to produce 3p

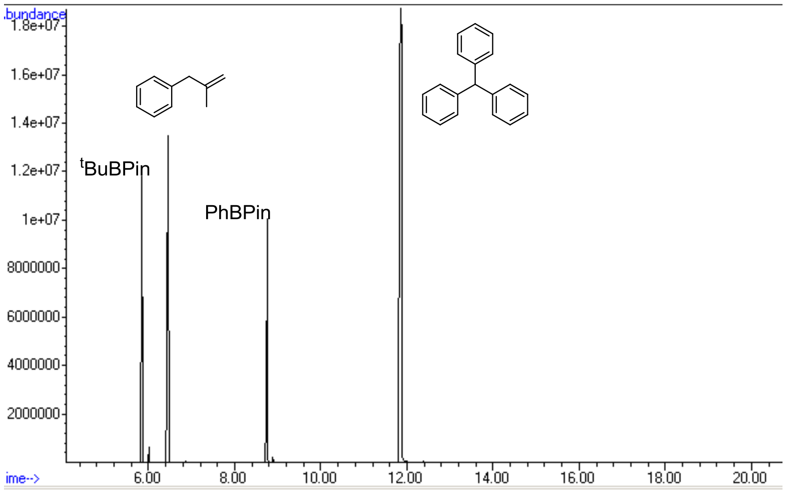

Figure 43: GCMS chromatogram of the crude from the reaction to produce 3p

1-fluoro-4-(4-(trifluoromethoxy)benzyl)benzene (3q)

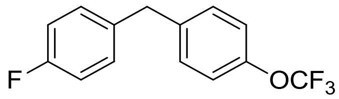

Synthesised according to the above general procedure from 4-fluorobenzyl bromide (22 µl) and [Li][(tBu)(F_3CO-C_6H_4-)B(Pin)] (90 mg) and 325 µl of a 0.047 M solution of $ZnBr_2$ (10 mol%). The reaction was quenched with ethanol and the conversion was measured by ^{19}F NMR spectroscopy. Conversion : 30% after 24 hours.

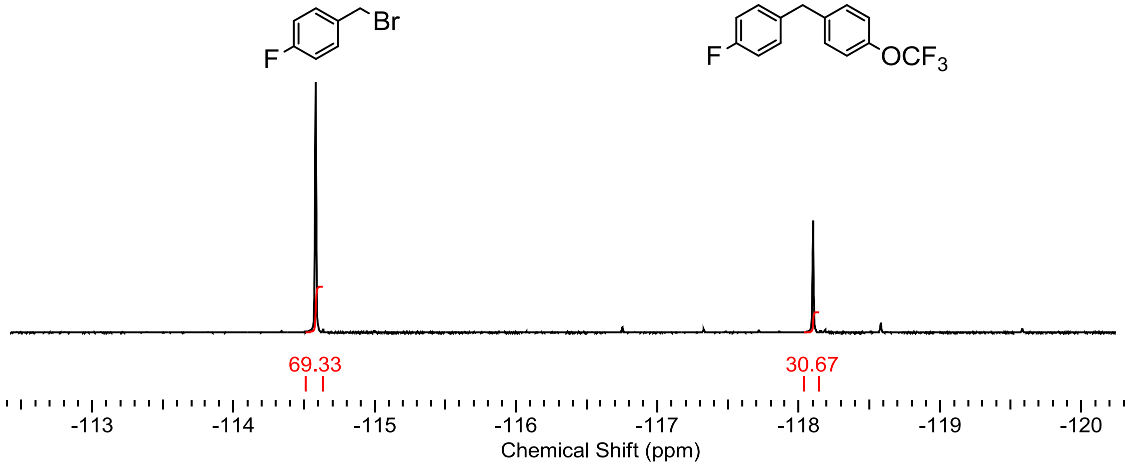

Figure S44: ^{19}F NMR spectrum of 3q

Radical inhibition studies

9,10 Dihydroanthracene

In an oven dried ampoule 9,10 dihydroanthracene (42 mg, 0.23 mmol, 1eq.) and [Li][(^{t}Bu)(Ph)B(Pin)] (94 mg, 0.35 mmol, 1.5eq) were dissolved in 1.5ml 2MeTHF. To this was added 500 µl of a 0.047M stock solution of $ZnBr_2$ in 2MeTHF (0.023 mmol, 0.1 eq), immediately followed by 4-fluorobenzyl bromide (29 µl, 0.23 mmol, 1 eq.). The reaction was heated at 60 °C for 18 hours before quenching with ethanol followed by addition of fluorobenzene (22 µl, 0.23 mmol) and mesitylene (32 µl, 0.23 mmol) as standards for analysis. The mixture was directly analysed by ^{19}F NMR spectroscopy before dilution with DCM, filtration through a silica plug and analysis by GCMS.

Styrene

In an oven dried ampoule [Li][(^{t}Bu)(Ph)B(Pin)] (94 mg, 0.35 mmol, 1.5eq) was dissolved in 1.5ml 2MeTHF. To this was added 500 µl of a 0.047M stock solution of $ZnBr_2$ in 2MeTHF (0.023 mmol, 0.1 eq), immediately followed by styrene (27 µl, 0.23 mmol, 1 eq.), and 4-fluorobenzyl bromide (29 µl, 0.23 mmol, 1 eq.). The reaction was heated at 60 °C for 18 hours and 30 minutes before quenching with ethanol followed by addition of fluorobenzene (22 µl, 0.23 mmol) and mesitylene (32 µl, 0.23 mmol) as standards for analysis. The mixture was directly analysed by ^{19}F NMR spectroscopy before dilution with DCM, filtration through a silica plug and analysis by GCMS.

Table S 5: Radical inhibition studies

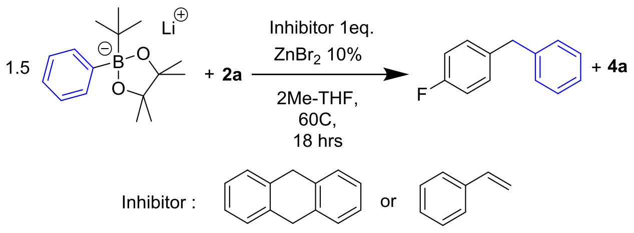

Radical Trap	**3a** / % yield	**4a** / % yield
None	90	1
DHA	92	<1
styrene	91	<1

Zincate reactivity studies

Reaction of $ZnBr_2$ with [Li][tBuPhBPin] (1a)

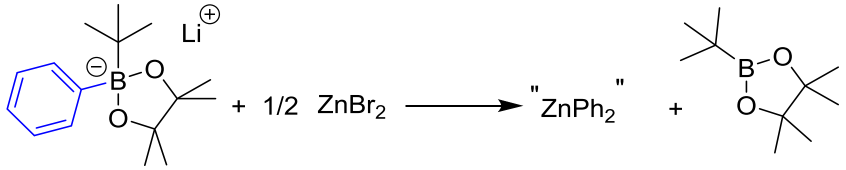

An oven dried J Young's NMR tube equipped with a DMSO-d_6 capillary insert was loaded with [Li][tBuPhBPin] (19 mg, 0.07 mmol) 2-MeTHF (0.7ml). The sample was analysed by ^{1}H NMR spectroscopy prior to addition of zinc bromide (8 mg, 0.035 mmol) and sonication for 30s. The sample was analysed by ^{11}B NMR spectroscopy showing complete consumption of the borate starting material and formation of tBuBPin.

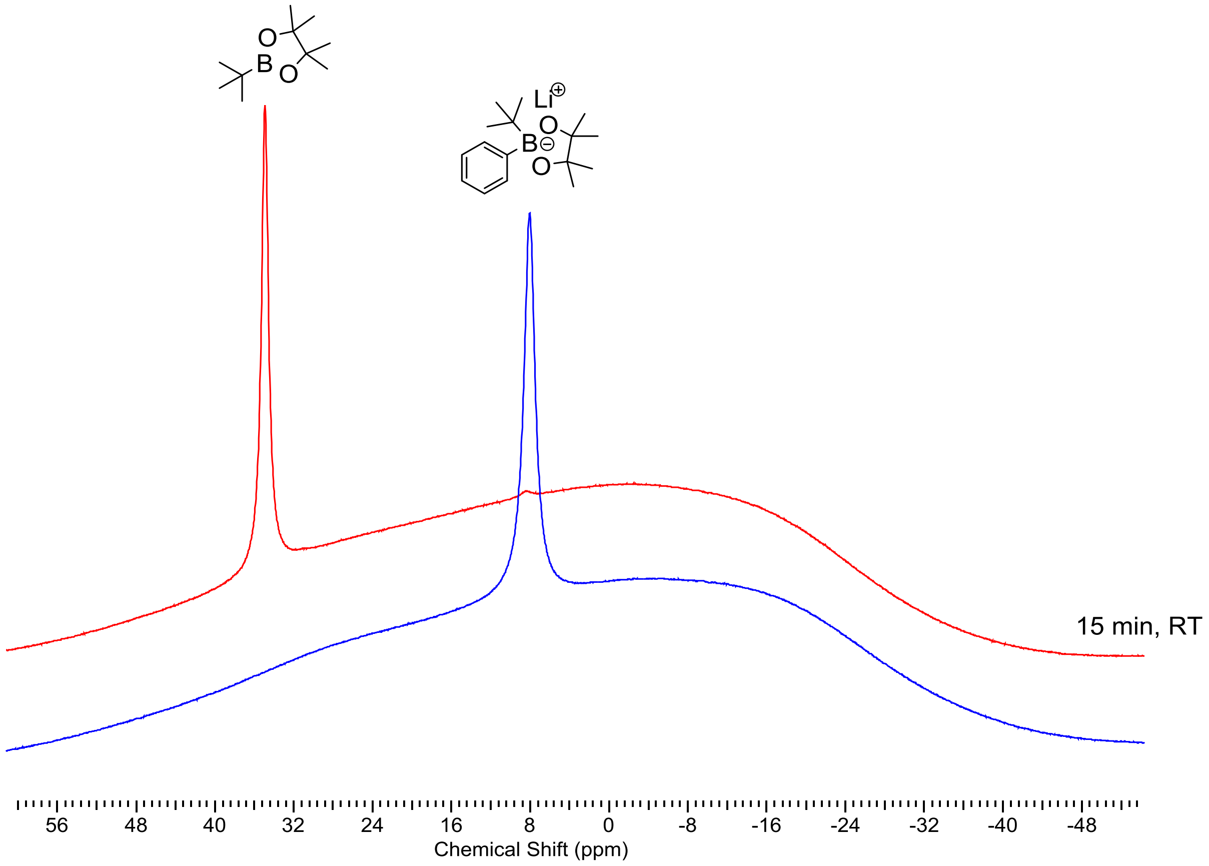

Figure S45: ^{11}B NMR spectrum of the reaction between $ZnBr_2$ and 1a

Reaction of $ZnPh_2$ with [Li][$^tBuPhBPin$] (1a)

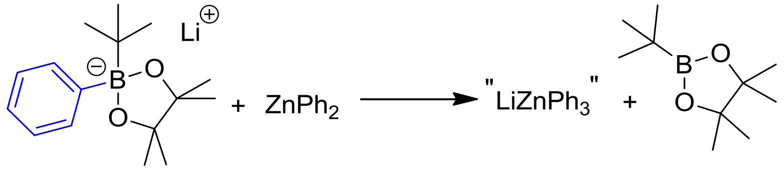

An oven dried J Young's NMR tube equipped with a DMSO-d_6 capillary insert was loaded with [Li][^{t}BuPhBPin] (86 mg, 0.32 mmol, 2 eq.), mesitylene (22.25 µl, 0.16 mmol as internal standard) and *protio*-2MeTHF (0.7ml). The sample was analysed by ^{1}H NMR spectroscopy and diphenyl zinc (35 mg, 0.16 mmol, 2 eq.) was added and the sample heated to 60 °C for 90 minutes. The sample was analysed by ^{1}H and ^{11}B NMR spectroscopy showing transfer of 1 phenyl equivalent. The sample was then heated at 60 °C for a further 16 hours. Analysis by NMR spectroscopy showed that 1 equivalent of the neutral ^{t}BuBPin had been formed by integration vs. the mesitylene standard, suggesting formation of $LiZnPh_3$ but not Li_2ZnPh_4.

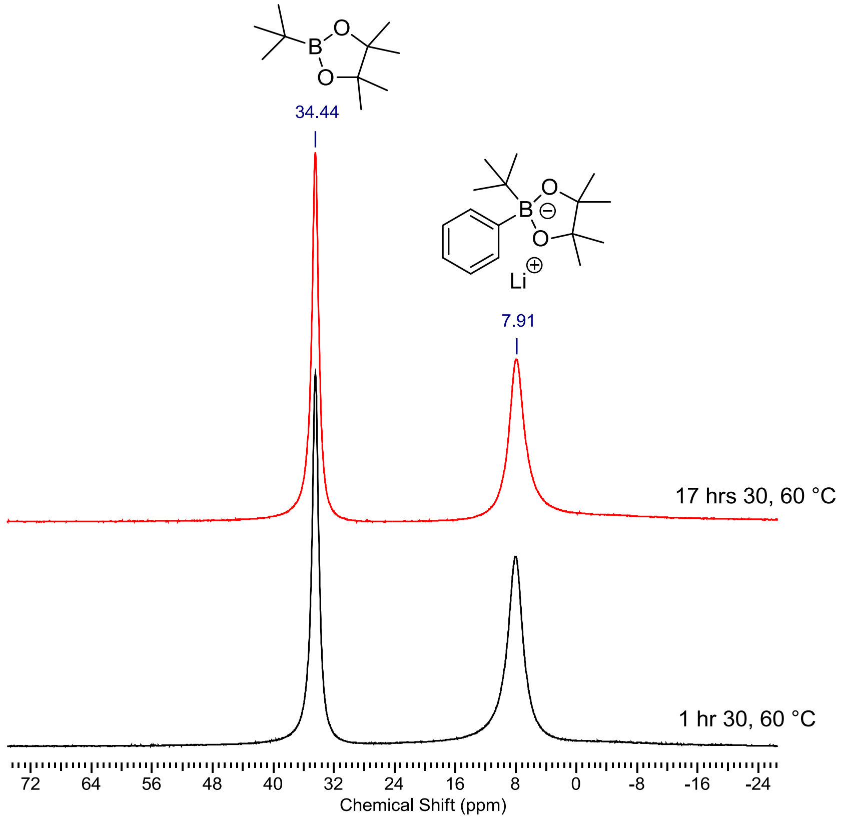

Figure S46: ^{11}B NMR spectrum of the reaction of $ZnPh_2$ with 2 eq. 1a

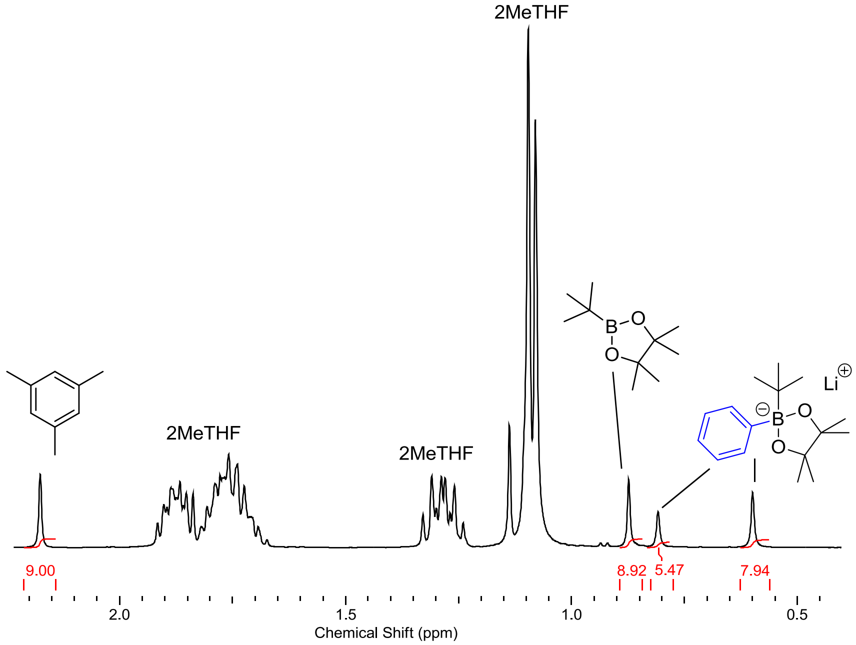

Figure S47: ^{1}H NMR spectrum of the reaction mixture after 18 hours heating to 60ºC. N.B. neutral ^{t}ButylBPin resonance indicated is *tert*-butyl 9H resonance

Interaction of $ZnPh_2$ with LiBr

An oven dried J Young's NMR tube equipped with a DMSO-d_6 capillary insert was loaded with $ZnPh_2$ (55mg, 0.25 mmol), and 2-MeTHF (700 µl). The ^{1}H NMR spectrum was measured. PhLi (22mg, 0.25 mmol) was added and the mix was sonicated for 40s. The mixture was analysed by ^{1}H and ^{7}Li NMR spectroscopy. A further equivalent of LiBr (22mg, 0.25 mmol total 0.5 mmol) was added, a further small shift in the ^{1}H NMR aryl resonances was observed. At this point a small amount of white solid could be observed.

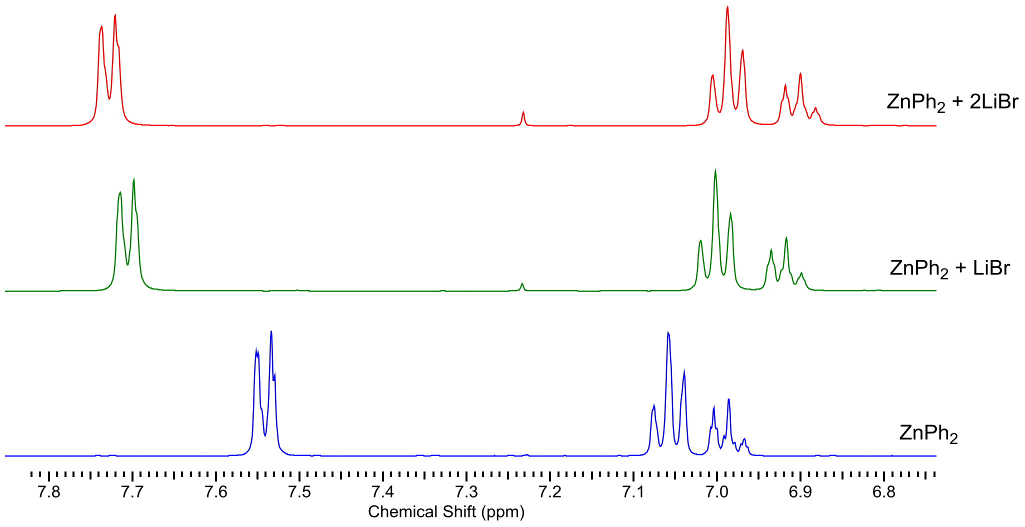

Figure S48: Aryl region of the ^{1}H NMR spectrum in 2MeTHF of $ZnPh_2$ and increasing equivalents of lithium bromide

Reaction of $ZnPh_2$ with 1a in the presence of LiBr

An oven dried J Young's NMR tube equipped with a DMSO-d_6 capillary insert was loaded with $ZnPh_2$ (27 mgs, 1eq.), LiBr (1 or 2 eq. 11mgs or 22mgs) and 2MeTHF was added (0.7 ml). [Li][^{t}BuPhBPin] (33 mgs, 1eq.) was added and the solution was heated to 60 °C for 30 minutes prior to analysis by ^{1}H and ^{11}B{^{1}H} NMR spectroscopy, after standing at room temperature for 30 minutes, the solution was heated to 60°C and analysed by ^{1}H and ^{11}B{^{1}H} NMR spectroscopy after a further 30 minutes and after 18 hours at 60°C.

Table S 6: Conversion based on ^{1}H NMR specrtoscopy

Equivalents LiBr	Time at 60 °C	Conversion of 1a to tBuBPin
1	1 hour	43%
	18 hours	87%
2	1 hour	37%
	18 hours	87%

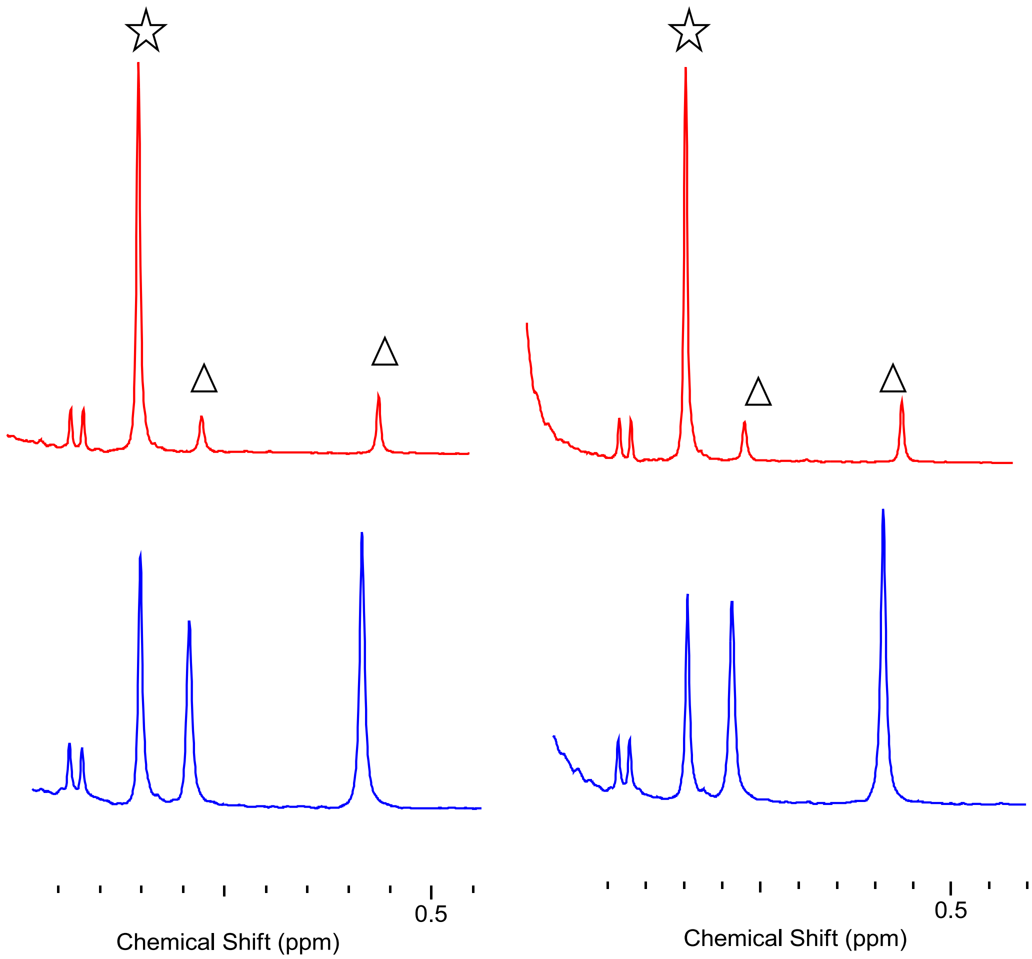

Figure S49: ^{1}H NMR spectra of of the reactions of 1a with $ZnPh_2$ in the presence of (left) 1 eq. LiBr and (right) 2 eq. LiBr. Bottom, after 1 hour at°60 C; top, after 18 hours at 60°C. Stars: tBuBPin; triangles, 1a

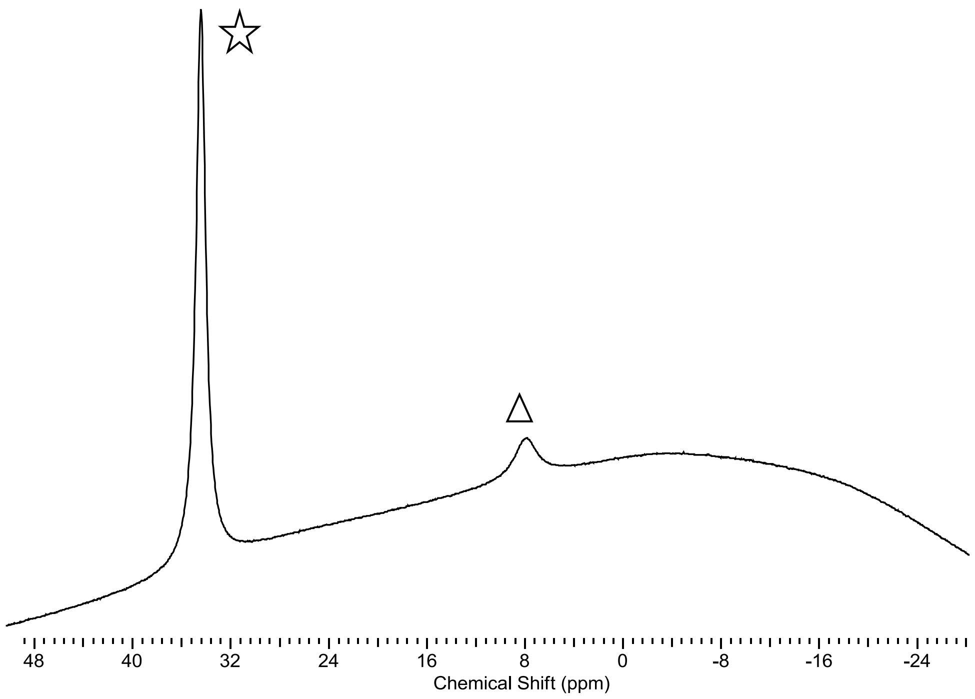

Figure S50: ^{11}B NMR spectrum of the reaction of 1a with $ZnPh_2$ in the presence of 2 eq. LiBr after heating to 60°C for 18 hours. Star: ^{t}BuBPin; Triangle: 1a.

Synthesis of Phenyllithium

Solvent free phenyllithium was synthesised according to a modified literature procedure[16]. Bromobenzene (1.4ml, 13.2 mmol) was dissolved in hexane and cooled to -80 °C, followed by dropwise addition of *n*-butyllithium (1.6M/hexanes, 8.2ml, 13.1 mmol) and allowed to warm slowly to room temperature. After stirring for 48 hours, a white precipitate had formed and was isolated by filtration and washed with hexane. ^{1}H NMR data matched reported literature values. [17]

Synthesis and Reaction of Li_2ZnPh_4 with 4-fluorobenzyl bromide

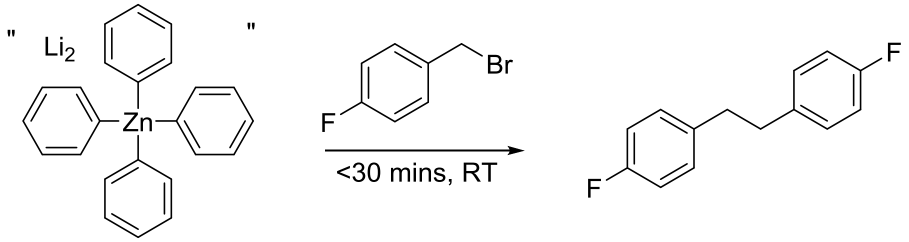

An oven dried J Young's NMR tube equipped with a DMSO-d_6 capillary insert was loaded with PhLi (26 mg, 0.32 mmol), $ZnPh_2$ (36 mg, 0.16 mmol), and 2-MeTHF (700 µl). The mixture was analysed by ^{1}H, ^{7}Li and ^{13}C{^{1}H} NMR spectroscopy which showed one set of resonances consistent with formation of Li_2ZnPh_4 (based on the ipso ^{13}C resonance and comparision to the work of Hevia et al.)[17].. To this mixture 4-fluorobenzyl bromide was added (20 µl, 0.16 mmol). After 20 minutes at room temperature analysis by ^{19}F NMR spectroscopy revealed complete consumption of the starting material. Analysis by GCMS revealed the presence of only 1,2-bis(4-fluorophenyl)ethane.

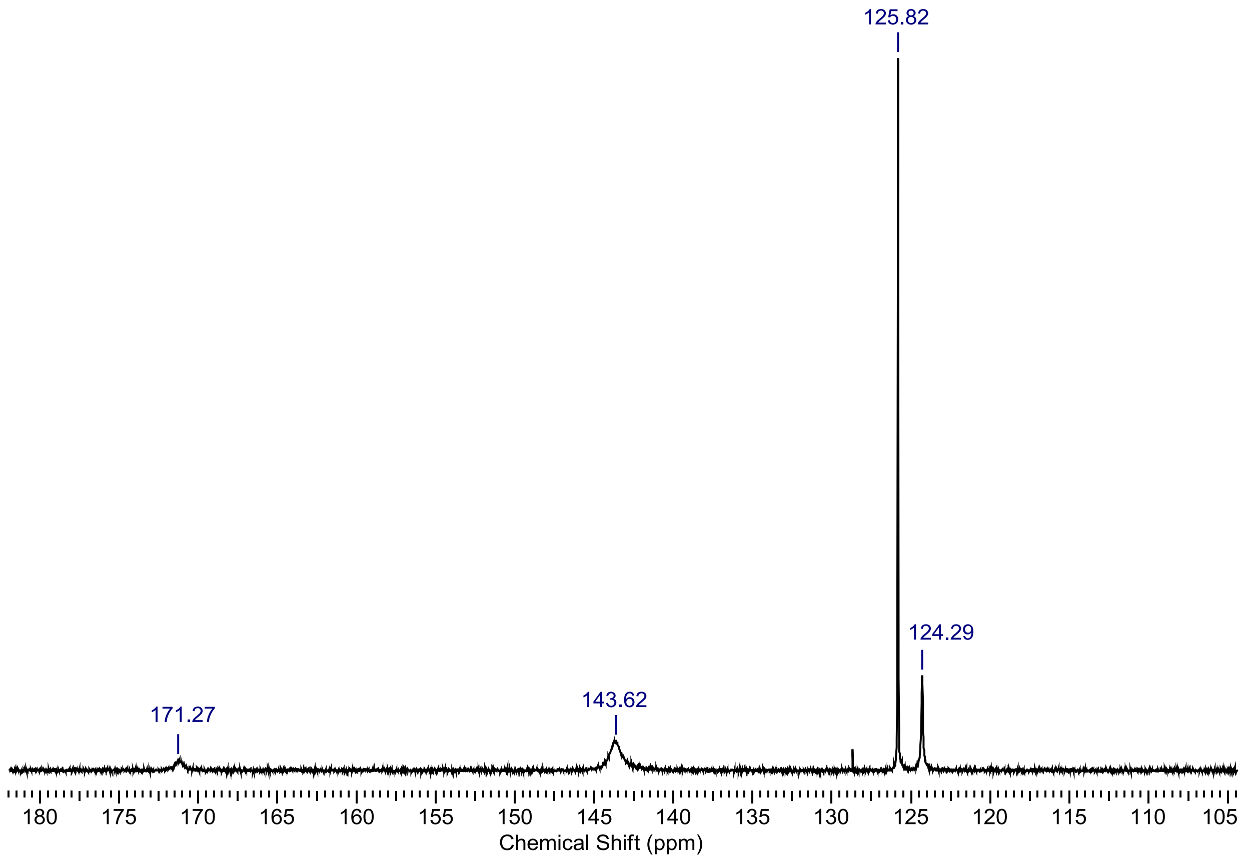

Figure S51: Aryl region of the ^{13}C{^{1}H} NMR spectrum of the reaction of $ZnPh_2$ with 2 equivalents of LiPh in 2-MeTHF before addition of the electrophile.

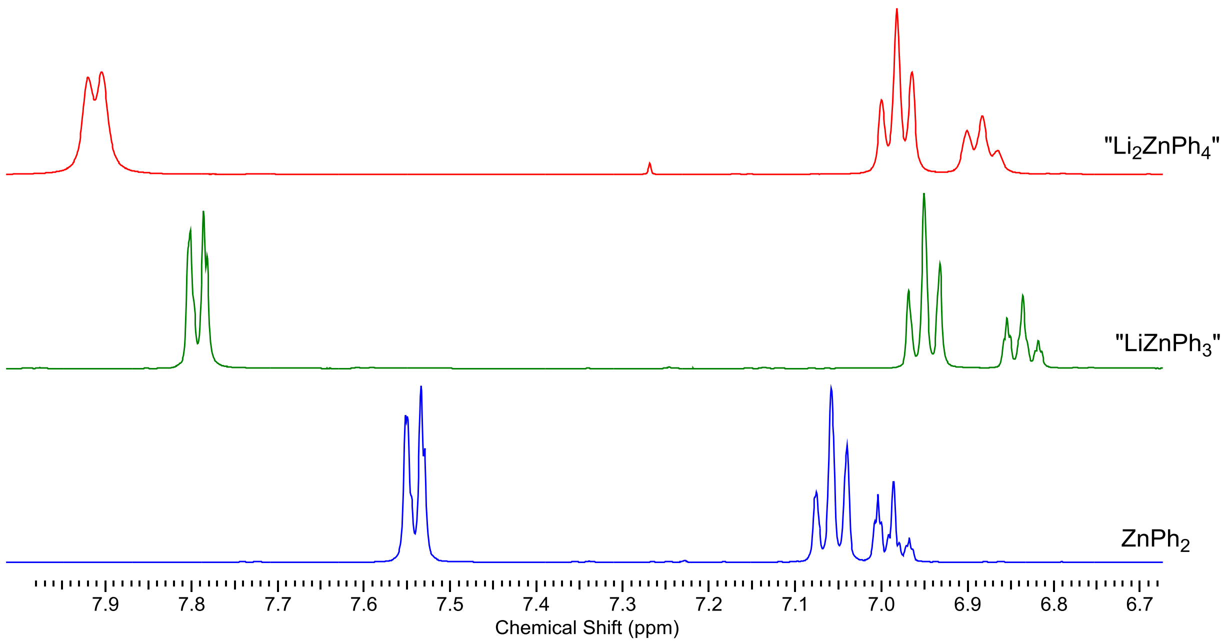

Figure S52: Aryl region of the ^{1}H NMR spectrum in 2MeTHF of $ZnPh_2$ following the addition of 0 (bottom), 1 (middle), and 2 (top) equivalents of LiPh

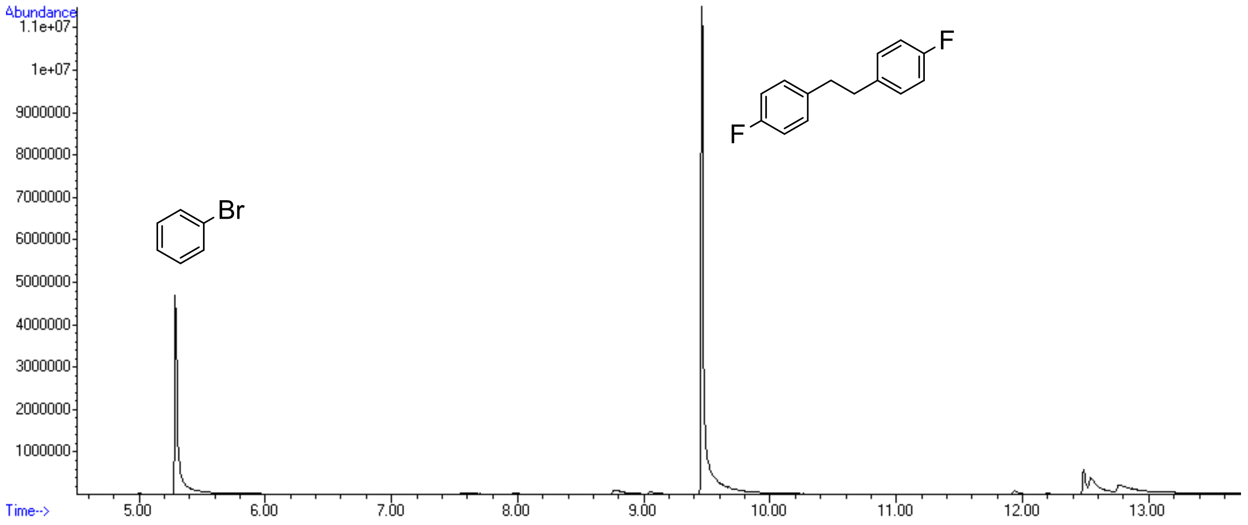

Figure S53: GCMS chromatogram of the reaction of Li_2ZnPh_4 with 4-fluorobenzyl bromide

Interaction of $LiZnPh_3$ with LiBr

An oven dried J Young's NMR tube equipped with a DMSO-*d*$_6$ capillary insert was loaded with $ZnPh_2$ (55mg, 0.25 mmol), Phenyllithium (21mg, 0.25 mmol) and 2-MeTHF (700 µl). Analysis by ^1H NMR showed formation of $LiZnPh_3$. Then LiBr (22mg, 0.25 mmol) was added, and the mixture sonicated for 40s. Analysis by ^1H NMR spectroscopy showed a small change in the aryl resonances. A further equivalent of LiBr (22mg, 0.25 mmol, total 0.5 mmol) was added, and analysis by ^1H NMR spectroscopy showed no further interaction. At this stage the solution was not completely homogeneous.

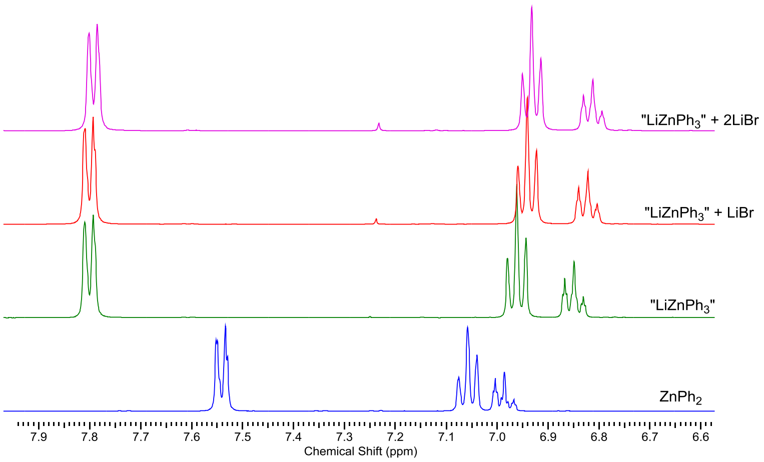

Figure S54: Aryl region of the ^1H NMR spectrum in 2MeTHF of $ZnPh_2$ (bottom), on addition of LiPh (2nd), LiPh + LiBr (3rd) and LiPh + 2 equivalents of LiBr (top)

Synthesis and Reaction of ‘$LiZnPh_3$’ with 4-fluorobenzyl bromide

An oven dried J Young’s NMR tube equipped with a DMSO-d_6 capillary insert was loaded with diphenylzinc (26 mg, 0.12 mmol, 1 eq.), phenyllithium (10 mg, 0.12 mmol, 1 eq.), and 2-MeTHF (600 µl). The mixture was sonicated for 1 minute to aid dissolution of the solids, and the solution was analysed by ^{1}H and $^{13}C\{^{1}H\}$ NMR spectroscopy, which showed formation of $LiZnPh_3$. After standing overnight, 4-fluorobenzyl bromide (15 µl, 0.12 mmol, 1 eq.) was added and the mixture heated to 60°C for 90 minutes. The reaction was quenched with ethanol. The reaction was analysed by ^{19}F NMR spectroscopy before dilution with DCM, filtration through a small silica plug and analysis by GCMS which showed the formation of 1-benzyl 4-fluorobenzene (59%) and 1,2-bis(4-fluorophenyl)ethane (11%).

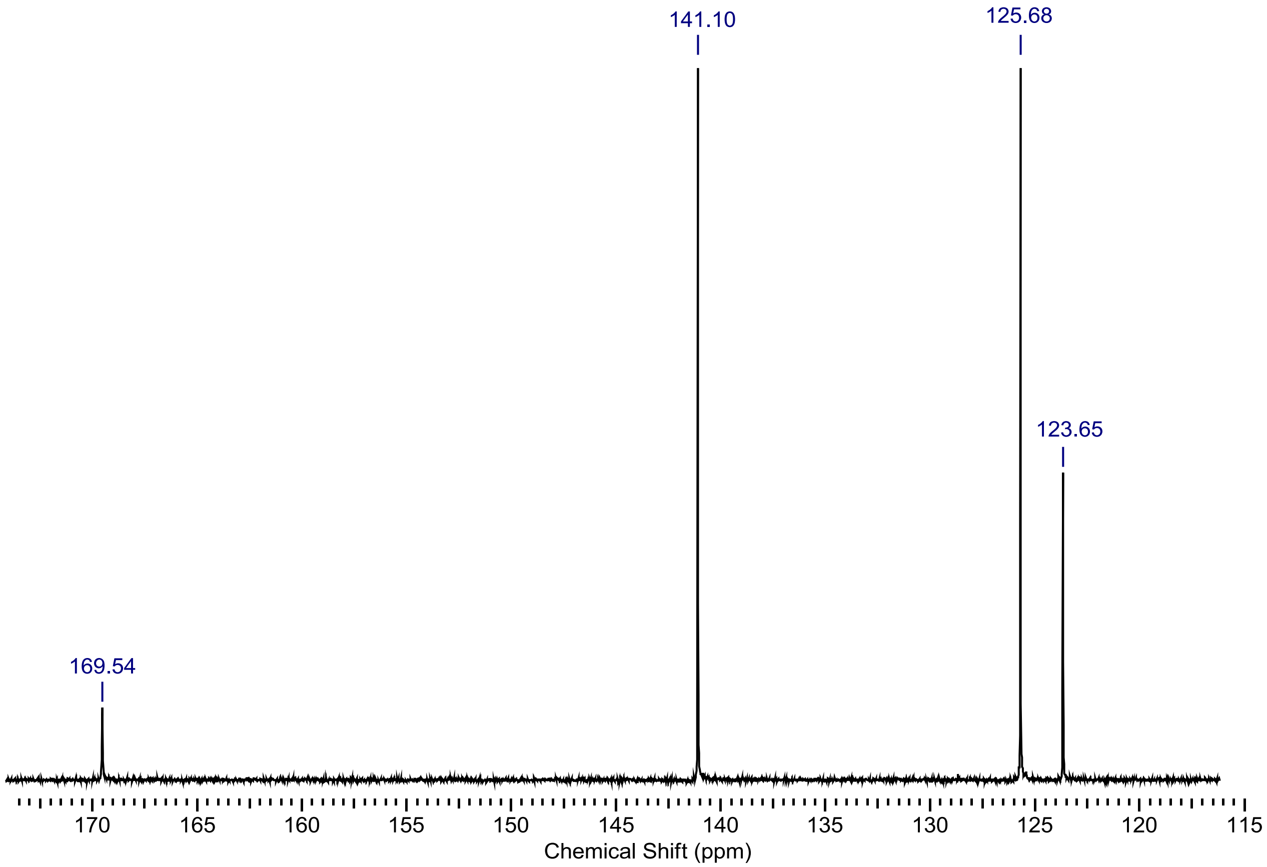

Figure S55: Aryl region of the $^{13}C\{^{1}H\}$ NMR spectrum of '$LiZnPh_3$' before addition of electrophile

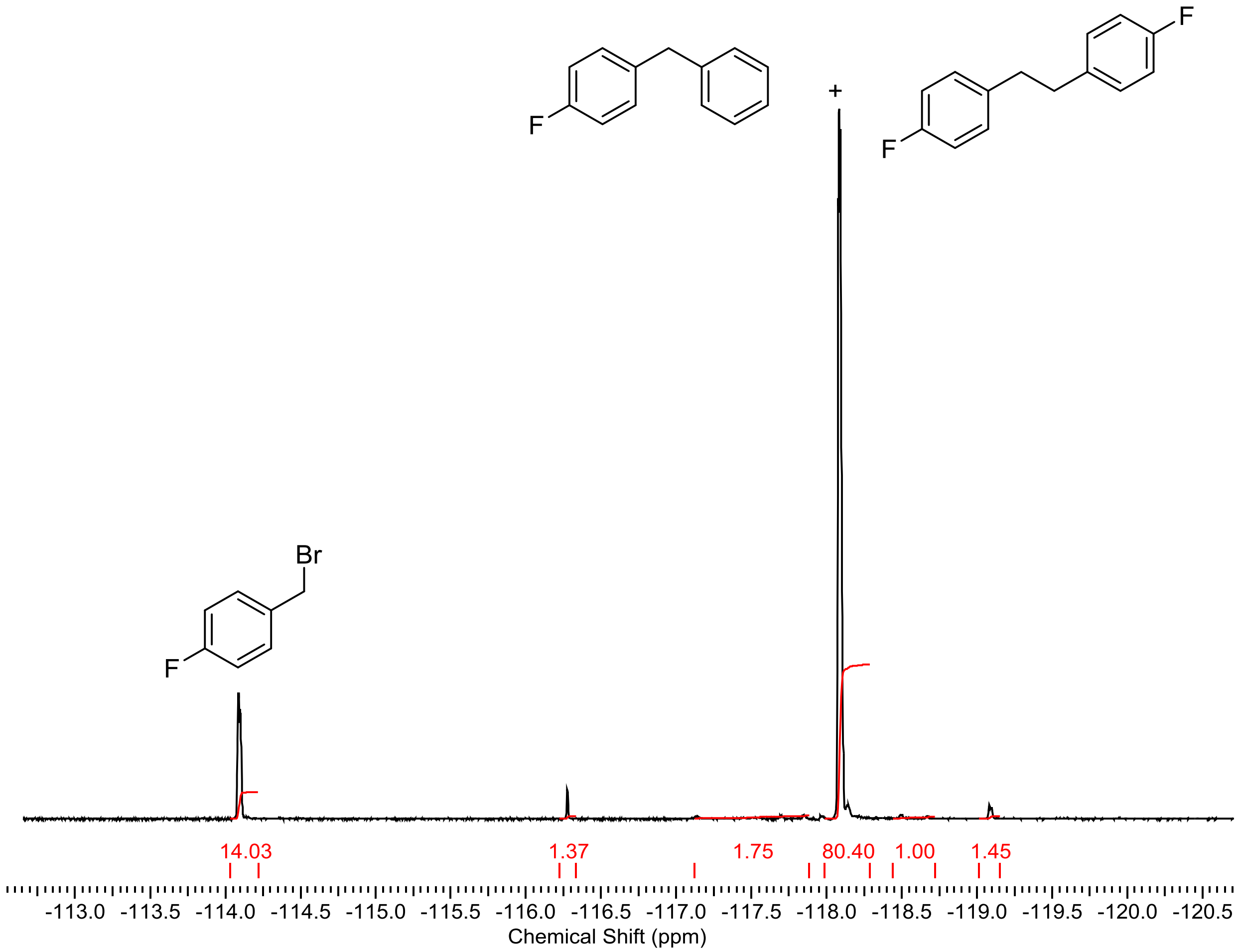

Figure S56: ^{19}F NMR spectrum of the reaction between $LiZnPh_3$ and 4-fluorobenzyl bromide

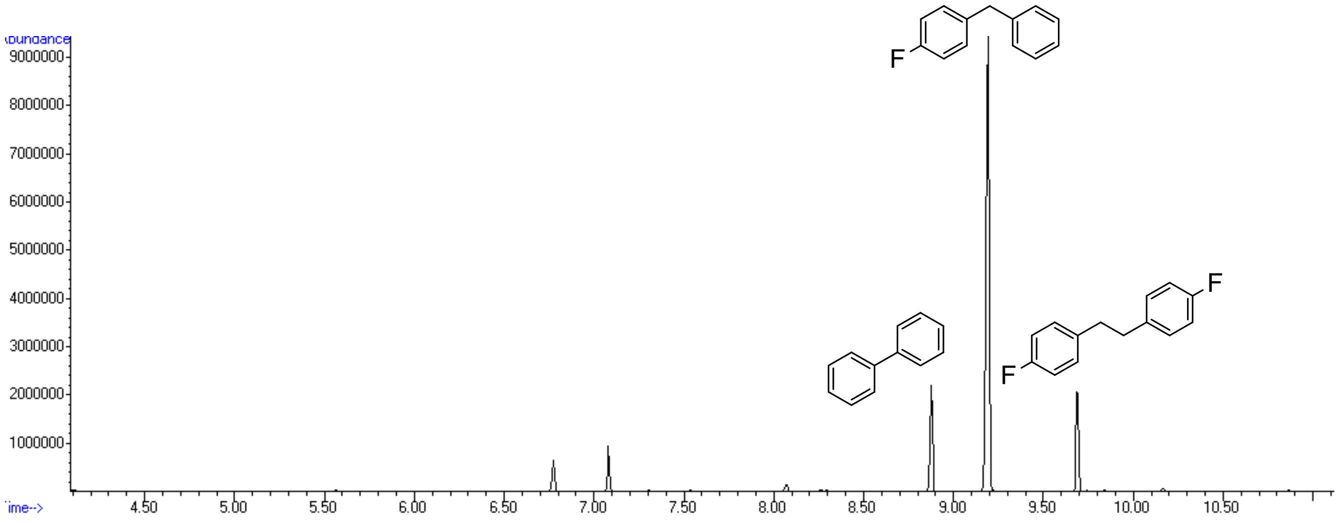

Figure S57: GCMS chromatogram of the reaction between '$LiZnPh_3$' and 4-fluorobenzyl bromide

Synthesis and Reaction of ‘$LiZnPh_3$’ with 4-fluorobenzyl bromide in the presence of LiBr

An oven dried J Young’s NMR tube equipped with a DMSO-d_6 capillary insert was loaded with diphenylzinc (26 mg, 0.12 mmol, 1 eq.), phenyllithium (10 mg, 0.12 mmol, 1 eq.), lithium bromide (21 mg, 0.24 mmol, 2 eq.) and 2-MeTHF (600 µl). The mixture was sonicated for 1 minute to aid dissolution of the solids, and the solution was analysed by ^{1}H and $^{13}C\{^{1}H\}$ NMR spectroscopy, which showed formation of $LiZnPh_3$. After standing overnight, 4-fluorobenzyl bromide (15 µl, 0.12 mmol, 1 eq.) was added and the mixture heated to 60°C for 90 minutes. The reaction was quenched with ethanol and fluorobenzene (1eq.) was added as an internal standard. The reaction was analysed by ^{19}F NMR spectroscopy which showed the formation of 1-benzyl 4-fluorobenzene (63%) and 1,2-bis(4-fluorophenyl)ethane (11%).

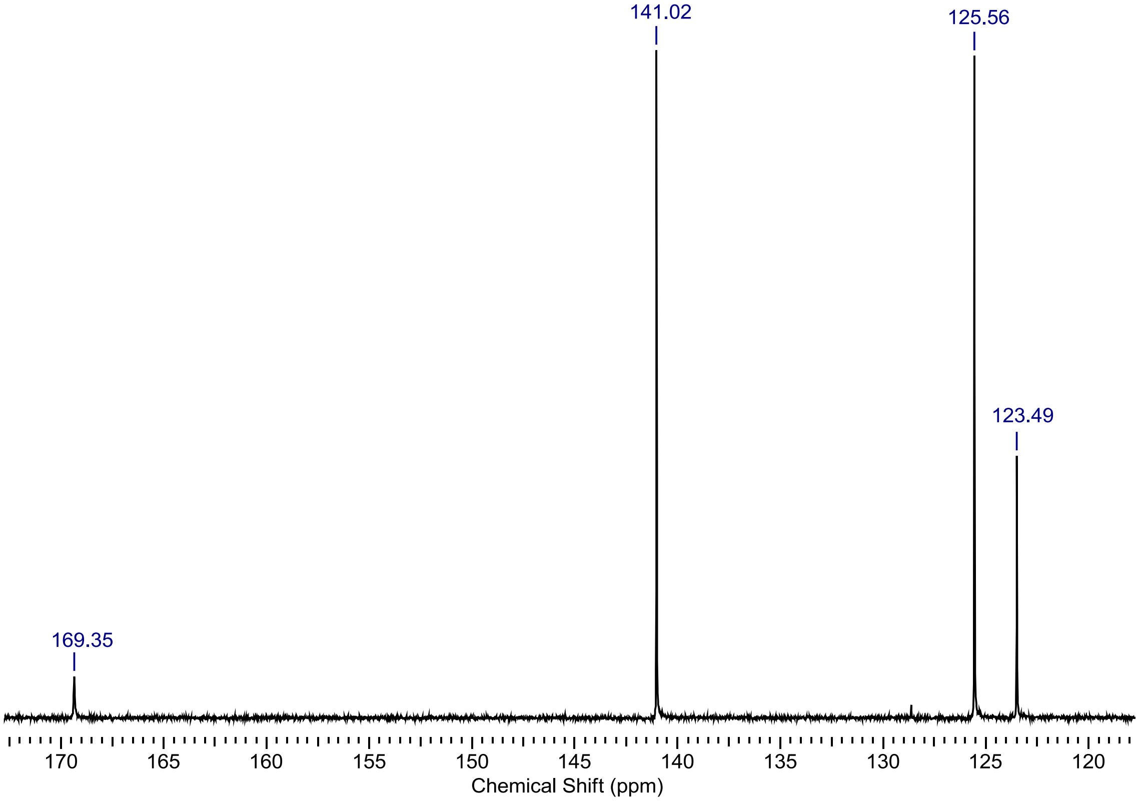

Figure S58: Aryl region of the $^{13}C\{^{1}H\}$ spectrum of '$LiZnPh_3$' in the presence of LiBr before addition of the electrophile

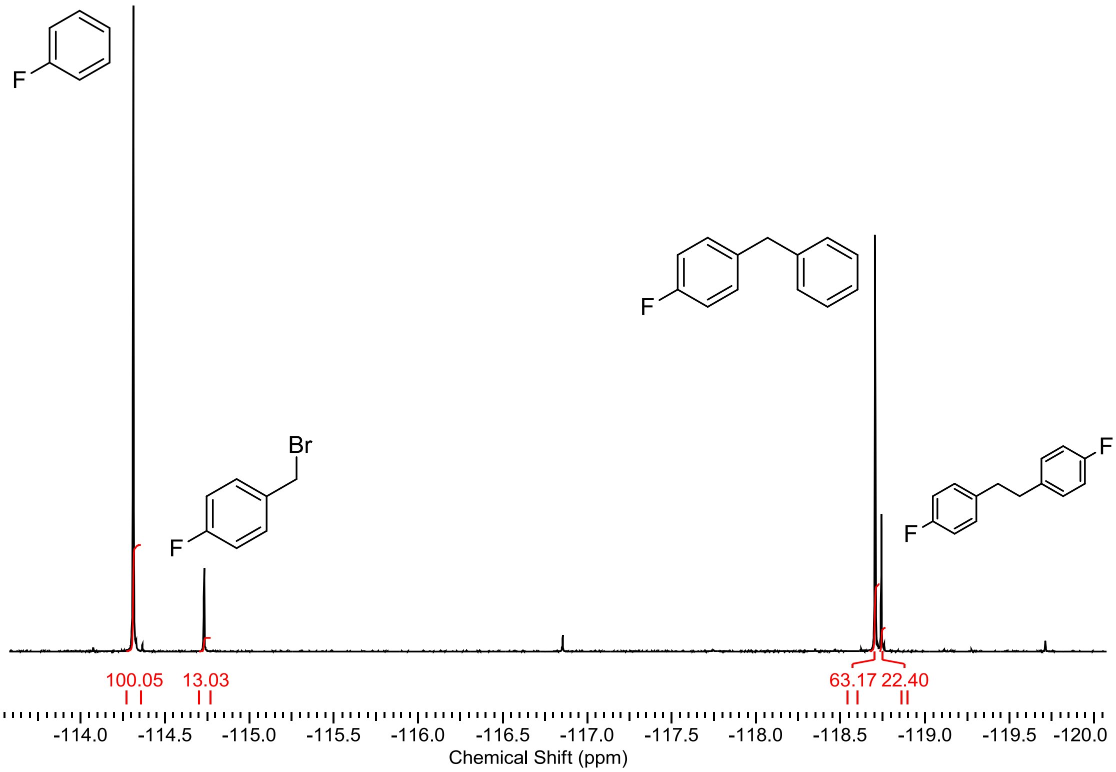

Figure S59: ^{19}F NMR spectrum of the reaction between 'LiZnPh$_3$' and 4-fluorobenzyl bromide in the presence of LiBr

Synthesis and Reaction of 'LiZnPh$_3$' with 4-fluorobenzyl bromide in the presence of ^{t}BuBPin

An oven dried J Young's NMR tube equipped with a benzene-d_6 capillary insert was loaded with diphenylzinc (26 mg, 0.12 mmol, 1 eq.), **1a** (32 mg, 0.12 mmol, 1 eq.), and 2-MeTHF (600 µl). The mixture was sonicated for 1 minute to aid dissolution of the solids, heated for 18 hours at 60°C and was analysed by ^{1}H, ^{13}C{^{1}H}, and ^{11}B NMR spectroscopy, which showed formation of LiZnPh$_3$ (based on the conversion of the majority of 1a to ^{t}BuBPin). Then 4-fluorobenzyl bromide (15 µl, 0.12 mmol, 1 eq.) was added and the mixture heated to 60°C for 90 minutes. The reaction was quenched with ethanol and analysed by ^{19}F NMR before dilution with DCM, filtration through a small silica plug and analysis by GCMS which showed the formation of 1-benzyl 4-fluorobenzene (69%) and 1,2-bis(4-fluorophenyl)ethane (9%).

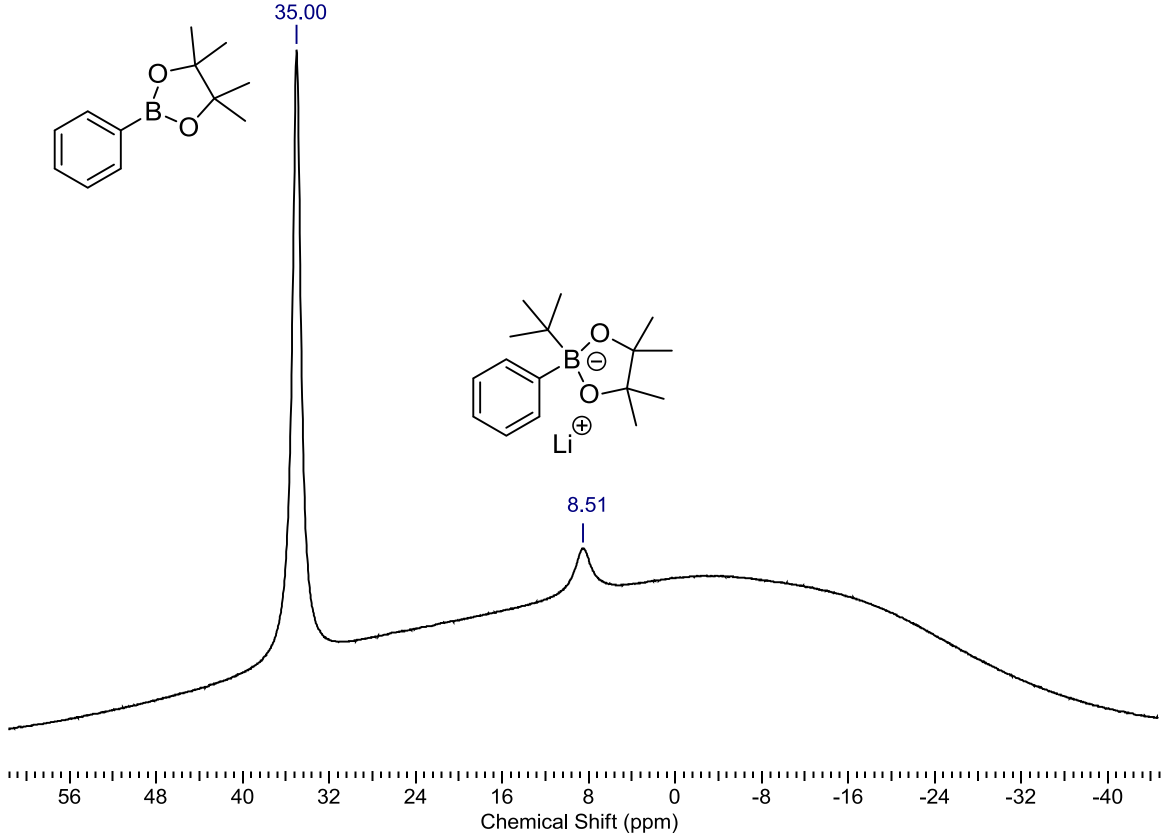

Figure S60: ^{11}B NMR spectrum of the reaction between ZnPh$_2$ and 1a after 18 hours at 60°C

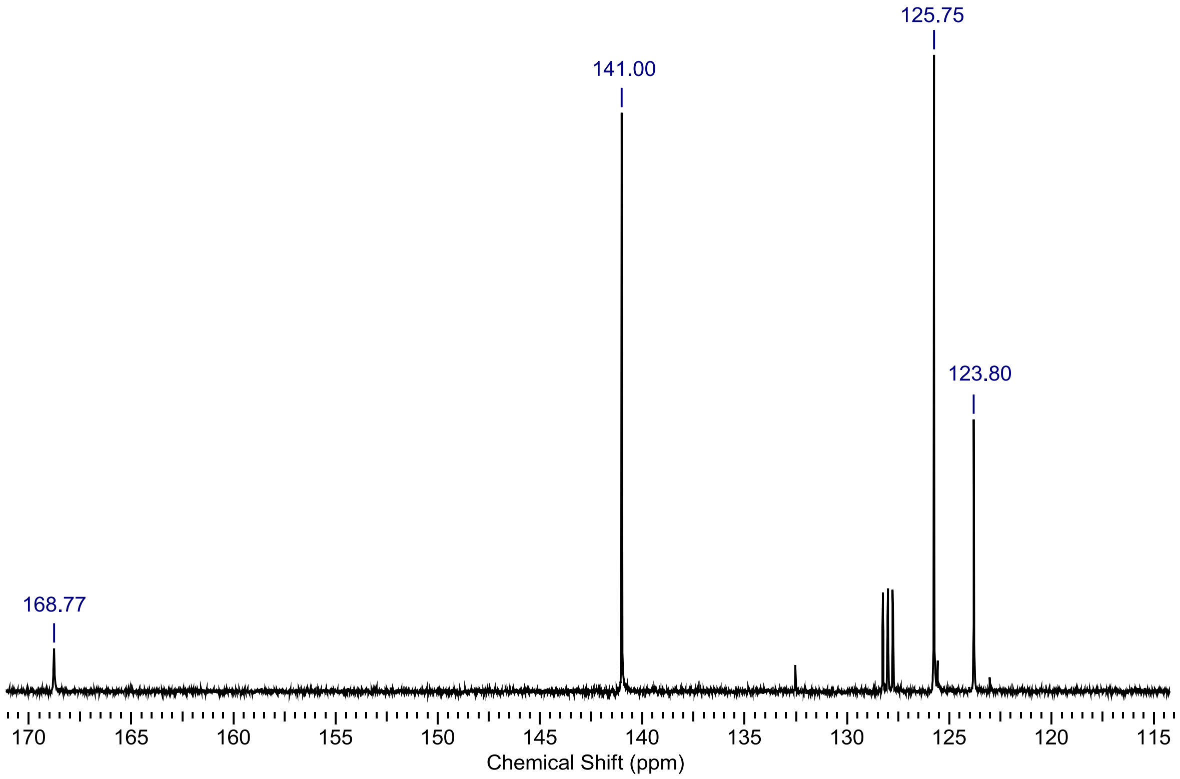

Figure S61: Aryl region of the $^{13}C\{^{1}H\}$ NMR spectrum of the 'LiZnPh$_3$' synthesised from 1a and ZnPh$_2$ prior to the addition of electrophile

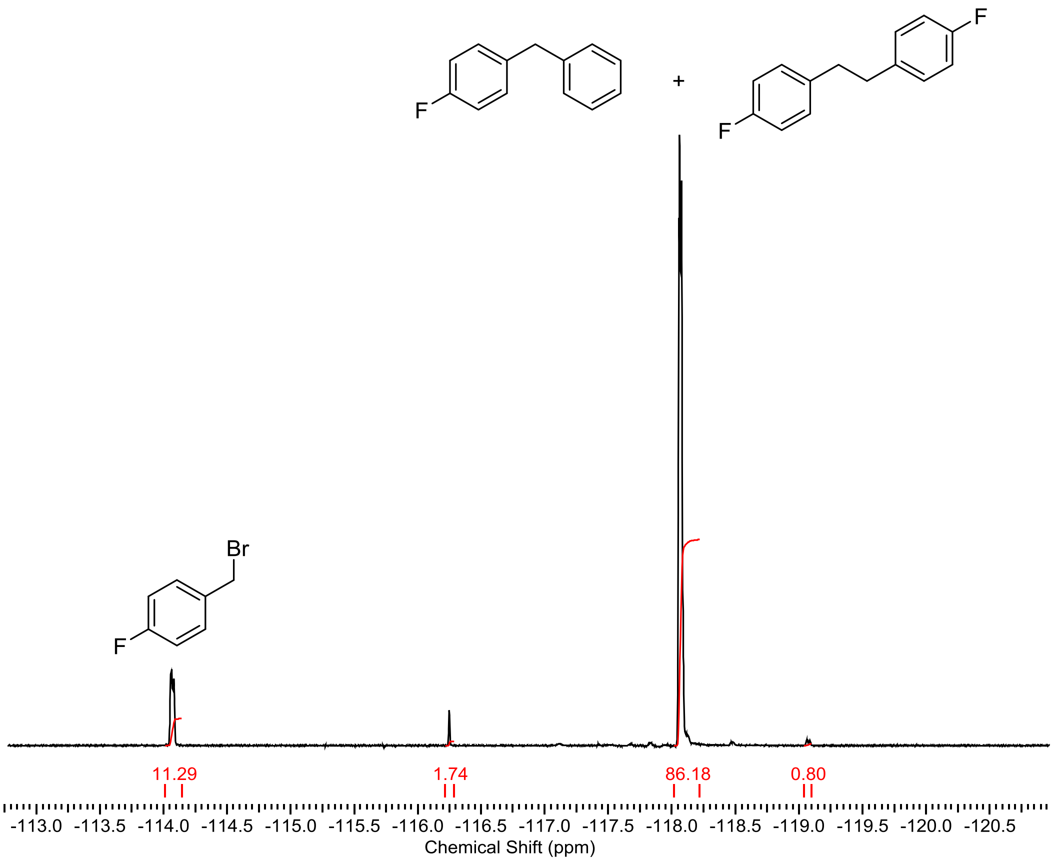

Figure S62: ^{19}F NMR spectrum of the reaction of 'LiZnPh$_3$' synthesised from 1a and ZnPh$_2$ with 4-fluorobenzyl bromide

Synthesis and Reaction of 'LiZnPh$_3$' with 4-fluorobenzyl bromide in the presence of LiBr at a catalytically relevant concentration (of 4-fluorobenzyl bromide)

An oven dried J Young's NMR tube equipped with a DMSO-d_6 capillary insert was loaded with diphenylzinc (13 mg, 0.06 mmol, 1 eq.), phenyllithium (5 mg, 0.06 mmol, 1 eq.), lithium bromide (10mg, 0.12 mmol, 2 eq.), and 2-MeTHF (520 µl). The mixture was sonicated for 1 minute to aid dissolution of the solids, and the solution was analysed by ^{1}H NMR spectroscopy. After standing overnight, 4-fluorobenzyl bromide (7.5 µl, 0.06 mmol, 1 eq., equivalent to the concentration that would be present at the start of the standard catalysis run) was added and the mixture heated to 60°C for 90 minutes. The reaction was quenched with ethanol and analysed by ^{19}F NMR before dilution with DCM, filtration through a small silica plug and analysis by GCMS which showed the formation of 1-benzyl 4-fluorobenzene (60%) and 1,2-bis(4-fluorophenyl)ethane (9%).

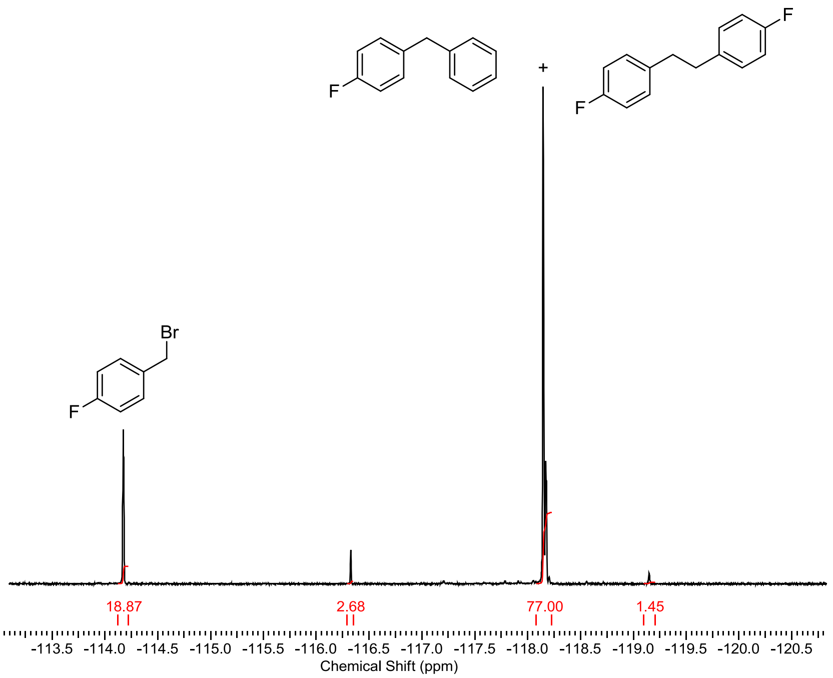

Figure S63: ^{19}F NMR spectrum of the reaction of LiZnPh$_3$ with 4-fluorobenzyl bromide in the presence of LiBr at lower concentration

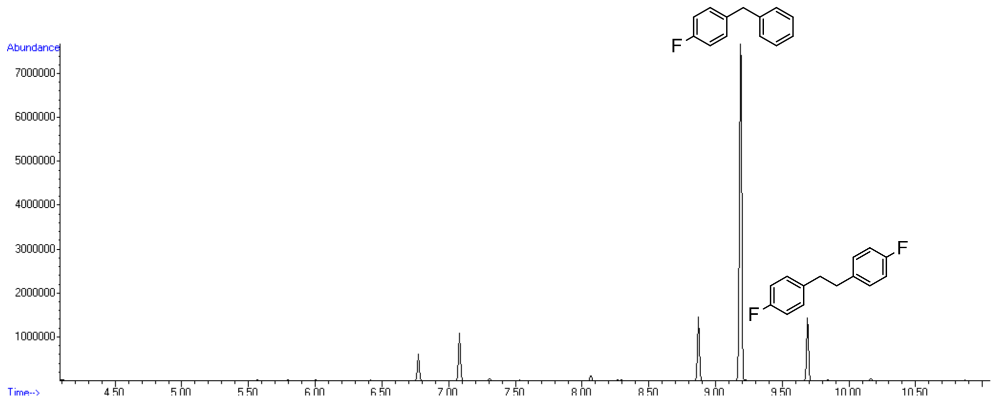

Figure S64: GCMS chromatogram of the reaction of LiZnPh$_3$ with 4-fluorobenzyl bromide in the presence of LiBr at lower concentration

Synthesis and Reaction of ‘$Li_2ZnPh_2Br_2$’ with 4-fluorobenzyl bromide

An oven dried J Young’s NMR tube equipped with a DMSO-d_6 capillary insert was loaded with diphenylzinc (26 mg, 0.12 mmol, 1 eq.), lithium bromide (21 mg, 0.24 mmol, 1 eq.), and 2-MeTHF (600 µl). The mixture was sonicated for 1 minute to aid dissolution of the solids, and the solution was analysed by ^{1}H and $^{13}C\{^{1}H\}$ NMR spectroscopy, which showed formation of some interaction of LiBr with $ZnPh_2$. After standing overnight, 4-fluorobenzyl bromide (15 µl, 0.12 mmol, 1 eq.) was added and the mixture heated to 60°C for 90 minutes. The reaction was quenched with ethanol and fluorobenzene (1 eq.) was added as an internal standard. The mixture was analysed by ^{19}F NMR spectroscopy which showed the formation of 1-benzyl 4-fluorobenzene (3%) and 1,2-bis(4-fluorophenyl)ethane (10%).

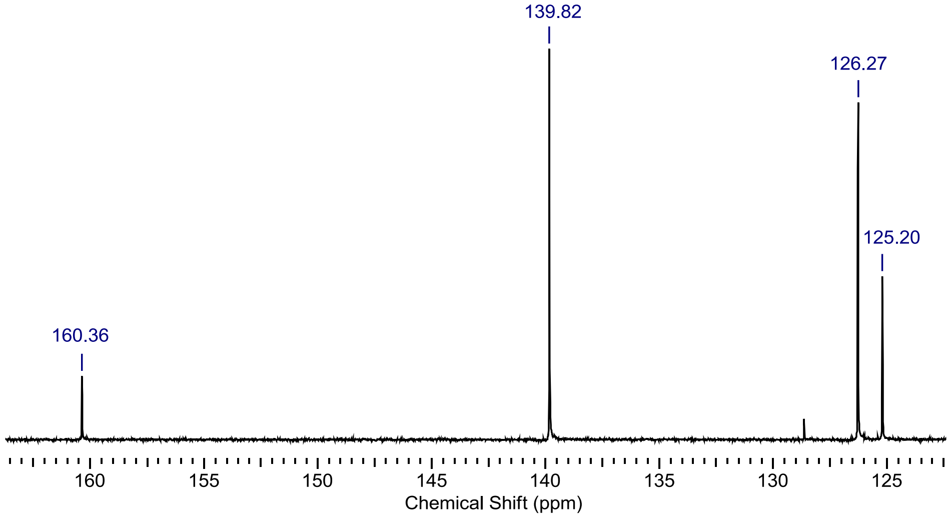

Figure S65: Aryl region of the $^{13}C\{^{1}H\}$ NMR spectrum of the zincate formed from $ZnPh_2$ and 2 eq. LiBr prior to addition of the electrophile

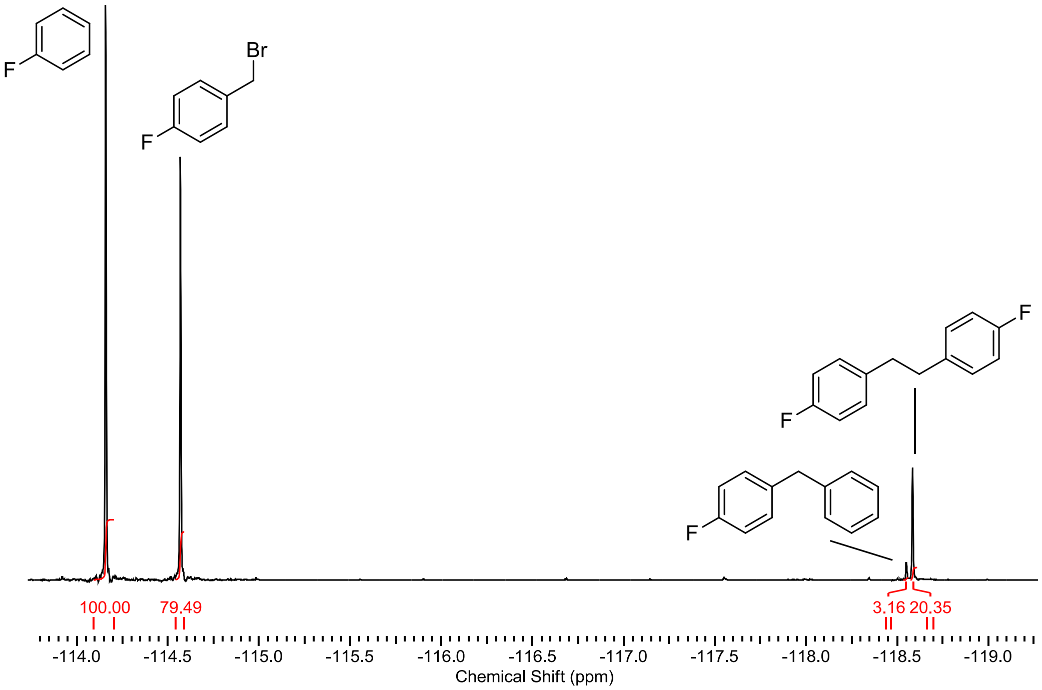

Figure S66: ^{19}F NMR spectrum of the reaction between '$Li_2ZnPh_2Br_2$' and 4-fluorobenzyl bromide

Synthesis and Reaction of ‘$Li_2ZnPhBr_3$’ with 4-fluorobenzyl bromide

An oven dried J Young’s NMR tube equipped with a DMSO-d_6 capillary insert was loaded with zinc bromide (27 mg, 0.12 mmol, 1 eq.), phenyllithium (10 mg, 0.12 mmol, 1 eq.), lithium bromide (10 mg, 0.12 mmol, 1 eq.) and 2-MeTHF (600 µl). The mixture was sonicated for 1 minute to aid dissolution of the solids, and the solution was analysed by ^{1}H and $^{13}C\{^{1}H\}$ NMR spectroscopy, which showed reaction of $ZnBr_2$ with PhLi. After standing overnight, 4-fluorobenzyl bromide (15 µl, 0.12 mmol, 1 eq.) was added and the mixture heated to 60°C for 90 minutes. The reaction was quenched with ethanol and analysed by ^{19}F NMR which showed no conversion of starting material.

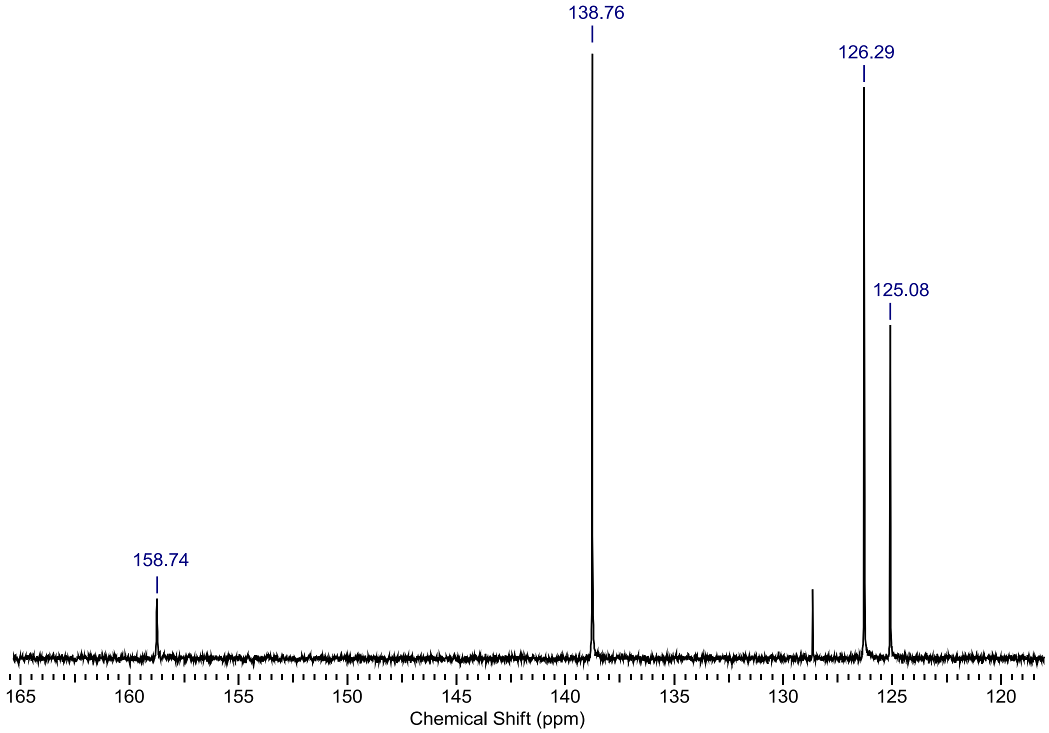

Figure S67: Aryl region of the $^{13}C\{^{1}H\}$ NMR spectrum of the bromide rich zincate prior to addition of the electrophile

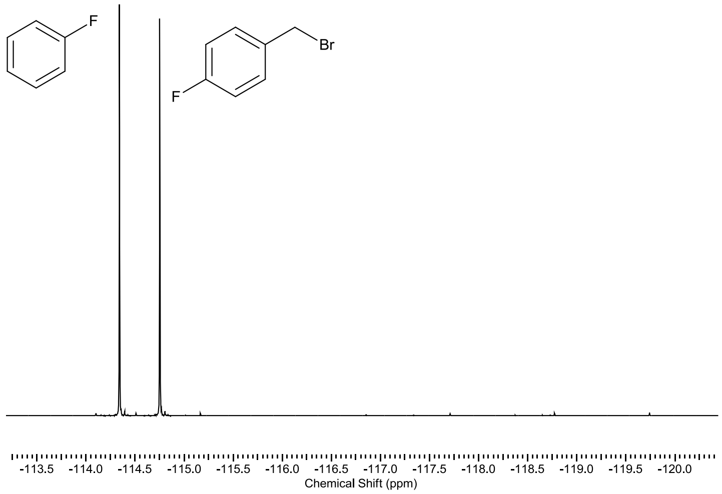

Figure S68: ^{19}F NMR spectrum of the reacion of '$Li_2ZnPhBr_3$' with 4-fluorobenzyl bromide (coupling products expected at -118.75 ppm are not present)

Reaction of $NaBPh_4$ with $ZnPh_2$

An oven dried J Young's NMR tube equipped with a DMSO-d_6 capillary insert was loaded with diphenyl zinc (35 mg, 0.16 mmol, 1 eq.), and sodium tetraphenylborate (55 mg, 0.16 mmol, 1 eq.) and 2-MeTHF (600 µl). The mixture was sonicated for 1 minute to aid dissolution of the solids, after standing at room temperature for 90 minutes, the mixture was analysed by ^{11}B NMR spectroscopy revealing no phenyl group transfer. The mixture was heated to 60°C and analysed by ^{11}B NMR spectroscopy after 3 hours and 41 hours, which showed the presence of only unreacted $NaBPh_4$ (no (2-MeTHF)-BPh_3 was observed).

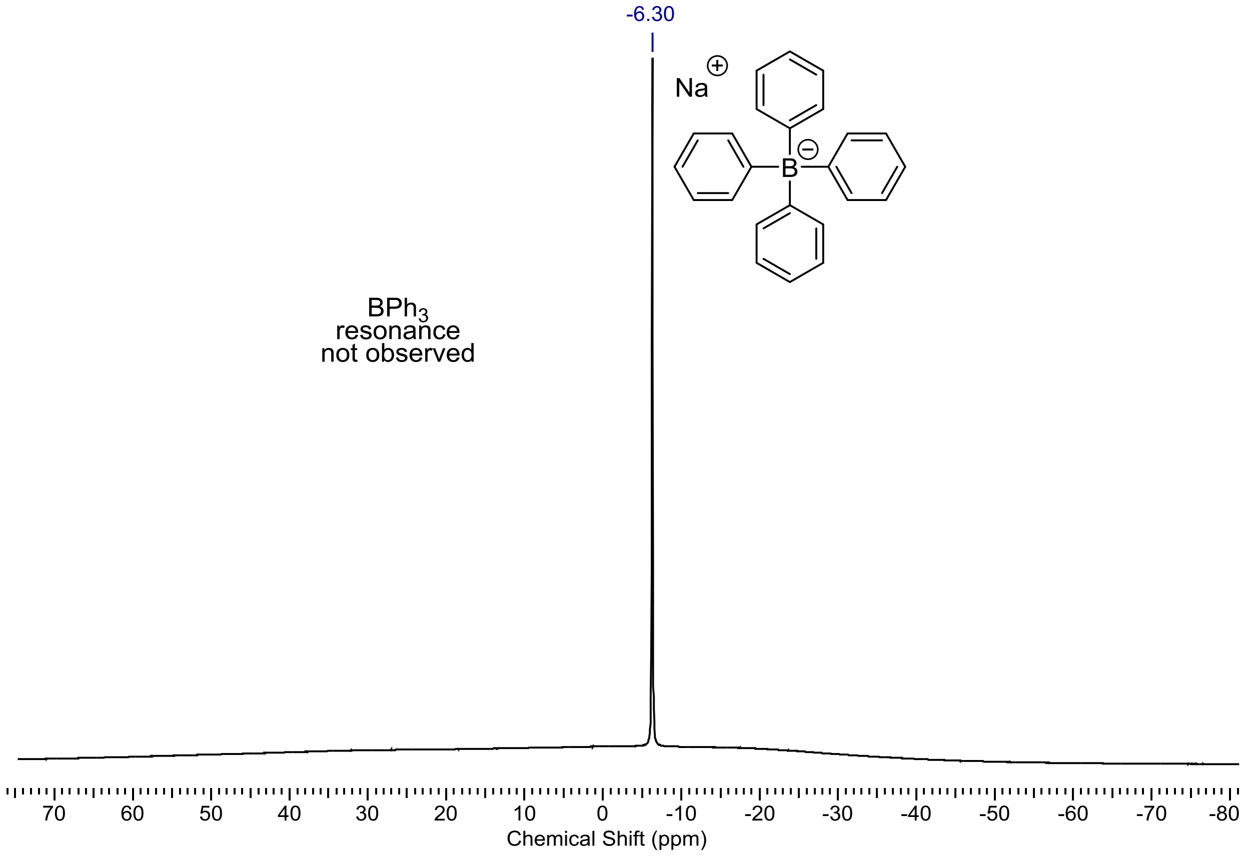

Figure S69: ^{11}B NMR spectrum of the attempted reaction of $NaBPh_4$ with $ZnPh_2$ after 41 hours at 60°C, showing no phenyl group transfer.

Triethylphosphine oxide Lewis acidity test

Reference values for Et_3PO in 2-MeTHF with relevant Lewis acidic species

In an oven dried J Young's NMR tube Et_3PO was dissolved in 2-MeTHF (600 µl), and analysed by ^{31}P NMR spectroscopy, which showed a chemical shift of 44.3 ppm. To this sample was added phenyl boronic acid pinacol ester (22.5 µl mg, 0.11 mmol), and the mixture was once again analysed by ^{31}P NMR spectroscopy, showing a chemical shift of 44.6 ppm (no change in chemical shift). To the same sample, lithium bromide was added (10 mg, 0.11 mmol), some precipitate was observed, and the sample was analysed by ^{31}P NMR spectroscopy, which showed a chemical shift of 58.3 ppm.

Et_3PO in representative reaction mixture

In an oven dried J Young's ampoule [Li][(^{t}Bu)(Ph)B(Pin)] **1a** (94 mg, 0.35 mmol) and $ZnBr_2$ (1 of a 2-MeTHF solution equating to 0.047 mmol) were dissolved in 2-MeTHF (2 ml). 4-fluorobenzyl bromide (29 µl, 0.23 mmol) was added. The reaction was heated to 60 °C for three hours before it was allowed to cool and triethylphosphine oxide (6.3 mg, 0.047 mmol) was added. The mixture was transferred to a J Young's NMR tube and analysed by ^{31}P NMR spectroscopy, which showed a chemical shift of 56.8 ppm for Et_3PO. This is compared to the value of 44.3 ppm obtained for free Et_3PO in 2-MeTHF.

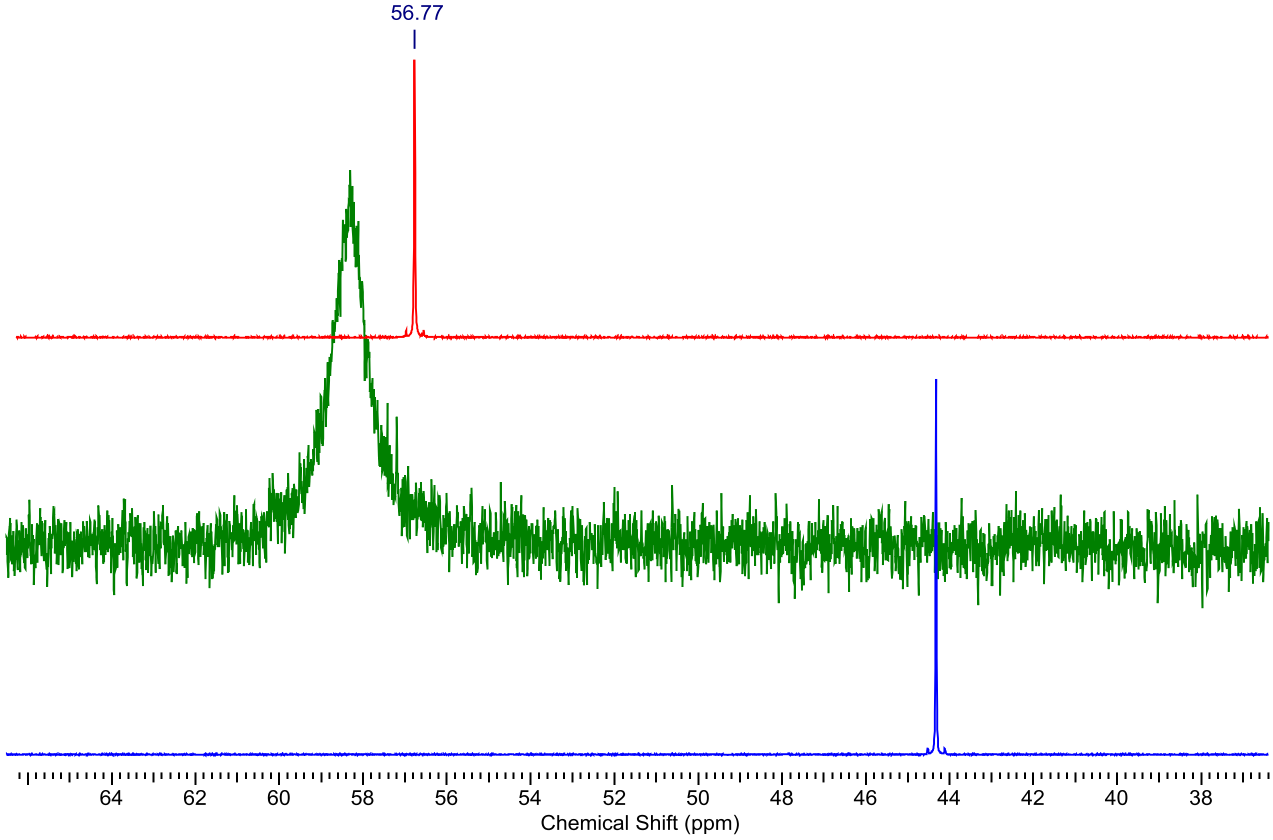

Figure S70: $^{31}P\{^1H\}$ NMR spectrum of triethylphosphine oxide (bottom), with LiBr (middle), and added to the catalytic mixture (after 3 h, see above, thus is in the presence of 1a/ $ZnBr_2$ / electrophile, top)

NMR Spectra of new compounds

1b

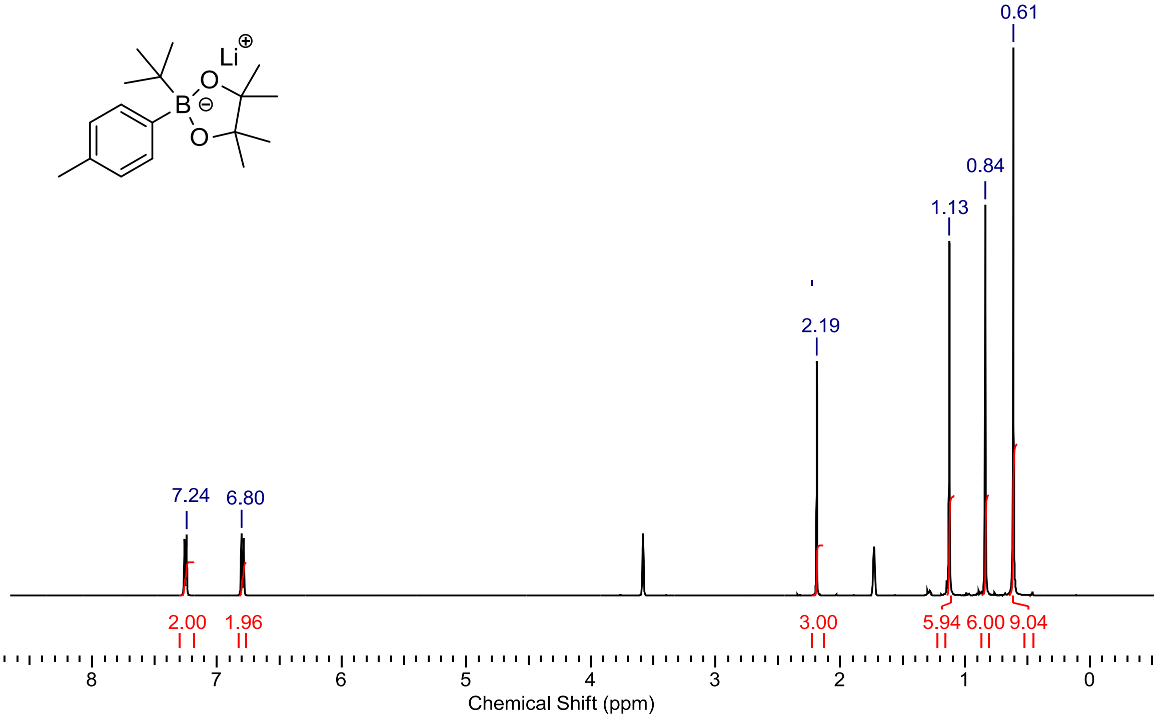

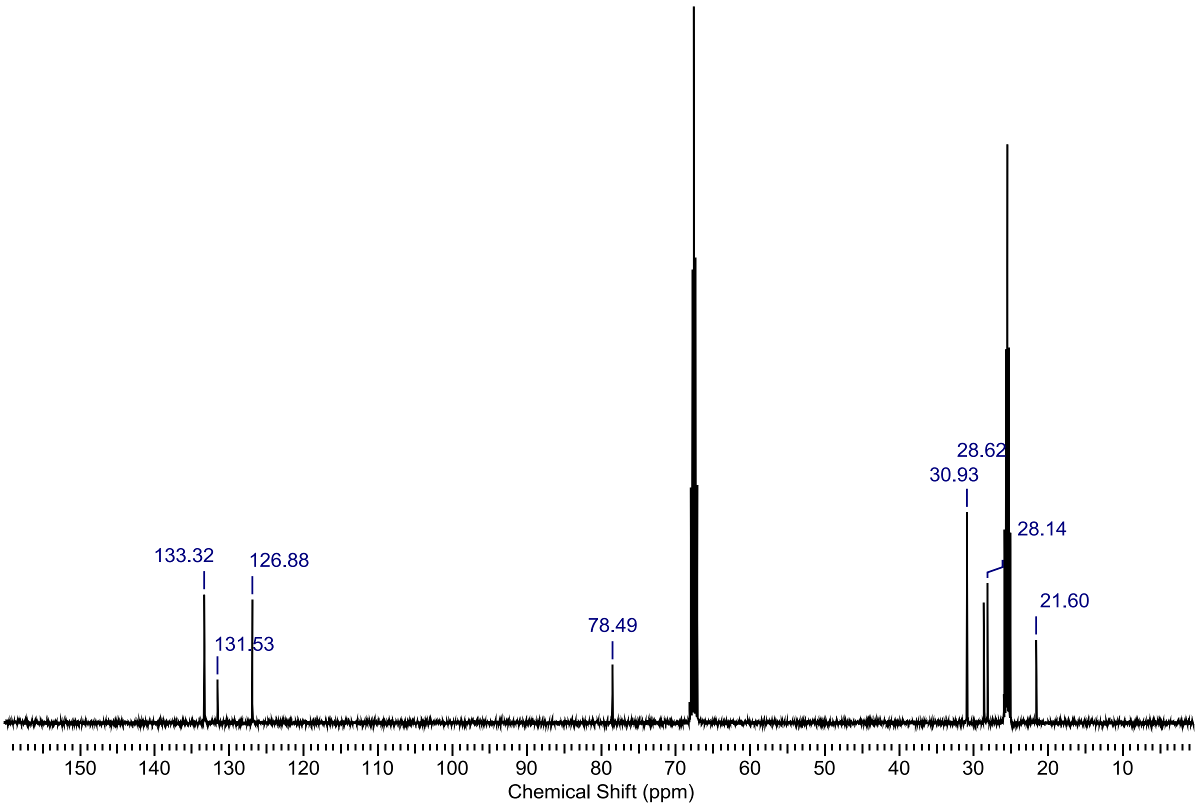

Figure S71: Top: ^{1}H NMR spectrum (d$_8$-THF) Bottom: ^{13}C{^{1}H} NMR spectrum (d$_8$THF)

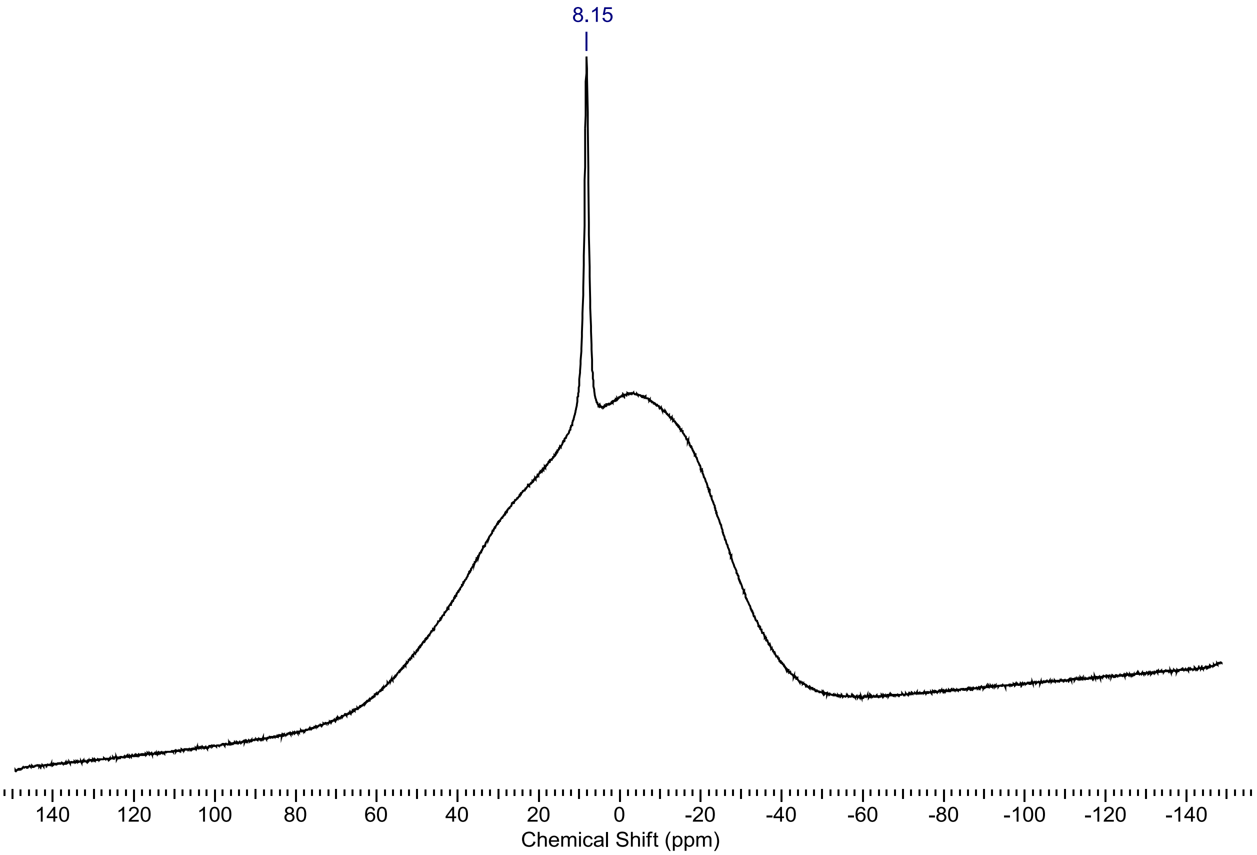

Figure S72: $^{11}B\{^{1}H\}$ NMR spectrum (d_8-THF, 128 MHz)

1c

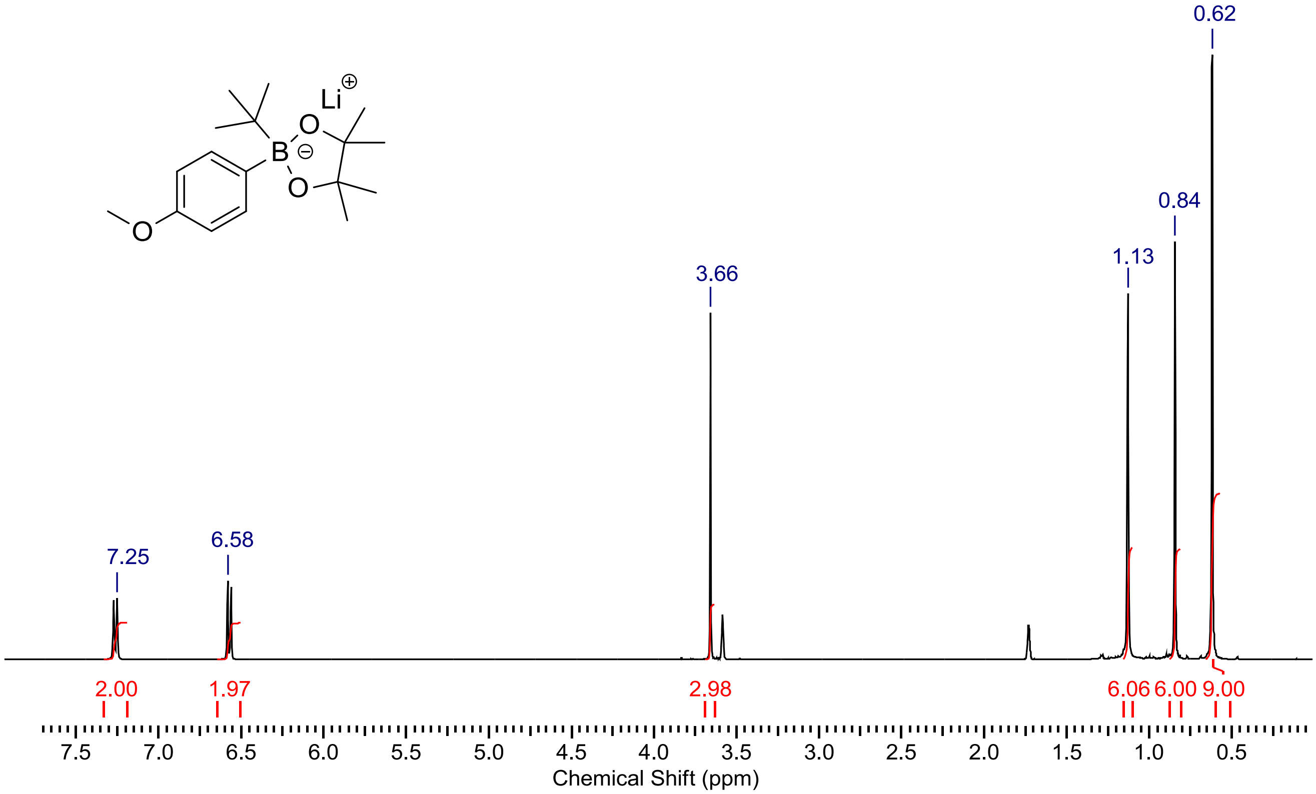

Figure S73: ^{1}H NMR (d$_8$-THF, 400MHz.)

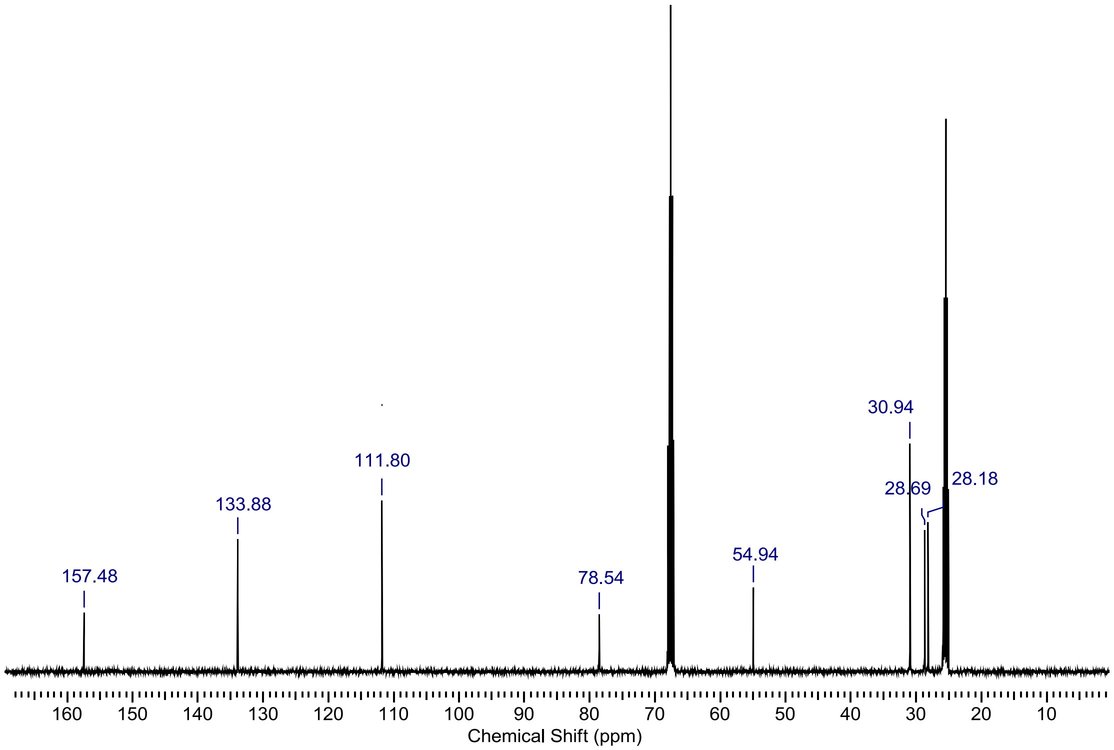

Figure S74: ^{13}C{^{1}H} NMR (d$_8$-THF, 101MHz)

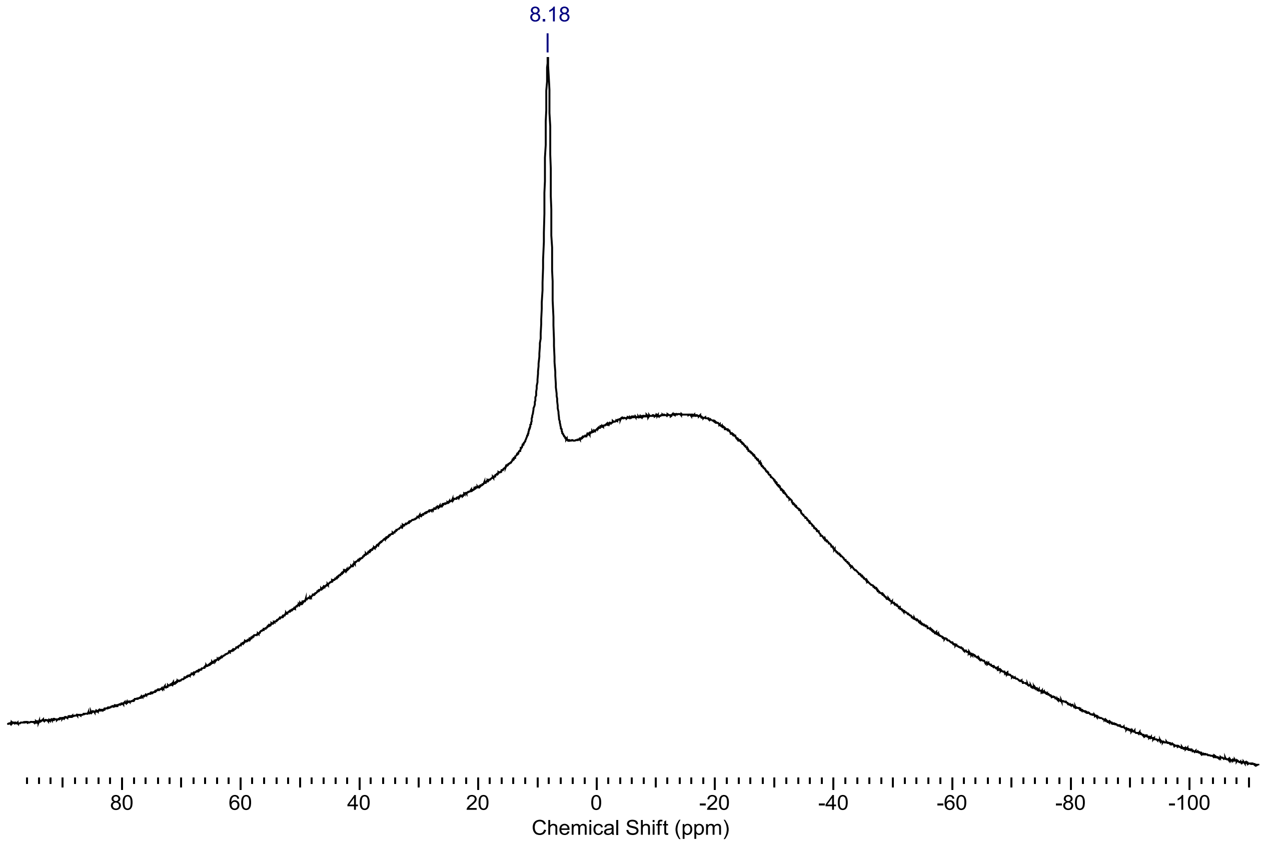

Figure S75: $^{11}B\{^{1}H\}$ NMR spectrum (d_8THF, 128 MHz)

1d

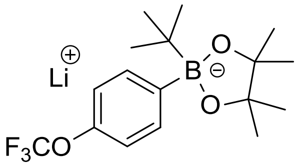

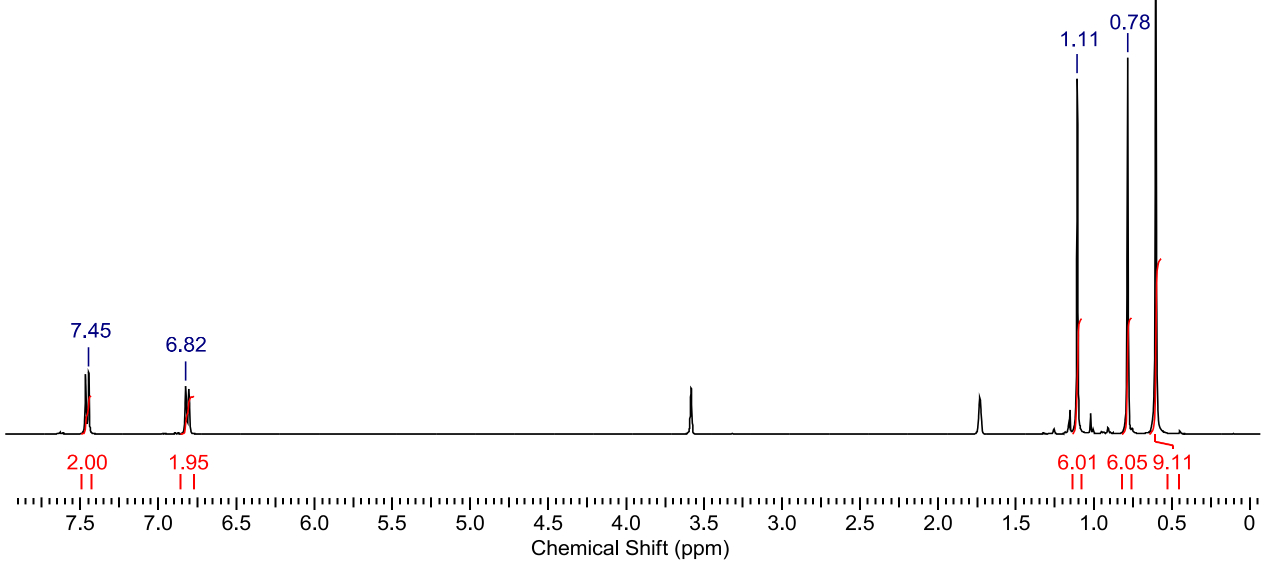

Figure S76: ^{1}H NMR (d$_8$-THF, 400MHz.)

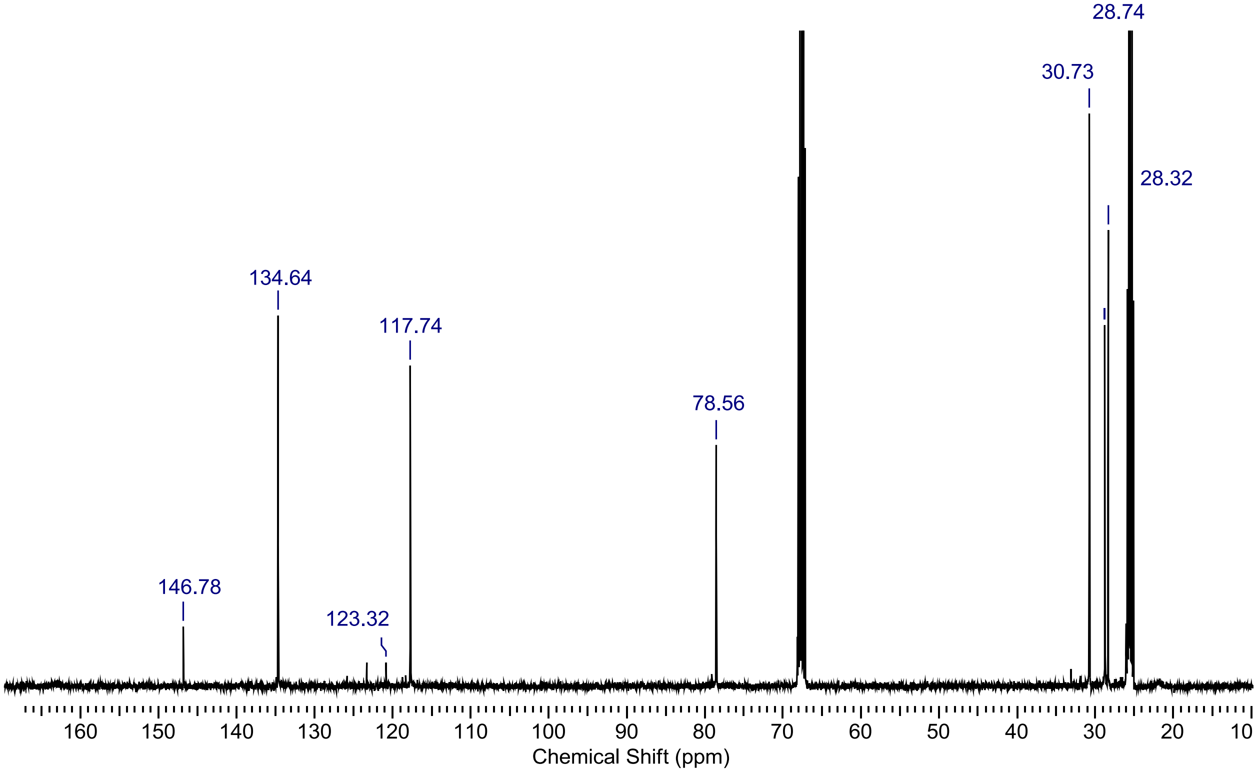

Figure S77: ^{13}C{^{1}H} NMR (d$_8$-THF, 101MHz)

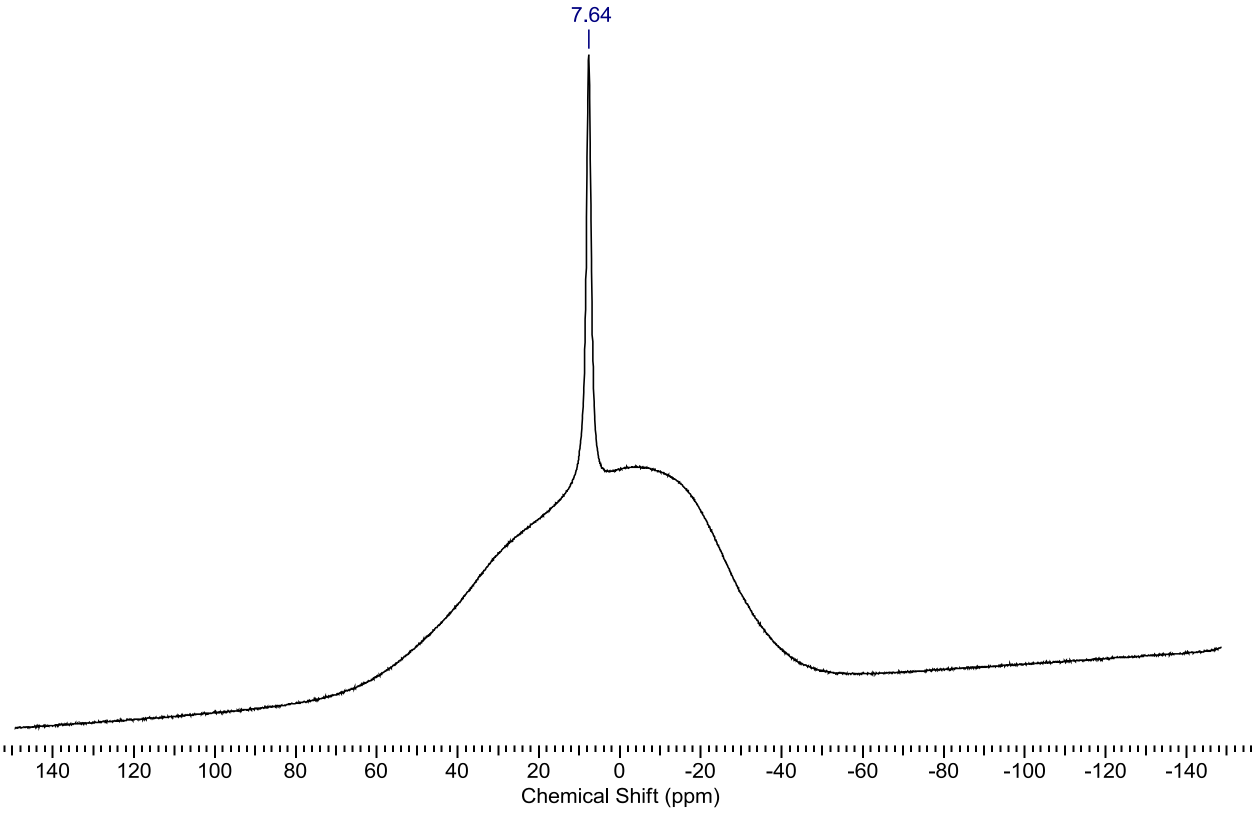

Figure S78: $^{11}B\{^1H\}$ NMR spectrum (d_8THF, 128 MHz)

Compound 6

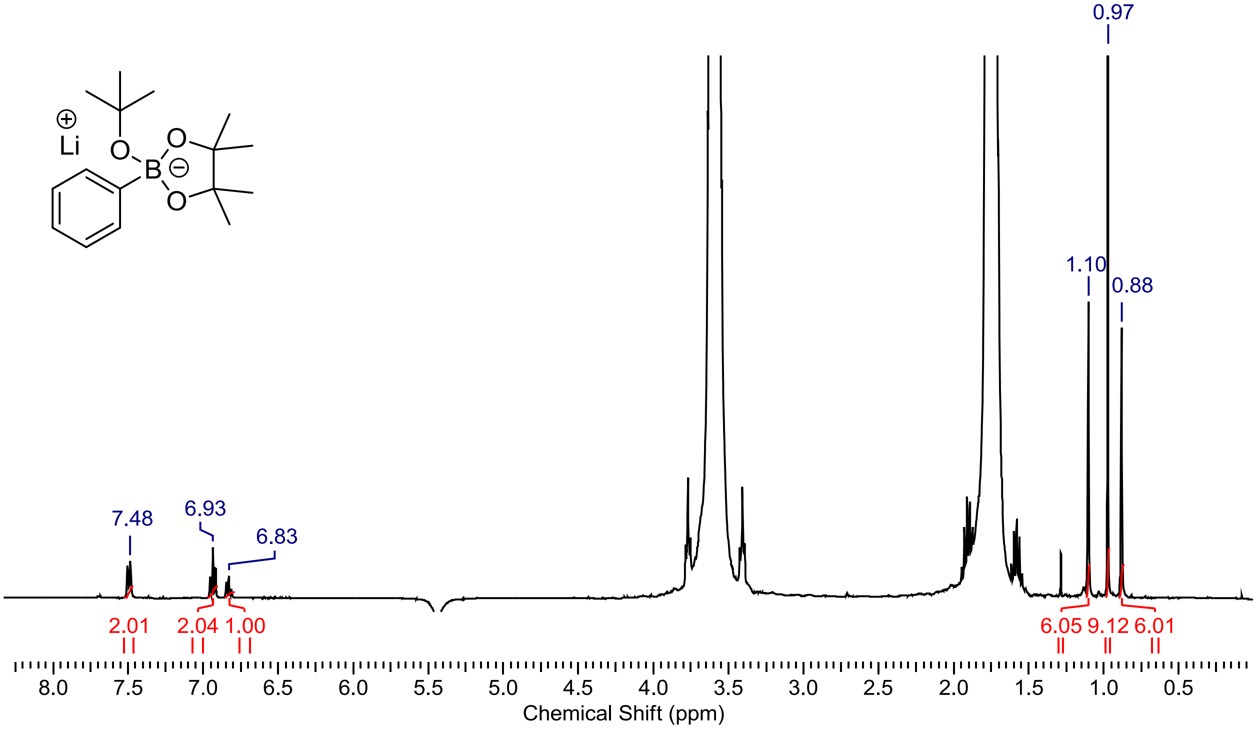

Figure S79: ^{1}H NMR spectrum(*protio*-THF, 400MHz)

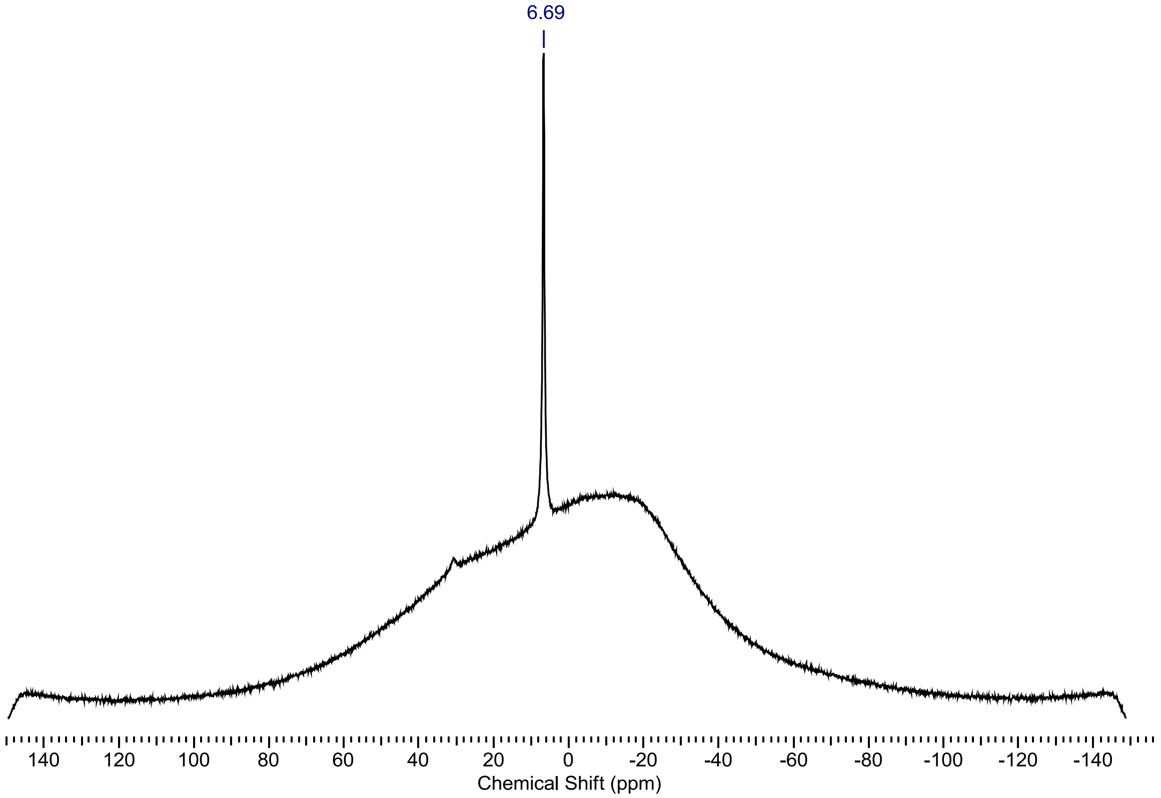

Figure S80: ^{11}B NMR spectrum (*protio*-THF) of 7

References

1. Hatakeyama, T.; Hashimoto, T.; Kondo, Y.; Fujiwara, Y.; Seike, H.; Takaya, H.; Tamada, Y.; Ono, T.; Nakamura, M., *J. Am. Chem. Soc.* **2010,** *132* (31), 10674-10676.
2. Dunsford, J. J.; Clark, E. R.; Ingleson, M. J., *Dalton Trans.* **2015,** *44* (47), 20577-20583.
3. Berionni, G.; Leonov, A. I.; Mayer, P.; Ofial, A. R.; Mayr, H., *Angew. Chem. Int. Ed.* **2015,** *54* (9), 2780-2783.
4. Bedford, R. B.; Brenner, P. B.; Carter, E.; Carvell, T. W.; Cogswell, P. M.; Gallagher, T.; Harvey, J. N.; Murphy, D. M.; Neeve, E. C.; Nunn, J.; Pye, D. R., *Chem. Eur. J.* **2014,** *20* (26), 7935-7938.
5. Bedford, R. B.; Gower, N. J.; Haddow, M. F.; Harvey, J. N.; Nunn, J.; Okopie, R. A.; Sankey, R. F., *Angew. Chem. Int. Ed.* **2012,** *51* (22), 5435-5438.
6. Pal, S.; Chowdhury, S.; Rozwadowski, E.; Auffrant, A.; Gosmini, C., *Adv. Synth. Catal.* **2016,** *358* (15), 2431-2435.
7. St. Denis, J. D.; Scully, C. C. G.; Lee, C. F.; Yudin, A. K., *Org. Lett.* **2014,** *16* (5), 1338-1341.
8. Fillion, E.; Beaton, E.; Nguyen, Y.; Wilsily, A.; Bondarenko, G.; Jacq, J., *Adv. Synth. Catal.* **2016,** *358* (21), 3422-3434.
9. Bedford, R. B.; Brenner, P. B.; Carter, E.; Clifton, J.; Cogswell, P. M.; Gower, N. J.; Haddow, M. F.; Harvey, J. N.; Kehl, J. A.; Murphy, D. M.; Neeve, E. C.; Neidig, M. L.; Nunn, J.; Snyder, B. E. R.; Taylor, J., *Organometallics* **2014,** *33* (20), 5767-5780.
10. Barroso, S.; Lemaire, S.; Farina, V.; Steib, A. K.; Blanc, R.; Knochel, P., *J. Org. Chem.* **2016,** *81* (7), 2804-2816.
11. Yuguchi, M.; Tokuda, M.; Orito, K., *J. Org. Chem.* **2004,** *69* (3), 908-914.
12. Gieshoff, T. N.; Chakraborty, U.; Villa, M.; Jacobi von Wangelin, A., *Angew. Chem. Int. Ed.* **2017,** *56* (13), 3585-3589.
13. Xia, Y.; Hu, F.; Liu, Z.; Qu, P.; Ge, R.; Ma, C.; Zhang, Y.; Wang, J., *Org. Lett.* **2013,** *15* (7), 1784-1787.
14. Sun, Y.-Y.; Yi, J.; Lu, X.; Zhang, Z.-Q.; Xiao, B.; Fu, Y., *Chem. Commun.* **2014,** *50* (75), 11060-11062.
15. Donck, S.; Baroudi, A.; Fensterbank, L.; Goddard, J.-P.; Ollivier, C., *Adv. Synth. Catal.* **2013,** *355* (8), 1477-1482.
16. Wehman, E.; Jastrzebski, J. T. B. H.; Ernsting, J.-M.; Grove, D. M.; van Koten, G., *J. Organomet. Chem.* **1988,** *353* (2), 133-143.
17. Hernán-Gómez, A.; Herd, E.; Uzelac, M.; Cadenbach, T.; Kennedy, A. R.; Borilovic, I.; Aromí, G.; Hevia, E., *Organometallics* **2015,** *34* (11), 2614-2623.
